# NANOG initiates epiblast fate through the coordination of pluripotency genes expression

**DOI:** 10.1038/s41467-022-30858-8

**Published:** 2022-06-21

**Authors:** Nicolas Allègre, Sabine Chauveau, Cynthia Dennis, Yoan Renaud, Dimitri Meistermann, Lorena Valverde Estrella, Pierre Pouchin, Michel Cohen-Tannoudji, Laurent David, Claire Chazaud

**Affiliations:** 1 Université Clermont Auvergne, CNRS, INSERM, GReD Institute, Faculté de Médecine, F-63000 Clermont-Ferrand, France; 2 Byonet, 19 rue du courait, F-63200 Riom, France; 3 Université de Nantes, CHU Nantes, INSERM, CR2TI, UMR 1064, ITUN, F-44000 Nantes, France; 4 Université de Nantes, CNRS, LS2N, CNRS UMR 6004, F-44000 Nantes, France; 5 Institut Pasteur, Université Paris Cité, CNRS UMR3738, Epigenomics, Proliferation, and the Identity of Cells, Department of Developmental and Stem Cell Biology, F-75015 Paris, France; 6 Université de Nantes, CHU Nantes, INSERM, CNRS, UMS Biocore, INSERM UMS 016, CNRS UMS 3556, F-44000 Nantes, France

**Keywords:** Differentiation, Stem-cell differentiation, Pluripotent stem cells

## Abstract

The epiblast is the source of all mammalian embryonic tissues and of pluripotent embryonic stem cells. It differentiates alongside the primitive endoderm in a “salt and pepper” pattern from inner cell mass (ICM) progenitors during the preimplantation stages through the activity of NANOG, GATA6 and the FGF pathway. When and how epiblast lineage specification is initiated is still unclear. Here, we show that the coordinated expression of pluripotency markers defines epiblast identity. Conversely, ICM progenitor cells display random cell-to-cell variability in expression of various pluripotency markers, remarkably dissimilar from the epiblast signature and independently from NANOG, GATA6 and FGF activities. Coordination of pluripotency markers expression fails in *Nanog* and *Gata6* double KO (*DKO*) embryos. Collectively, our data suggest that NANOG triggers epiblast specification by ensuring the coordinated expression of pluripotency markers in a subset of cells, implying a stochastic mechanism. These features are likely conserved, as suggested by analysis of human embryos.

## Abstract (web summary)

Pluripotent epiblast cells segregate from primitive endoderm in the blastocyst inner cell mass (ICM). Here the authors show that mosaic epiblast differentiation during mouse and human preimplantation development initiates stochastically in ICM progenitors, independently of the FGF pathway, and requires NANOG activity

## Introduction

During preimplantation development, the mammalian embryo develops into a blastocyst via two main differentiation events. First, from the 16-cell (16C) stage in the mouse, inner cells segregate from outer cells and form the inner cell mass (ICM), while the cells remaining outside will produce the extraembryonic trophectoderm (TE). The second differentiation event occurs in the ICM, with the production of epiblast (Epi), the source of all embryonic tissues and of embryonic stem (ES) cells, and primitive endoderm (PrE), which participates in the extraembryonic yolk sac formation, and is required for the proper patterning of the anteroposterior axis of the embryo and for primary haematopoiesis. At the beginning of blastocyst formation (20C-32C stage), ICM cells co-express NANOG and GATA6^1^. Then, between the 32C and 90C stage, these cells asynchronously differentiate into Epi cells that express preferentially NANOG, or into PrE cells that dominantly express GATA6, in an apparently random “salt and pepper” pattern^1–5^. FGF4 is secreted by Epi cells^3,4,6^ and converts unspecified neighbouring cells into PrE cells^2,5,7–15^. This short-range signal regulates precisely and robustly the proportion of Epi and PrE cells^16,17^ through positive and negative feedback loops^14,17–20^. Still, how Epi lineage is initiated remains unknown^21,22^. In this work, we establish that the Epi state is defined by the coordinated expression of Epi/pluripotency markers, which occurs between 16C and 32C stages. We also show that *DKO* embryos are unable to produce Epi or PrE cells that remain in an ICM progenitor-like state, revealing that NANOG is required for Epi initiation by enabling coordinated expression of pluripotency markers.

## Results

### Epi specification initiates between the 16C and 32C stages

**Fig. 1:**
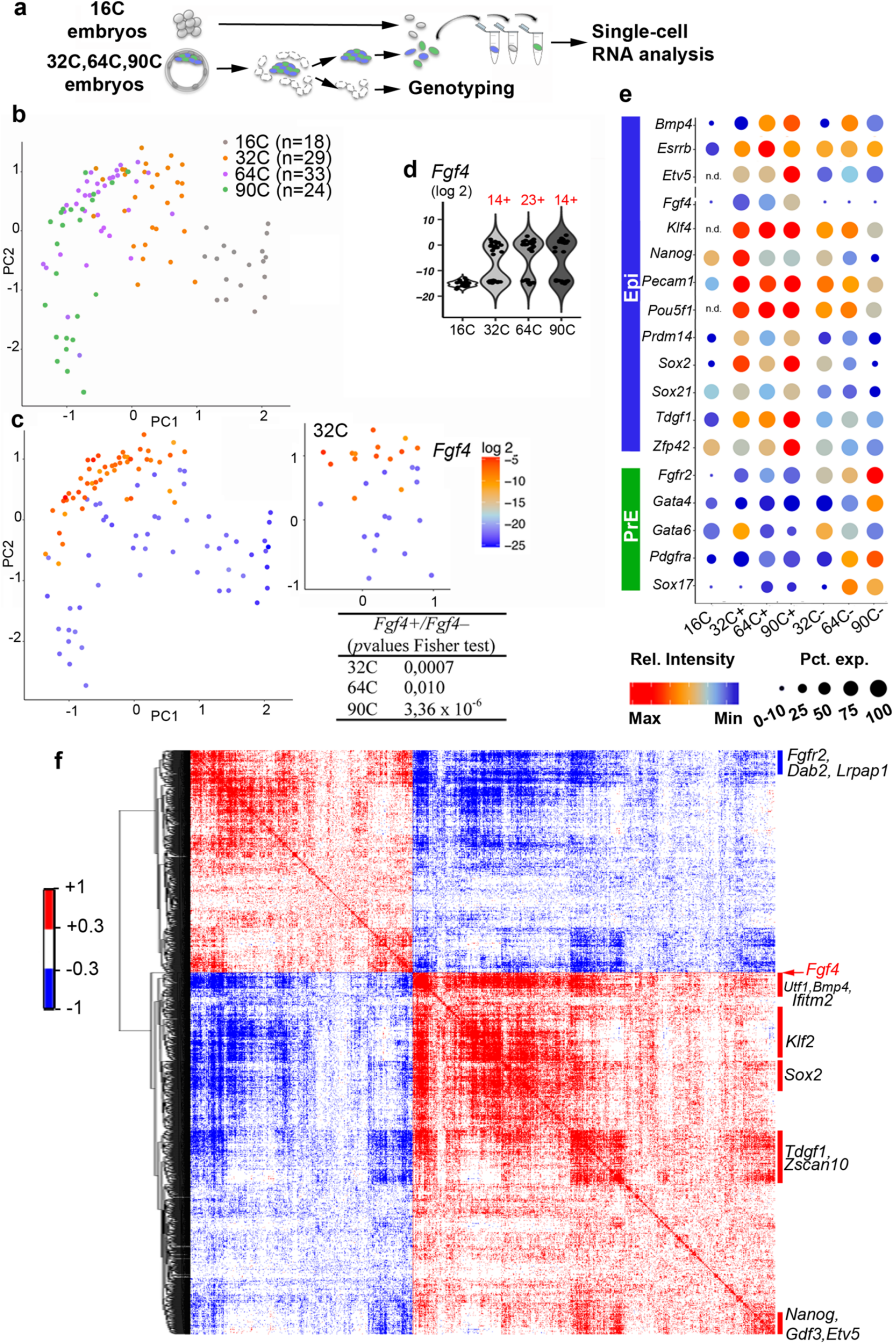
Emergence of two cell populations in 32C mouse embryos. **a** Single-cell isolation procedure for RT-qPCR analyses. **b**, PCA map where developmental stages are represented by the indicated colours (PC1 Score= 24.40%, PC2 score = 14.77%, PC3 score <0.01). **c** PCA plot shown in (**b**) with graded colours to indicate *Fgf4* expression level in each cell at the four stages (left panel) and at 32C only (right panel). The table shows the Fisher’s exact test values for *Fgf4*+ and *Fgf4*− cell distributions. **d** Violin plot of *Fgf4* expression level in individual cells. The number of *Fgf4* + cells at each stage is indicated in red. **e** Seurat plot showing the single-cell expression level of the indicated genes at the four stages in the *Fgf4*+ and *Fgf4*− populations, according to the violin plots shown in (**d**). The colour intensity represents the average expression level, whereas the dot size represents the proportion of cells that express the gene within the population (for each cell, values >0.10 of the gene maximal intensity were considered as positive). **f** Spearman correlation matrix for the paired expression of 1434 genes (selected by their correlation with *Fgf4* expression) in the 40 ICM cells at stage 32C. Genes are ordered in a hierarchical tree for similarity (see Supplementary data 4 for a detailed map in a vector-based PDF file). Source data are provided in the Source Data file.

To explore when and how ICM cells initiate their differentiation into Epi or PrE, we performed a quantitative single-cell gene expression analysis in wild type (*WT*) mouse embryos at the 16C, 32C, 64C, and 90C stages (i.e. from before to after their differentiation into Epi or PrE cells) using the Biomark Fluidigm system (Fig. 1a), as it offers a high sensitivity compared to other methods^23–25^. We compiled a list of genes that are known PrE and Epi markers^1–4,26,27^, of genes expressed before the 32C stage and/or linked to pluripotency in embryos or mouse (m)ES cells (e.g. *Zscan4*, *Tcfcp2l1*, *Sox21*, *Prdm14*)^28–31^, and of FGF pathway genes^9,27^. The progressive differentiation toward Epi and PrE cells was captured by principal component analysis (PCA) (Fig. 1b). The developmental time segregates cells along PC1, whereas PC2 highlights the differentiation between Epi and PrE, as indicated by the expression level of Epi and PrE markers (Supplementary Fig. 1a). We then assessed the expression dynamics of each gene at each stage (Fig. 1c; Supplementary Fig. 1b; Supplementary Data 1). Several Epi/pluripotency markers, such as *Fgf4*, *Prdm14*, *Klf2*, *Klf4* and *Tdgf1*, display heterogeneous expression in ICM cells from the 32C stage (Supplementary data 1). *Fgf4* is one of the first markers of binary differentiation^3,4,27,32^. Its expression is low at the 16C stage and increases from the 32C stage in a subset of cells, thus segregating the sample in two populations of *Fgf4*−positive (*Fgf4*+) and -negative (*Fgf4−*) (Fig. 1d). This enables to distinguish Epi and PrE cells at the 64C and 90C stages, confirmed by the expression of specific markers and in agreement with previous reports^3,9,27,32^ (Fig. 1e; Supplementary Data 2). Except for *Gata6* and *Fgfr2*, the analysed PrE markers start to be expressed later than Epi markers, between the 64C and 90C stage, as previously reported at the protein level^16,18,33^. At the 32C stage, *Prdm14*, *Klf2*, *Klf4*, *Tdgf1, Sox21, Sox2* and *Nanog* already display differential expression between *Fgf4*+ and *Fgf4−* cells, whereas other Epi/pluripotency markers, such as *Bmp4, Zfp42, Enox1* and *Esrrb*, become restricted to the Epi lineage only later (Fig. 1e; Supplementary Data 2).

*Fgf4*+ cells are already clustered on the PCA map at the 32C stage (Fig. 1c, right panel) indicating that *Fgf4* expression is not initiated randomly. Several Epi/pluripotency markers display a similar distribution (Supplementary Fig. 1b, c) and the *Fgf4*+ cells on the PCA map are mainly included in the *Nanog*+, *Prdm14*+*, Klf2*+*, Klf4*+ or *Tdgf1*+ populations (Supplementary Fig. 2a, b). Accordingly, *Fgf4* expression is significantly correlated with that of Epi/pluripotency genes in 32C embryos (Supplementary Table 1). Similar correlations are found with *Nanog* and *Sox2* expressions (Supplementary Table 2). Altogether, these results show that at the 32C stage, a group of cells share a gene expression signature that includes *Fgf4* and several pluripotency markers and that defines the Epi state. As the cell heterogeneities described previously at the 32C stage^27^ are not random, this questions how the coordinated expression of Epi markers is initiated. We thus examined genes expression in 16C embryos. At this stage, Epi markers display random expression (Supplementary Fig. 3, Table 2), indicating that the Epi state is not acquired yet. Thus, the presence of isolated Epi markers in undifferentiated cells is not indicative of an Epi state. The Epi state emerges between the 16C and 32C stages, attested by Epi genes expression coordination. Altogether, this analysis shows that in mouse embryos, ICM cells display two distinct cell heterogeneities. In undifferentiated cells Epi markers are expressed randomly, identifying a primary heterogeneity state. Conversely upon differentiation, cell heterogeneity is organised through the coordinated expression of Epi and PrE markers classically referred as the salt and pepper pattern.

We then extended our analysis to the whole transcriptome using published data on ICM annotated cells from 32C embryos^34^. The expression of 1434 genes is positively (888) and negatively (546) correlated with *Fgf4* expression level at the 32C stage (Supplementary data 3). These 1434 genes expression highly cross correlate, demonstrating a regulated organisation (Fig. 1f; Supplementary Data 4). This allowed enriching the Epi signature (Supplementary data 3). Among the positively correlated genes, *Nanog*, *Sox2*, *Bmp4*, *Klf2* and *Tdgf1* were also identified as 32C stage Epi markers with the Biomark Fluidigm analysis, thus validating this approach. The negatively correlated genes include *Fgfr2*, *Dab2* and *Lrpap1* that are known PrE markers^3,4,27,35^ (Fig. 1f).

We then examined whether the Epi expression correlation signature is already present in 16C stage identified inner cells^34^ when the first ICM cells are produced. At this stage, *Fgf4* is expressed in fewer cells and at lower levels compared with the 32C stage (Supplementary Fig. 4a). *Fgf4* expression is scarcely correlated with that of the 1434 genes in 16C stage inner cells (Supplementary data 3), and, importantly, cross correlations between these genes expression are almost inexistent (Supplementary Fig. 4b, Supplementary Data 5). This reveals the absence of the Epi signature at this stage and confirms that the Epi state emerges between the 16C and 32C stages. Thus our work demonstrates that two distinct ICM cell states are successively present in the developing blastocyst, designated as primary heterogeneity or salt and pepper heterogeneity. The transition from random to coordinated expression of Epi markers characterizes the production of Epi cells.

### *Nanog* is required to initiate Epi specification

**Fig. 2:**
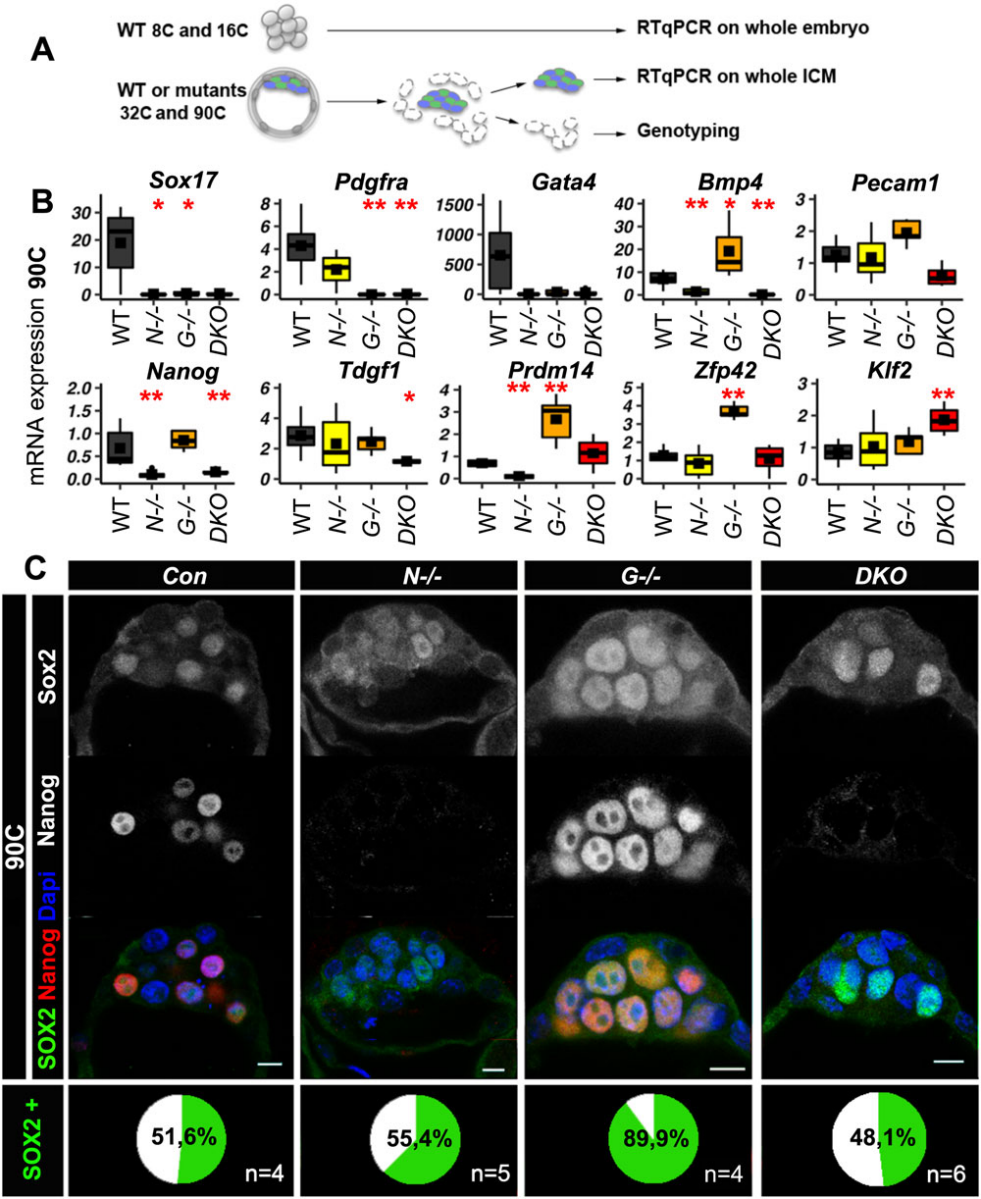
Absence of PrE and Epi differentiation in *DKO* embryos. **A** Schematic representation of the experimental procedure used in (**B**) and Supplementary data 6. For the 8C and 16C stages, individual whole embryos were analysed, whereas for the 32C and 90C stages individual whole ICMs were isolated by immunosurgery. **B** PrE and Epi gene expression analysis by RT-qPCR. Boxplots show the relative RNA levels in 90C *WT* (*n* = 6), *Nanog*^*−/−*^ (*n* = 6), *Gata6*^*−/−*^ (*n* = 5), and *DKO* (*n* = 6) whole ICMs. The mean is represented by a square, the median by the central line; *p < 0.05, **p < 0.01 (Two-sided Wilcoxon test relative to the *WT* sample, see Supplementary data 15 for exact *p* values). The edges of the box represent the 25th and 75th quartiles. The whiskers extend to 1.5 times the interquartile range (25th to 75th percentile). Source data are provided in the Source Data file. **C** Representative immunofluorescence confocal images of NANOG and SOX2 localization in 90C *Nanog*^*−/−*^ (*N*), *Gata6*^*−/−*^ (*G*), *DKO*, and control (*Con*) embryos. The percentage of SOX2-positive cells (SOX2+) in ICM is indicated in the bottom panel. Scale bars: 10 µm.

As *Gata6* and *Nanog* individual mutations prevent the binary specification of PrE and Epi, respectively^6,18,36–39^, we generated *Nanog*;^*−/−*^*Gata6*^*−/−*^ double KO (*DKO*) mouse embryos to capture the ICM molecular state before Epi and PrE differentiation initiation. After validating that TE and ICM cells are properly segregated (Supplementary Fig. 5a), we determined whether in *DKO* embryos, ICM cells could still differentiate by immunofluorescence (IF) analysis of PrE- and Epi-specific genes. RT-qPCR analysis of whole individual ICMs from *WT*, *Nanog*^*−/−*^*, Gata6*^*−/−*^, and *DKO* embryos at the 32C and 90C stages was also carried out (Fig. 2A). IF (Supplementary Fig. 5b, c) and RT-qPCR (Fig. 2B; Supplementary Data 6) show that PrE markers (e.g., *Sox17, Pdgfra, Gata4, Foxq1…*)^1,27,40^ are not expressed in *DKO* ICM samples. Some Epi markers (e.g. *Nanog, Bmp4*, *Tdgf1*) are significantly downregulated or absent in *DKO* ICMs at the 32C and 90C stages, while others show similar (*Zfp42, Prdm14*) or increased (*Klf2*) expression compared with *WT* ICMs (Fig. 2B; Supplementary data 6). *Nanog*^*βgeo*^ allele transcript levels, which can be detected in *Nanog* +*/*^*βgeo*^ embryos, are low or absent in *DKO* ICMs. This indicates that NANOG is required for its own expression in early blastocysts, in contrast to the self-repression reported in mES cells^41,42^ or at later stages (>120C)^43^. Overall, these findings indicate that *DKO* ICM cells cannot differentiate into PrE or Epi.

To further characterize the *DKO* ICM cell undifferentiated state, we examined the expression of the pluripotency markers SOX2 and PECAM1 that are normally detected only in 16C inner cells and then become progressively restricted to Epi cells at the 90C stage^44–48^. SOX2 and PECAM1 are expressed in *DKO* embryos at the 32C and 90C stages (Fig. 2C; Supplementary Fig. 5d). SOX2 displays cell-to-cell heterogeneous nuclear levels, indicating that *Sox2* initial expression is induced independently of NANOG and GATA6 presence. This heterogeneous pattern is lost in *Gata6*^*−/−*^ embryos, where all ICM cells differentiate into Epi cells. This indicates that in these embryos, SOX2 expression is influenced by NANOG expression that is present in all ICM cells. Thus, SOX2 expression is controlled by different inputs that are first NANOG-independent and later NANOG-dependent. This is confirmed by SOX2 downregulation in E4.25 (>120C) *Nanog*^−/−^ embryos^43^. To further evaluate the heterogeneous expression of pluripotency transcription factors, we assessed KLF4 and SOX21 levels that show cell-to-cell variability during mouse preimplantation^30,49^. IF analysis shows similar KLF4 expression patterns in the ICM of *DKO* and *WT* embryos (Supplementary Fig. 5e). Compared with earlier stages, SOX21 levels are decreased in *WT* 90C ICM cells (Supplementary Fig. 5f). This result is in agreement with undetectable SOX21 in mES cells^50,51^ that correspond to late blastocyst Epi cells^52^. In *DKO* ICMs, SOX21 displays cell-to-cell heterogeneity (Supplementary Fig. 5f), indicating the maintenance of the early expression pattern. Altogether IF analysis of SOX2, KLF4 and SOX21 suggests that in the absence of NANOG and GATA6, ICM cells remain in a progenitor state.

**Fig. 3:**
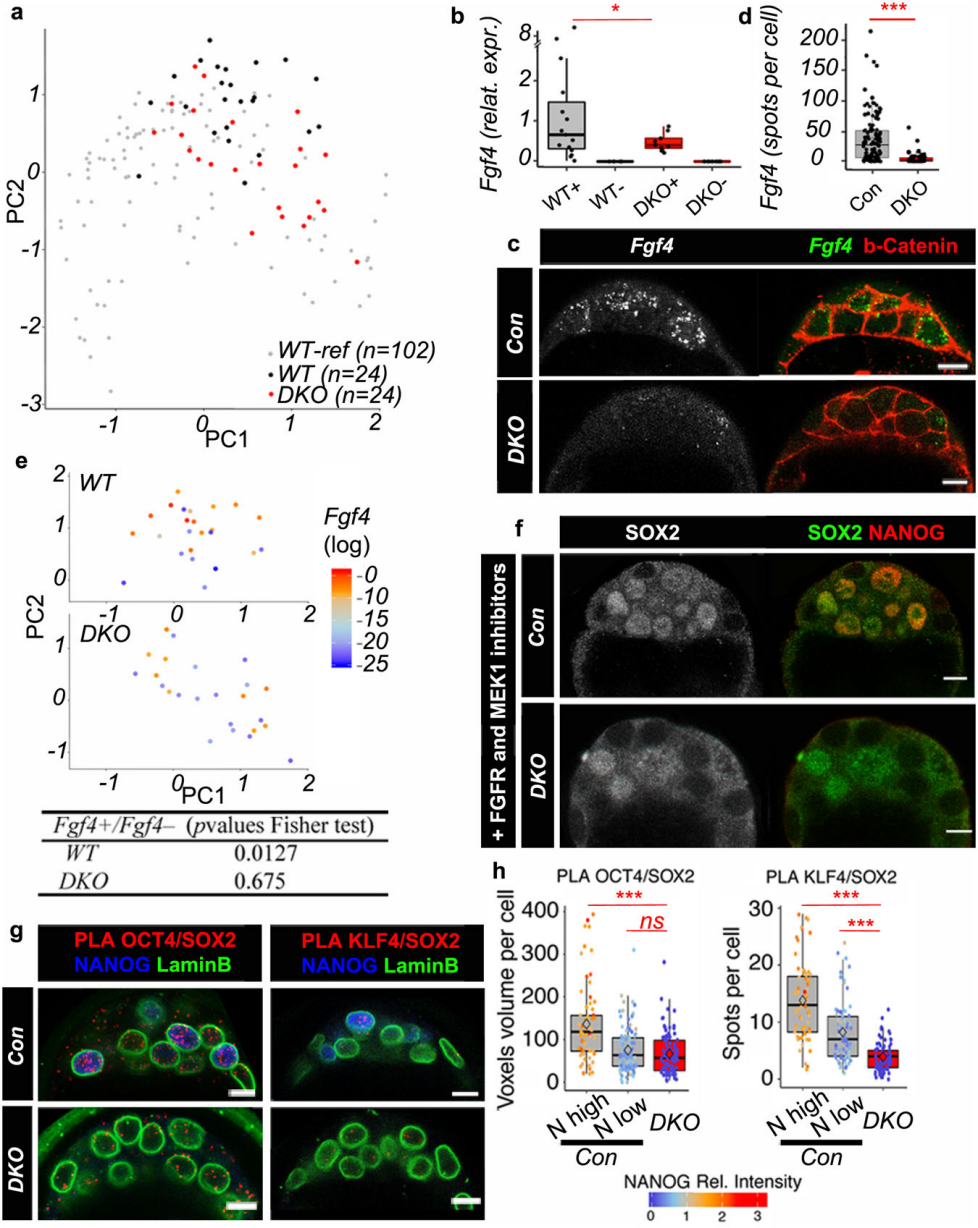
Cell heterogeneity in *DKO* ICMs. **a** PCA performed with *DKO* and *WT* cells (32–50C) and with *WT* cells from Fig. 1a, b (16C, 32C, 64C and 90C) as reference (*WT*-Ref) (scores: PC1, 20.84%; PC2, 13.13%). **b** Boxplot showing *Fgf4* expression levels in *Fgf4*+ and *Fgf4*− populations (+ and *−*) in *WT* (*n* = 4 embryos) (15 cells *Fgf4*+ and 9 cells *Fgf4-)* and *DKO* (*n* = 4) (9 cells *Fgf4*+ and 15 cells *Fgf4*−); Fligner-Killeen test for homogeneity of variance (between *WT* and *DKO Fgf4*+ cells *p* value = 0.012. **c** Representative images of *Fgf4* HCR RNA-FISH, coupled with b-Catenin IF, in 64C *DKO* (*n* = 4) and control embryos. Scale bars: 10 µm. **d** Quantification of HCR RNA-FISH spots in ICM cells from control (*n* = 115 cells from 7 embryos) and *DKO* (*n* = 77 cells from 4 embryos) embryos (Fligner-Killeen test *p* value < 2.2 e^−16^). **e** PCA map from (**a**) with *WT* (top panel) and *DKO* (bottom panel) cells only, and with graded colours indicating *Fgf4* expression level. Fisher’s exact test p-values (bottom table). **f** Representative immunofluorescence images of 64–90C control and *DKO* (*n* = 6) embryos cultured with FGFR and MEK1 inhibitors showing SOX2 and NANOG localization. Scale bars: 10 µm. **g** Representative images after SOX2/OCT4 and SOX2/KLF4 PLA, coupled with LaminB and NANOG immunofluorescence in control and *DKO* embryos (*n* = 4 for SOX2/OCT4; *n* = 4 for SOX2/KLF4). Scale bars: 10 µm. **h** Quantification of PLA signals per nucleus from (**g**). N high, cells with high NANOG expression; N low, cells with low NANOG expression. For SOX2/OCT4: *n* = 65 N high cells, *n* = 102 N low cells and *n* = 91 *DKO* cells. For SOX2/KLF4: *n* = 62 N high cells, *n* = 80 N low cells and *n* = 79 *DKO* cells. Pairwise Wilcoxon comparisons after a two-sided Kruskal-Wallis test of the three cell populations (***p < 0.01; ns=not significant; exact *p*-values: for OCT4/SOX2 between N high and DKO cells *p* = 4.0 e^−08^; for OCT4/SOX2 between N low and DKO cells *p* = 0.15; for KLF4/SOX2 between N high and DKO cells *p* < 2.2 e^−16^; or KLF4/SOX2 between N high and DKO cells *p* = 4.4 e^−10^. For boxplots: The edges of the box represent the 25th and 75th quartiles. The median is represented by the central line. The whiskers extend to 1.5 times the interquartile range (25th to 75th percentile). Cells are plotted individually. Source data are provided in the Source Data file.

To further characterize the identity of *DKO* ICM cells, we carried out single-cell RNA analysis, as described in Fig. 1a. We focused on single and double mutant embryos at stage 32–50C, because this is the time of appearance of *Fgf4*+ cells and of Epi specification (Fig. 1). Overall, *WT* and *DKO* cells are interspersed along the PC1 axis (Fig. 3a; see also Supplementary Fig. 6 for PCA analyses of the four genotypes), indicating no major delay in *DKO* embryo development. *Lats2, Socs3* or *Zfp281* that are expressed preferentially early around the 16C stage in *WT* cells, maintain a high expression in 32C *DKO* embryos (Supplementary Fig. 7). This confirms that *DKO* ICM cells remain in an early undifferentiated state. We detected a subset of *Fgf4*+ cells also in *DKO* embryos, but *Fgf4* range of expression is strongly reduced compared with *WT Fgf4*+ cells (Fig. 3b). A similar decrease of expression is observed by RNA-FISH (Fig. 3c, d). Thus, NANOG is required for high *Fgf4* expression in individual cells and efficient activation of the pathway, as illustrated by the absence of cytoplasmic phosphorylated ERK and nuclear DUSP4 signal^7^ in *DKO* ICMs (Supplementary Fig. 5g, h). *Fgfr1* and *Fgfr2* expression levels are similar in *DKO* and *WT* ICMs (Supplementary data 7), strongly suggesting that the lack of ERK pathway activation is only due to low FGF4 levels. The existence of a threshold level of FGF4 activity is supported by data showing that incremental doses of recombinant FGF4 are required for PrE differentiation in *Fgf4* mutants^8,10,17^. Moreover, ERK differential activity correlates with the number of FGF4-secreting Epi cells^14^. Together, our results indicate that NANOG is necessary to boost *Fgf4* expression in Epi cells to the levels required for FGF pathway activation in neighbouring cells. In agreement, NANOG can bind to the *Fgf4* locus in mES cells^53,54^. Thus, the FGF pathway seems to act only downstream of Epi specification.

In line with the whole ICM analysis at 32C, many analysed Epi genes are still expressed in *DKO* cells (Supplementary data 7). However, unlike *WT* cells, *Fgf4*+cells in *DKO* embryos are scattered on the PCA map (Fig. 3e). Moreover, the correlations between Epi genes expressions are globally lost (Supplementary Table 3). These two features are characteristic of the primary heterogeneity state observed at 16C in *WT* embryos. Altogether, our data indicate that NANOG is required for the coordinated expression of pluripotency genes to enable the emergence of the Epi state.

Although *Fgf4* level was lower in individual cells of *DKO* than of *WT* embryos, we cannot rule out that it may still create some heterogeneity among ICM cells. To test this, we cultured 8C to 90C *DKO* embryos in the presence of FGFR and MEK inhibitors, as previously described^6,12,15^. In these conditions, SOX2, SOX21 and KLF4, still show cell-to-cell variability in *DKO* ICM cells (Fig. 3f; Supplementary Fig. 8). This demonstrates that gene expression in ICM cells remains heterogeneous in the absence of *Nanog*, *Gata6* and the FGF/MEK pathway. Thus, ICM progenitor cell heterogeneity exists before NANOG, GATA6 and FGF pathway become effective.

### NANOG promotes interactions between Epi-specific factors

Our results show that between 16C and 32C, a subset of ICM progenitor cells change their identity to become Epi. This important step requires the presence of NANOG, however NANOG alone does not seem to be sufficient to induce *Fgf4* expression. Indeed, despite high levels of NANOG protein in a subset of 16C cells^55^ (our unpublished results), *Fgf4* expression level is barely detectable at this stage^3^ (Supplementary data 1, 2). In addition, such *Nanog*+ *Fgf4-* cells can still be found at the 32C stage (Supplementary Fig. 2b). This suggests that other factor(s) collaborate(s) with NANOG to induce *Fgf4* expression. SOX2 and OCT4 cooperate with NANOG on enhancers to maintain mES cell pluripotency^53,56–58^. They can also synergistically induce *Fgf4* expression through a shared binding site^45,59^. Besides SOX2 and OCT4, KLF4 also is involved in pluripotency acquisition and maintenance^60^, and physically interacts with SOX2, OCT4 and NANOG in mES cells^61,62^. These transcription factors are considered as pioneer factors to open chromatin and initiate lineage differentiation^63,64^, and therefore, are good candidates to induce Epi specification. Our results show that different Epi factors are already transcribed at the 16C stage, and despite their uncorrelated expression at this stage, they can be co-expressed with *Nanog* in some cells, therefore randomly. We thus hypothesized that OCT4, SOX2, KLF4 cooperate with NANOG to initiate the Epi state. Indeed, these factors are present before Epi specification^48,49,55^ and are expressed in *DKO* embryos (Fig. 2, Supplementary Fig. 5, 7), thus independently from NANOG expression. Consequently, they should still be able to interact with each other in *DKO* ICMs. We analysed potential interactions by Proximity Ligation Assay (PLA) to obtain single-cell resolution in preimplantation embryos^65,66^. In 64–90C embryos NANOG/SOX2 PLA foci are present and enriched in nuclei with high NANOG levels, and are absent in *Nanog*^*−/−*^ embryos, used as negative control (Supplementary Fig. 9). PLA experiments with SOX2/KLF4 and SOX2/OCT4 also show foci enrichment in nuclei of *WT* cells with high NANOG expression (Fig. 3g, h). In *DKO* ICMs, the number of SOX2/KLF4 and SOX2/OCT4 foci was significantly reduced (Fig. 3g, h). Thus, in the absence of NANOG and GATA6, the number of interactions between these transcription factors is decreased and coincides with the lack of Epi differentiation. This indicates that NANOG is required for maximal interactions between these pioneer factors.

### Mouse and human Epi specification share conserved features

**Fig. 4:**
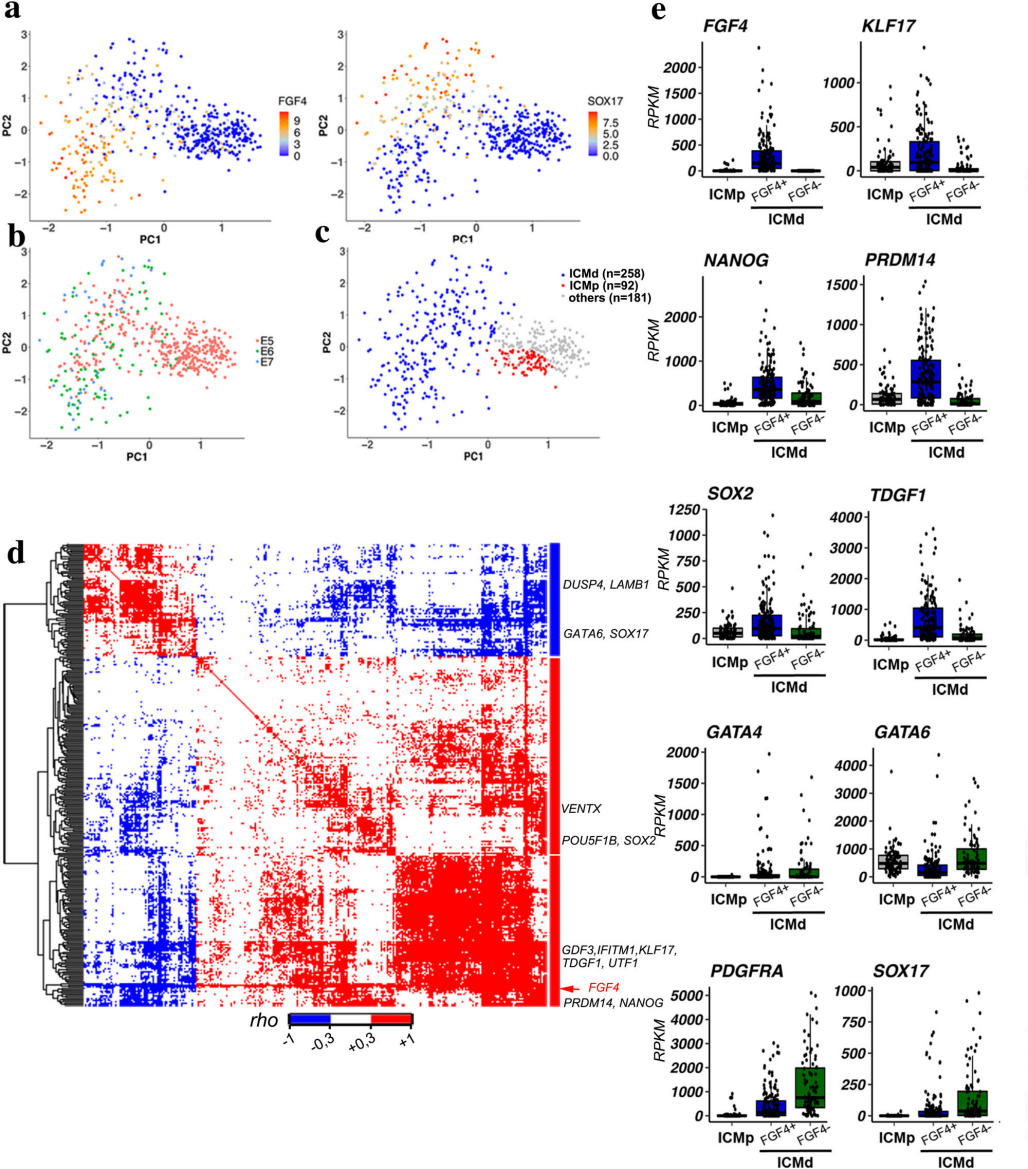
Single-cell RNA sequencing data analysis in human ICMs. **a** PCA map of human cells from 52 embryos with graded colours indicating *FGF4* and *SOX17* expression levels in E5 cells and E6-E7 ICM cells (PC1 Score= 28.09%, PC2 score = 11.34%, PC3 score = 6%). **b** Same PCA plot as in (**a**) where developmental stages are represented by the indicated colours. **c** Same PCA plot showing ICMd and ICMp cells (see Supplementary data 8 for the list of cells and their allocation). **d** Spearman correlation matrix for paired expression of the 308 *FGF4*-correlated genes in the 258 human ICMd cells. Genes are ordered in a hierarchical tree for similarity (see Supplementary data 9 for detailed map in the vector-based PDF file). **e** Single cell normalized expression levels of Epi and PrE markers in ICMp (*n* = 88) and ICMd (*FGF4*+ (*n* = 172) and *FGF4*- (*n* = 86)) cells (Supplementary data 8); Two-sided Wilcoxon test between *FGF4*+ and *FGF4-* ICMd cells (*p*-values: *FGF4* *p* < 2.00 e^−16^; *KLF17* *p* = 5.80 e^−11^; *NANOG* *p* = 5.40 e^−09^; *PRDM14* *p* < 2.00e^−16^; *SOX2* *p* = 6.10^e-08^; *TDGF1* *p* = 2.40 e^−13^; *GATA4* *p* = 0.38; *GATA6* *p* = 3.10 e^−12^; *PDGFRA* *p* = 1.40 e^−11^; *SOX17* *p* = 8.00 e^−08^). For boxplots: The edges of the box represent the 25th and 75th quartiles. The median is represented by the central line. The whiskers extend to 1.5 times the interquartile range (25th to 75th percentile). Cells are plotted individually.

While initiation of the TE programme in human embryos has been recently unveiled^67^, how Epi and PrE cells differentiate in this species remains an open question. Like in mice and other mammals^68^, NANOG and GATA6 show a mutually exclusive expression in human Epi and PrE cells, respectively, at the late blastocyst stage^69–72^. However, modulating the FGF/ERK pathway seems to have minimal effects on Epi/PrE specification in human embryos^70,72,73^, suggesting that other pathways could be involved conjointly or independently. We investigated whether in human embryos Epi cell induction is characterized by a transition from uncorrelated to correlated expression of Epi genes, as we found in the mouse. We analysed four published single-cell RNA-seq datasets^74–77^ to characterize ICM cell expression profiles before and during Epi and PrE specification in 54 E5 to E7 embryos. ICM/TE cell fate and commitment from the morula stage have not been fully investigated in humans; however, transcriptomic analyses allow discriminating cells with TE, Epi and PrE signatures^75^. In these datasets, TE cells at E6 and E7 have been clearly identified^74,75,77^ and were removed. We analysed all cells from E5 embryos to include all potential ICM progenitor cells, leading to a total of 518 cells examined (Supplementary data 8). To map Epi and PrE lineage specification, we followed the expression of the human orthologues of the 36 informative genes identified in the mouse. This strategy is supported by previous reports demonstrating that a minimal set of markers can allow a clear lineage separation^75^. To be able to discriminate TE cells in E5 embryos, we added TE markers such as TACSTD2, ENPEP or ABCG2^74^ to the list. Using the PCA approach, we found that these markers segregate also human Epi and PrE cells, as indicated by the expression of *FGF4* and *SOX17*, respectively (Fig. 4a). Other known Epi (*KLF17, TDGF1, SOX2*) and PrE (*GATA4, PDGFRA*) markers behave similarly (Supplementary Fig. 10a, b). K-means clustering gives a similar pattern (Supplementary Fig. 11a). PCA analysis of the data at E5, E6 and E7 shows that cells are interspersed, as previously observed^74^, indicating asynchronous cell specification (Fig. 4b). Consequently, the embryonic day cannot be used to discriminate progenitors from differentiated cells. We then asked whether an ICM progenitor cell state, equivalent to the mouse 16C inner cells, is present in early human blastocysts. K-means clustering identifies a cluster of early cells that segregates from the Epi and PrE clusters on the PCA (Supplementary Fig. 11a, cluster 3). This cluster contains early ICM cells before their differentiation into Epi or PrE, as well as early TE cells. We then discarded the clusters of cells that express TE markers, such as *TACSTD2*, *ENPEP* and *GATA2/3*^67,74,78^ (Supplementary Fig. 11b–d). We also removed 14 cells originating from embryos in which no TE cell was identified, and thus possibly representing earlier embryos in which TE/ICM differentiation has not occurred yet. This led to the identification of 88 ICM progenitor cells among which 30 were defined as “early ICM” in previous reports^74,75^. Altogether, our analysis highlights the presence of two ICM cell populations: 1) unspecified progenitor cells (ICMp), and 2) cells that have started or completed differentiation (ICMd) into Epi or PrE (Fig. 4c; Supplementary data 8). The expression of 308 of the 34 K expressed genes is correlated positively or negatively with *FGF4* expression in the ICMd population (Fig. 4d, Supplementary data 3, Supplementary data 9). The list of positively correlated genes includes Epi/pluripotency markers (e.g. *NANOG, IFITM1, PRDM14, TDGF1, GDF3, SOX2, KLF17*) in human embryos and human ES cells^74,75,79^. Their expression is enriched in *FGF4*+ cells (Fig. 4e). Correlation analyses with *NANOG* expression also highlight known Epi/pluripotency markers (Supplementary Fig. 12a, Supplementary data 3, 10). The expression of 56% of the 308 *FGF4*-correlated genes is also correlated with *NANOG* expression (Supplementary Data 3). By comparing these 308 genes with the mouse genes, we established a list of 44 genes that defines a common Epi expression correlation signature in both species (Supplementary data 3). In ICMp cells, genes belonging to the 308 genes, such as *SOX2, PRDM14, TDGF1, KLF17, NANOG*, show cell-to-cell expression variability (Supplementary Fig. 10); however, most of the correlations found in the ICMd matrix are absent in the ICMp population (Supplementary Fig. 12c; Supplementary data 12). We found a similar absence of correlation in ICMp cell when comparing with NANOG expression Supplementary Fig. 12b; Supplementary data 3, 11). In addition, we also analysed a list of genes defined as Epi specific by Stirparo et al^75^. that was also produced from the Petropoulos et al^74^. dataset. To analyse all ICM cells from E5 to E7 stages, we compiled the Epi and PrE modules from their WGCNA analysis, merged with their list of mature Epi markers identified by PCA, leading to 1065 genes (Supplementary data 3). Paired gene expression correlation matrices were produced (Supplementary Fig. 12 d, e). While a tight cluster of Epi markers is segregated in ICMd cells, Epi and PrE markers are scattered all along the tree built with ICMp cells. These results show that expression correlation analyses allow the identification of the three cell states of human ICM: progenitors, Epi, and PrE. Collectively, our results demonstrate that in both mouse and human ICM progenitors, cell-to-cell random expression of some Epi/pluripotency genes defines the primary ICM heterogeneity. Epi differentiation is initiated through the coordinated expression of several Epi/pluripotency genes in individual cells within the ICM cell population in an asynchronous manner. Correlation analysis of gene expression in mouse and human ICM cells suggests that Epi specification could initiate through a conserved stochastic mechanism.

## Discussion

Here, we report the characterization of two states of cell heterogeneity that define two phases of mammalian ICM development. In undifferentiated progenitor cells, uncoordinated expression of some Epi genes characterizes the primary ICM cell heterogeneity. Conversely, the well described Epi/PrE cell heterogeneity (“salt and pepper”) observed in intermingled but specified cells exhibits coordinated expression of either Epi or PrE markers. Thus, the acquisition of the Epi state is defined by the onset of the coordinated expression of Epi markers previously showing random cell-to-cell variability, as well as by the upregulation of other specific markers. Two phases of cell heterogeneity were delineated in the mouse before^27^, but our results indicate an earlier transition start between the 16C and 32C. The cell-to-cell variability with characteristics of ICM primary heterogeneity observed in *DKO* embryos (lacking NANOG and GATA6, the main players of Epi/PrE cell heterogeneity) validates the existence of an ICM primary heterogeneity before Epi and PrE differentiation, and demonstrates that NANOG is required to initiate Epi cell differentiation. This mirrors NANOG requirements during iPSC reprogramming to the pluripotent state^39^, while its presence is less important for maintaining mES cell pluripotency^80^. As the absence of NANOG in *DKO* embryos reduces the number of interactions between the pluripotency factors SOX2, OCT4 and KLF4, we propose that NANOG enables the association of pluripotency factors to induce the Epi state. Whether NANOG directly initiates these interactions in vivo or through the activation of the pluripotency network, or both, is not known yet. In mES cells it was shown that NANOG drives the recruitment of OCT4 and SOX2 at transcriptionally active sites^57^. In the future, it will important to determine whether NANOG recruitment activity is critical not only when the Epi-like state is already settled like in mES cells but also for initiation of Epi fate in embryos.

**Fig. 5:**
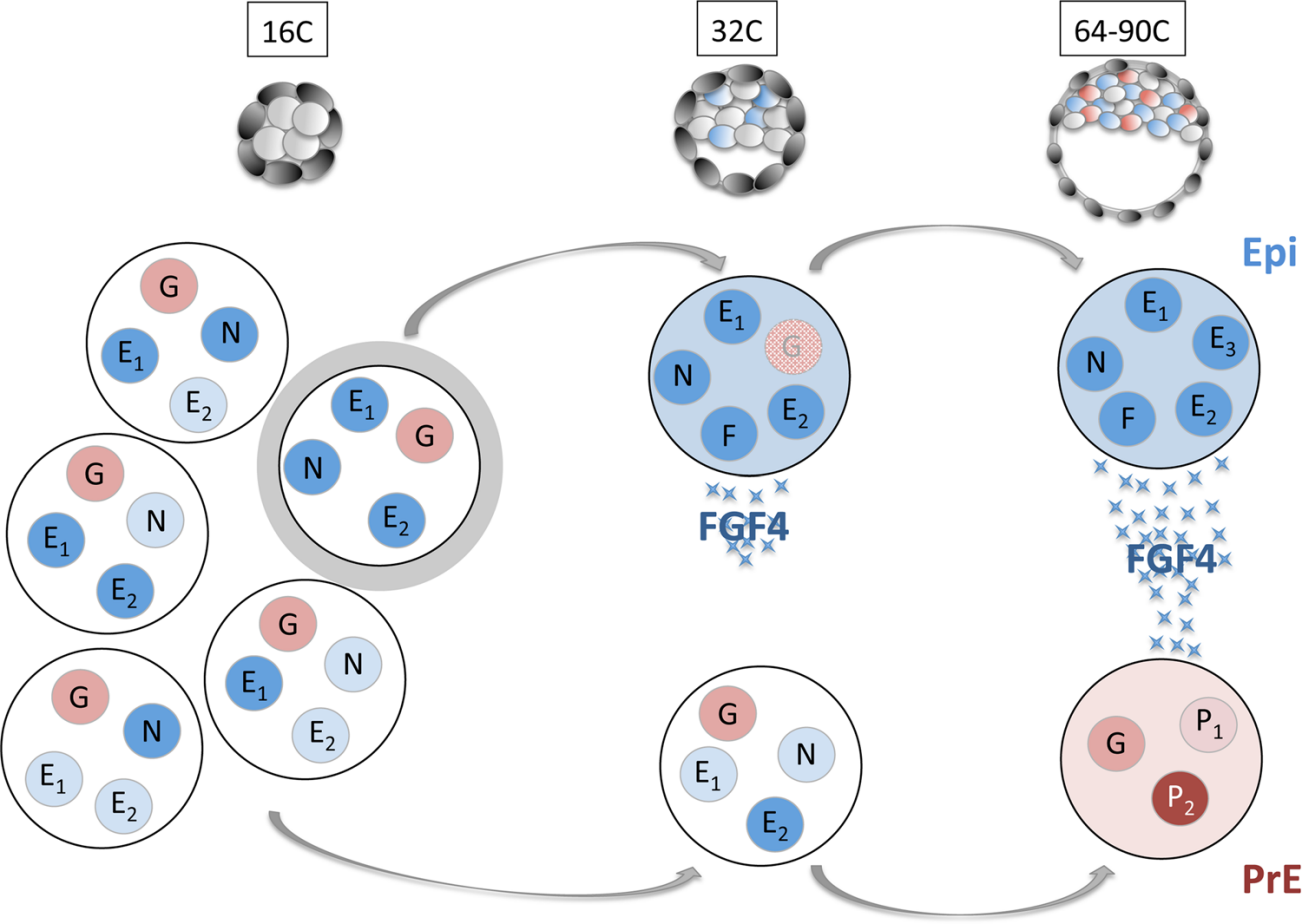
Schematic model of Epi differentiation. At the 16C stage, all ICM progenitor cells express various Epi genes (blue, E1, E2…) including NANOG (N), at different levels (colour intensity) without correlation. GATA6 (G) is expressed at similar levels in all cells. Some cells (highlighted in grey) randomly co-express NANOG with other Epi factor(s). Only in these cell(s), the co-expression promotes the coordinated expression of Epi factors that induces the Epi state at the 32C state and boosts *Fgf4* expression (F). After the 32C stage, FGF4 is strongly secreted by Epi cells, enabling the differentiation of their undifferentiated neighbours into PrE cells that express specific PrE markers (red, P1, P2).

Collectively, our results and previous work in the mouse^5,6,27,81^ lead us to propose the following mechanism of Epi and PrE differentiation (Fig. 5). At the 16C stage, ICM progenitor cells display the primary cell-to-cell heterogeneity characterized by the uncorrelated expressions of some Epi/pluripotency markers, independently of NANOG, GATA6 and FGF pathway activities. Then, NANOG orchestrates Epi specification by recruiting Epi factors such as SOX2, OCT4 and KLF4, at target Epi genes like *Fgf4*. For this, these factors need to be expressed in the same cell. As *Nanog* and Epi factors are initially randomly expressed, we propose that the stochastic co-expression of NANOG with one or more Epi factors activates the pluripotency network in these cells. This results in the coordinated expression of Epi/pluripotency markers characterizing Epi specification. The precise mechanism is not known yet. Does NANOG directly recruit TFs at binding site, or does it potentiate pioneer factors already present as shown in other chromatin regulations^82^? NANOG is absolutely required for this step. Conversely, other Epi factors might display some redundancy, as exemplified by the correct Epi specification in *Sox2* and *Oct4* single KO embryos^48,83,84^. First, only a subset of cells differentiates into Epi^16,18,33^, thus allowing the differentiation of other unspecified cells into PrE^5,17,33^. PrE cells are designated through this stochastic mechanism, but their differentiation also has a deterministic component driven by the FGF pathway.

Cell-to-cell variability of several Epi transcript levels in ICM progenitor cells can depend on multiple, non-exclusive mechanisms, such as transcriptional noise, uneven cell partitioning of RNA or proteins leading to differential transcription factor kinetics and nuclear presence, asynchronous cell cycle and chromatin remodelling^21,81,85,86^. Increase of transcriptional noise has been observed between the 8C and the blastocyst stage^87^, at the onset of ICM differentiation, and an increase in cell heterogeneity precedes cell specification in other systems^88–90^. At the 16C stage, *Nanog* locus displays low transcriptional activity (Supplementary data 2, Supplementary Fig. 4a), illustrated by random monoallelic pulses^91–93^. As translation occurs in bursts, low RNA levels can lead to large protein fluctuations^94^, and can be the source of NANOG, and possibly of other factors, cell-to-cell variability. These fluctuations, combined with random co-expression, could also explain the observed asynchronous differentiation of Epi and then PrE cells^1,5,17,35^. Earlier cell heterogeneities have been observed at the 2C and 4C stages^30,47,95,96^, and the question is whether this could be transmitted along their different lineages. Indeed, *Carm1*, which shows cell-to-cell variability at the 4C stage, can enhance *Nanog*, *Sox2* and *Sox21* expression. However, we found that their expression is not coordinated at 16C stage, indicating that the Epi state is not directly inherited from the 4C stage. Therefore if these early 4C heterogeneities influence some genes expression level at 16C stage, such activities would have to impact each Epi gene differently. Still, changes in the levels of a key Epi factor at 16C stage could shift the probability of this key factor to be co-expressed with other Epi factors in individual cells. Thus the number of Epi cells could be modulated by upstream pathways/factors. However in that case, the mechanism of Epi specification would still depend on the occurrence of Epi genes co-expression in the same cell. Thus despite possible deterministic components, the mechanism remains stochastic.

In conclusion, our results reveal that the coordination of Epi/pluripotency gene expression defines the onset of Epi differentiation and that this feature is conserved between mouse and human embryos. The analysis of *DKO* mouse embryos demonstrates that NANOG is central in this process. The Epi cell state stems from a previously uncharacterized cell heterogeneity, which displays random cell-to-cell variability of pluripotency markers. Noteworthy, during reprogramming, iPSC also go through a stochastic phase of cell specification, followed by a deterministic differentiation phase^97^, which is reminiscent of the in vivo Epi differentiation. Altogether, our work brings mechanistic cues to understand how Epi, and thus pluripotency, is established.

## Methods

### Embryo collection and staging

Experiments were performed in accordance with the French and EU guidelines for the care and use of laboratory animals (Ethical Committee C2EA-02, file #26479–2020100111292271). Animals were housed in a pathogen-free facility under a 12-hour light cycle at 30–70% humidity and temperature of 20–26 degrees celsius, with access to standard chow and water. All embryos used in this study were produced by natural mating.

Embryos were collected at the 8-cell (8C), 16C, 32C, 64C, and 90C stage (corresponding to 2.5, 3.0, 3.25, 3.5, and 3.75 days of embryonic development respectively). *WT* embryos from the CD1 strain (*WT-*Ref) (Charles River or Janvier Labs) were staged by total cell count in littermates. Mutants were backcrossed in the CD1 background for more than 9 crosses.

### Genotyping

Single and double mutant embryos were obtained from heterozygous inter-crosses between *Gata6*^*−/+*^ and *Nanog*^−^^*/+*^ mice produced from *Gata6*^*tm2.1Sad*^^18,98^ and *Nanog*^*tm1Yam*^^37^ mice. For a subset of embryo culture and PLA experiments, crosses between the *Gata6*^*tm2.1Sad*^ and the *Zp3-Cre* transgenic lines^99^ were used to increase the number of double mutants. Mendelian ratios were calculated for the different genotypes. As *DKO* and *WT* embryos are rare (1/16 each) and often cannot be found in the same litter, we either processed several litters at the same time, or used *Nanog*^*+/*−^; *Gata6*^*+/+*^ as controls, after checking that removing one *Nanog* allele had no effect on the analysed markers. As slight stage variations can exist within and between litters, embryos were staged according to their total cell number.

Mice and embryos were genotyped as previously described^6,18,100^. Primer pairs for *Gata6* genotyping^98^, using the GoTaq polymerase (Promega) were: 5’-AGTCTCCCTGTCATTCTTCCTGCTC-3’ with 5’-TGATCAAACCTGGGTCTACACTCCTA-3’ for the mutated allele, and 5’-AGTCTCCCTGTCATTCTTCCTGCTC-3’ with 5’–ACGCGAGCTCCAGAAAAAGT–3’ for the wild type allele. Primer pairs for *Nanog* genotyping^37^, using the GoTaq polymerase (Promega) were: 5’-CAGAATGCAGACAGGTCTACAGCCCG-3’ with 5’-AATGGGCTGACCGCTTCCTCGTGCTT-3’ for the mutated allele, or with 5’-GGCCCAGCTGTGTGCACTCAA-3’ for the wild type allele.

For ICM and single-cell analyses, genotyping was carried out using lysed TE cells after immunosurgery^101^. Whole Genome Amplification (WGA) was performed using the REPLI-g kit (Qiagen) followed by PCR genotyping, as above.

### Embryo culture and immunostaining

Embryos were flushed with M2 (Sigma Aldrich) and cultured in KSOM medium (Millipore) for the indicated times. Embryos were incubated with the FGFR inhibitor PD173074 (Sigma Aldrich) at 100 nM, and the MEK1 inhibitor PD0325901 (Sigma Aldrich) at 500 nM^6^. Blastocyst immunostaining was performed as previously described^2^ (see antibodies list, Supplementary Table 5).

Images were captured with the confocal microscopes Leica SPE, SP5 or Sp8 using 40X objectives. Images were analysed with ImageJ (NIH) and Imaris (Bitplane). Cell counts were semiautomated through the Imaris software (Bitplane), as previously described^18^, and DAPI was used to detect cells. In Fig. 2C, only cells with a higher signal in the nucleus compared to the cytoplasm were considered as expressing SOX2 (SOX2+). ICM cell numbers were calculated by subtracting the number of CDX2-positive cells from the total (DAPI-labelled) number of cells. Figures show single z-plane images of cells.

### HCR RNA-FISH

For hybridization chain reaction (HCR) fluorescent in situ hybridization (FISH), the *Fgf4* probe set was designed and purchased from Molecular Instruments. Briefly embryos were fixed in 4% paraformaldehyde (PFA (Electron Microscopy Science)) in 1×PBS for 10 min. After washes in PBT (PBS, 0.1% Tween 20 (Sigma Aldrich)), they were dehydrated in graded series of EtOH/PBT. After graded rehydration, embryos were postfixed in 4% PFA. Prehybridization, hybridization (16 h at 37 °C), amplification (16 h) were carried out according to Molecular Instruments’ protocol. Immunostaining with beta-Catenin was performed subsequently, to allow cell segmentation.

Images were scanned with a LEICA SP8 microscope (40X objective) with z step increments of 0,35 μm. Laser compensation was used for the beta-Catenin channel.

Images were analysed with Imaris and Fiji (through Imaris XT). Cells were segmented with MorpholibJ watershed^102^ in Fiji, using beta-Catenin staining. Then, once the segmented cells were imported as a new channel into Imaris, the number of HCR-FISH spots per cell was calculated using Surfaces or Spots IMARIS functions.

*DKO* and control embryos were processed and imaged at the same time. As *WT* embryos are rare, especially in the same litter as *DKOs*, we used *Nanog*^*+/+*^;*Gata6*^*+/*−^ as control embryos. In *Gata6*^*+/*−^ the number of Epi cells is higher compared to WT’s, however it was shown that NANOG levels in individual cells are the same between WT and *Gata6*^*+/*−^ embryos^38^. Accordingly, the maximal number of *Fgf4* HCR-FISH spots per cell is equivalent in both WT and *Nanog*^*+/+*^;*Gata6*^*+/*−^ (*p* value = 0.1836, Fligner-Killeen test for homogeneity of variance), validating both genotypes as controls for quantification in individual cells.

### Single-cell analyses in the mouse

#### Single cell isolation and RT-qPCR/Fluidigm analysis

Isolated ICM cells (after immunosurgery) and morulae (16C) were incubated in 1X TrypLE™ Express Enzyme (Gibco) at 37 °C for 10 min, and cells were isolated by repeated mouth pipetting using pulled capillaries with serially smaller diameter openings. Each single cell was collected in 5 µl of 2X Reaction Mix (Invitrogen, CellsDirect One-Step qRT-PCR Kit) and stored at −80 °C or processed immediately.

cDNA from single cells with the desired genotypes was pre-amplified with 18 cycles. The quality and genotype (double-check after TE genotyping) of each single-cell sample were checked by qPCR and housekeeping gene primers (*Rps17* and *Rpl30*) and primers to detect the mutations (Table S4). The analysed cells originated from at least four different embryos in each category.

Single-cell qPCR assays were carried out with the Fluidigm Biomark system (GENTYANE facility) on 48.48 or 96.96 Dynamic Arrays, according to the manufacturer’s instructions. For space constraints on the Fluidigm chips, some genes were not analysed at all stages. Cells with absent or low Ct values for the housekeeping genes *Rps17* and *Rpl30* were removed from the analysis (5%). Ct values were normalized to the mean of the housekeeping genes *Rps17* and *Rpl30* using the 2-ΔCt method. In boxplots, values are relative to the mean of all *WT* 32C cells (*WT*-ref and *WT* from the transgenic background when available) to compare between genotypes and stages. Cells were classified in the *Fgf4*− subpopulation when their CT value was >35, and all the other cells were placed in the *Fgf4*+ subpopulation.

#### PCA analyses

Principal component analyses were performed with the 36 genes (Table S6) that were analysed in each sample (16C to 90C *WT-ref*, *WT, Nanog*^*−/−*^*, Gata6*^*−/−*^ and *DKO*), using the R package “pcaMethods” and the “bpca” method^103^ to compute component scores from log2 expression values from stages and genotypes. Scatter plots showing the distribution of cells and genes in the two main components (PC1 and PC2) were produced.

To analyse clustering between *Fgf4*+ and *Fgf4*− cells, first the repeated k-means clustering method was used with *k* = 2 (100 iterations) for each sample (32C, 64C and 90C *WT*-Ref, *WT* and *DKO*). Then, the distribution of *Fgf4*+ and *Fgf4*− cells within the clusters was analysed using the Fisher’s exact test to evaluate the significance of the association between classification types.

#### Single-cell RNA-seq expression correlation analyses

Single-cell expression data were extracted from^34^, taking 40 ICM cells at the 32C stage (early and late) and 33 inner cells at the 16C stage. These cells were already defined and validated by Posfai and collaborators^34^.

All correlation *rho* scores and *p*-values were obtained using the R package “HMisc” and the Spearman method. To generate the correlation heatmap for the 32C stage, first all expression correlations were computed between *Fgf4* and all the genes expressed at the 32C stage. Genes with a *rho* score ≥0.3 (i.e. with a *p* value ≤ 0.05 for 40 samples) were kept for building the matrix.

Correlation scores between these genes were computed and discretized in three individual values: 1=correlated (*rho* score ≥0.3), −1=anticorrelated (*rho* score ≤ −0.3), and 0 = no correlation.

The correlation matrix for the 16C stage was obtained using the 1434 genes selected as correlated or anticorrelated to *Fgf4* expression at the 32C stage. All correlation scores between these genes were computed and correlation was considered significant when the *rho* score ≥0.345 (i.e. *p* ≤ 0.05 for 33 samples). Then, these values were discretized in three individual values: 1=correlated (*rho* score ≥0.345), −1=anticorrelated (*rho* score ≤ −0.345), and 0=no correlation.

Heatmaps were generated using the R package “pheatmap” (Kolde R. 2015; Package ‘pheatmap’: https://CRAN.R-project.org/package=pheatmap). Then, a hierarchical clustering algorithm was applied on both rows and columns to obtain the final heatmap with a dendrogram showing the distances.

For genes expression analyses, cells were considered in the *Fgf4*− subpopulation when the normalized raw reads were equal to 0, and all other cells were placed in the *Fgf4*+ subpopulation.

### Boxplots

The edges of the box represent the 25th and 75th quartiles. The median is represented by the central line. The whiskers extend to 1.5 times the interquartile range (25th to 75th percentile) Cells are plotted individually in single-cell experiments.

### RT-qPCR analysis of single ICM

After immunosurgery^101^, RNA from single ICM was extracted using the PicoPure RNA isolation kit (Arcturus Bioscience). Total RNA from each ICM was reverse transcribed with SuperScript® III Reverse Transcriptase (Invitrogen), 1X PCR buffer2 (Applied Biosystems), 1.33 mM of MgCl_2_ (Applied Biosystems), 2.6U of Superase IN (Ambion), 8U of RNase OUT (Invitrogen), 3.6 µg of Random Primer (Invitrogen), 43.6 µM of dNTP (Invitrogen), and then inactivated at 70 °C for 15 min.

Pre-amplification was performed by PCR (1 cycle at 95 °C for 10 min, and 16 cycles of 15 s at 95 °C and 4 min at 60 °C) with cDNA (1:8 dilution in RNase-free water) and primers mix (Supplementary Table 2) diluted to 5 µM in 25 µl of Taqman preAmp Master mix (Applied Biosystems). Quantitative PCR with 1X SYBR Green I Master (Roche) and 1 µM of primers (Eurogentec) in a final volume of 10 µl was performed on a LightCycler®480 (Roche) with the following program: 95 °C for 5 min, then 40 cycles of 12 s at 95 °C, 12 s at 62 °C and 12 s at 72 °C, and then 1 cycle of 5 s at 95 °C, 1 min at 60 °C. The expression of each transcript was normalized to the mean expression of the housekeeping genes *Rplp0* and *Rps17* using the 2-ΔCt method. Values are relative to the mean at the 32C stage. This method was adapted from described protocols^104,105^.

### Proximity Ligation Assay

PLA was performed using DUOLINK kits (Sigma-Aldrich) following the manufacturer’s instructions. Embryos were fixed in 4% PFA at room temperature for 10 min, and washed twice in PBST (PBS-0.1% Tween 20). Permeabilization was adapted to the different primary antibodies used: 10 min in RIPA solution (150 mM NaCl, 1% (vol/vol) Nonidet-P40, 0.5% (vol/vol) sodium deoxycholate, 0.1% (vol/vol) SDS, 1 mM EDTA and 50 mM Tris-HCl, pH 8.0) and 20 min in PBS-0.5% Triton X-100 for OCT4/SOX2 or NANOG/SOX2 PLA, or 20 min in PBS-0.5% Triton X-100 only for KLF4/SOX2 PLA. Then, blastocysts were blocked in PBST with 10% FBS for 15 min. Embryos were incubated at 4 °C with primary antibodies for PLA (rabbit anti-NANOG, goat anti-SOX2, rabbit anti-OCT4, rabbit anti-KLF4) diluted in the Ab Diluent with the same dilutions used for immunofluorescence, together with an anti-laminB antibody, used for nuclear segmentation, and an anti-NANOG rat antibody (see Table S5 for antibody references). After overnight incubation and washing, embryos were incubated with the secondary antibodies coupled to PLA Probes at 37 °C for 1 h, and washed twice in PBS-0.05% Tween 20. Then, embryos were incubated with 0.025 U/µl ligase at 37 °C for 30 min, washed in PBS-0.05% Tween 20 for 10 min, and incubated with 0.125 U/µl polymerase at 37 °C for 100 min. The reaction was stopped in buffer B for at least 30 min.

Images were scanned with a LEICA SP8 microscope (40X objective) with z step increments of 0,35 μm. Laser compensation was used for the laminB and PLA channel (because the number and not the intensity of dots is analysed), but not for the NANOG channel to allow intensity quantification when necessary.

*DKO* and control embryos were processed and imaged at the same time. As *WT* embryos are as rare as DKO embryos, in some experiments CD1 embryos were included in the experiment and used as controls. We had checked beforehand that there were no difference for each PLA duo among *WT*, *Nanog*^*+/*−^;*Gata6*^*+/+*^, and CD1 embryos.

Images were analysed with Imaris and Fiji (through Imaris XT). Nuclei were segmented with MorpholibJ watershed^102^ in Fiji, using laminB staining. Then, once the segmented nuclei were imported as a new channel into Imaris, the number of PLA spots per nucleus was calculated. Single spots were counted for KLF4/SOX2 PLA, whereas the volume of positive voxels was calculated for OCT4/SOX2 and NANOG/SOX2 PLA because many spots could not be individualized. NANOG relative levels were corrected through the z-stack using the background levels to build a reference line. The mean NANOG intensity in each segmented nucleus was divided by the slope of the reference line. The corrected values for each cell were normalized against the average of the corrected values for all cells. On the violin plot, cells were allocated to the NANOG^high^ or NANOG^low^ populations using the repeated k-means clustering method with *k* = 2 (250 iterations).

### In silico single-cell analysis of human ICM cells

- Single-cell expression data were extracted from^74,76,106^, selecting already identified ICM cells at E6 and E7 and all cells at E5 (*n* = 54 embryos, and *n* = 518 cells in total) (data S8).

- PCA was performed using data on 36 markers for the 518 cells (Table S6) and the “bpca” method in the R package “pcaMethods”^103^ to compute component scores of log2 expression values from the different stages. Scatter plots showing the distribution of cells and of genes in the two main components (PC1 and PC2) were produced.

- k-means clustering with *k* = 4 was applied on the PCA coordinates to segregate clusters #1, 2 and 3. Then, k-means clustering with *k* = 3 was applied on cluster 3 to segregate clusters #3.1, 3.2, and 3.3. Cells from clusters 3.2 and 3.3 belonging to TE (Fig. S12) were discarded. Cells from cluster 3.1 belonging to embryos without TE cells were also discarded, to remove cells from potential earlier embryos that have not completed the ICM/TE differentiation because these cells could be progenitors of both TE and ICM. Finally, 88 cells were identified as ICMp cells, and 258 as ICMd cells (data S8).

- For gene expression analyses, cells were categorized in the *FGF4*- subpopulation when the normalized raw reads were equal to 0, and all other cells were placed in the *FGF4*+ subpopulation.

- Correlation *rho* scores and *p*-values were obtained using the R package “HMisc” and the Spearman method. To obtain correlation heatmaps for ICMd cells, first all expression correlations between *FGF4* and all the genes were computed. Genes with a *rho* score ≥0.3 (*p* ≤ 0.05) were kept for building the matrix.

Correlation scores between these genes were computed and discretized in three individual values: 1=correlated (*rho* score ≥0.3), −1=anticorrelated (*rho* score ≤ −0.3), and 0=no correlation.

The correlation matrix for ICMp cells was obtained by using the genes selected with a correlated or anticorrelated expession with *FGF4* expression in ICMd cells. All correlation scores between these genes were computed and correlation was considered significant if the *rho* score ≥0.3 (*p* ≤ 0.05). Then, these values were discretized in three individual values: 1=correlated (*rho* score ≥0.3), −1=anticorrelated (*rho* score ≤ −0.3), and 0 = no correlation.

Heatmaps were generated with the R package “pheatmap”.

The same method was used for correlations with *NANOG* expression.

**Generation of graphs** (dot plots, boxplots, PCA, Seurat plots)

Comparison gene plots were made using the R package “ggplot2”.

### Statistical analyses

Statistical tests were performed with R. Statistical significance was assessed using the Wilcoxon-Mann-Whitney test (non-parametric) for expression levels (see the different Excel sheets in Supplementary Data 15). Fisher’s exact test was performed to analyse Fgf4+ and Fgf4– cell distributions on PCA maps. The Fligner-Killeen non-parametric test for homogeneity of variance was used to analyse the distribution of *Fgf4*+ cells in *WT* and *DKO* cells. For comparing PLA results in three samples, the Kruskal-Wallis test was used, followed by the Wilcoxon-Mann-Whitney test for two-by-two comparisons.

### Reporting summary

Further information on research design is available in the Nature Research Reporting Summary linked to this article.

## Supplementary information

Supplementary Information

Peer Review File

Description of Additional Supplementary Files

Supplementary Data 1

Supplementary Data 2

Supplementary Data 3

Supplementary Data 4

Supplementary Data 5

Supplementary Data 6

Supplementary Data 7

Supplementary Data 8

Supplementary Data 9

Supplementary Data 10

Supplementary Data 11

Supplementary Data 12

Supplementary Data 13

Supplementary Data 14

Supplementary Data 15

Reporting Summary

## Data Availability

Source data are provided with this paper. Additionnal data that support this study are available from the corresponding author upon reasonable request.

## Source data

Source Data
