# Supplementary material for: NANOG initiates epiblast fate through the coordination of pluripotency genes expression
Source: Nat Commun. 2022 Jun 21;13:3550. doi: 10.1038/s41467-022-30858-8 (PMC9213552; doi:10.1038/s41467-022-30858-8)
Supplement: Supplementary file 1 — Supplementary Information [file 41467_2022_30858_MOESM1_ESM.pdf]

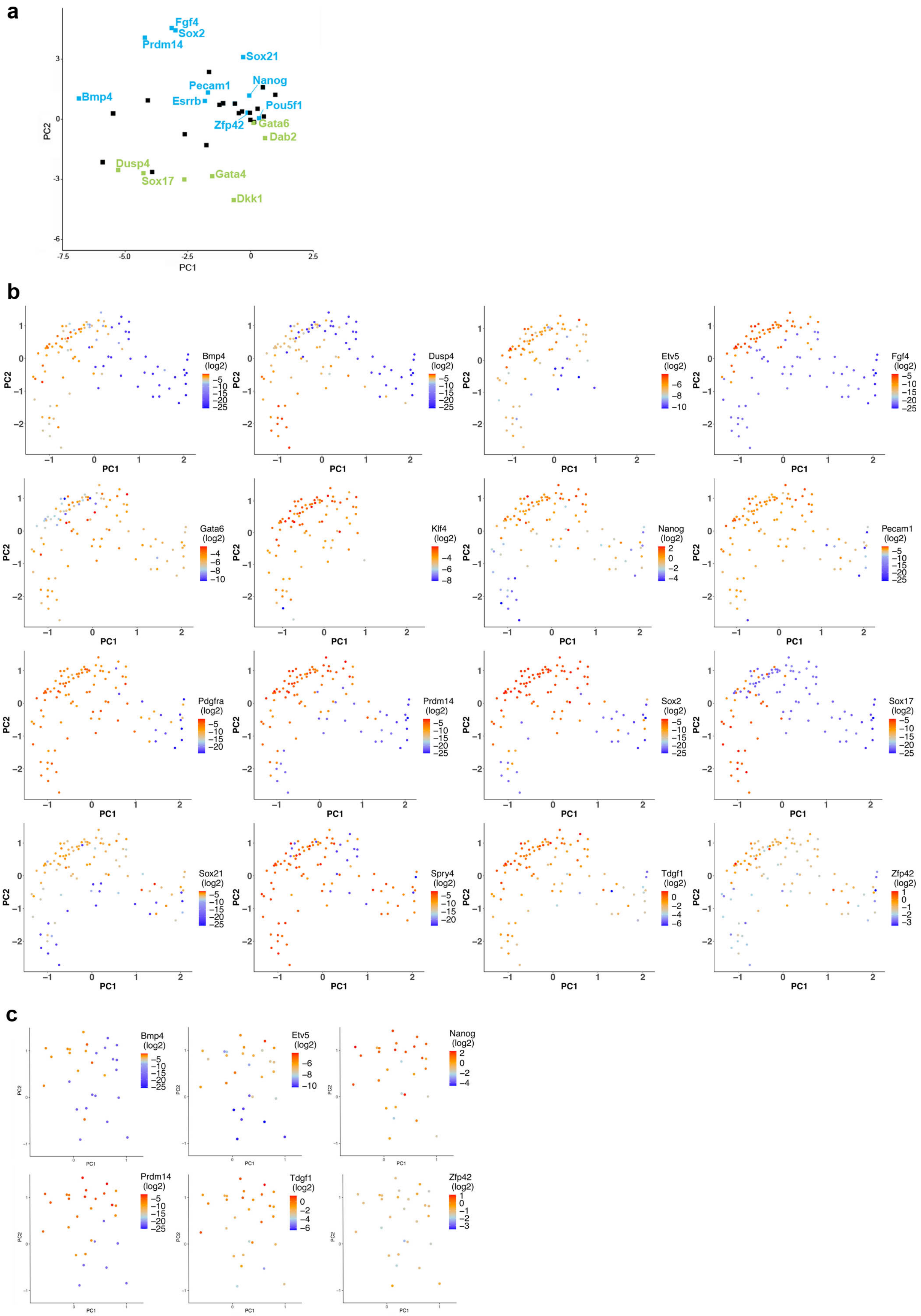

Supplementary Figure 1: a, PCA loading map (see Fig. 1b) showing the distribution of the measured gene expression levels. Epi and PrE genes are labelled in blue and green, respectively; other genes are represented by

black dots. **b**, PCA map shown in (Fig. 1b) with graded colours indicating the expression level of the tested genes in each cell at the four stages. For *Etv5* and *Klf4,* experiments in 16C-stage cells were not carried out. **c**, PCA map showing 32C stage cells only, with expression gradient as in **(a)**. Source data are provided as a Source Data file.

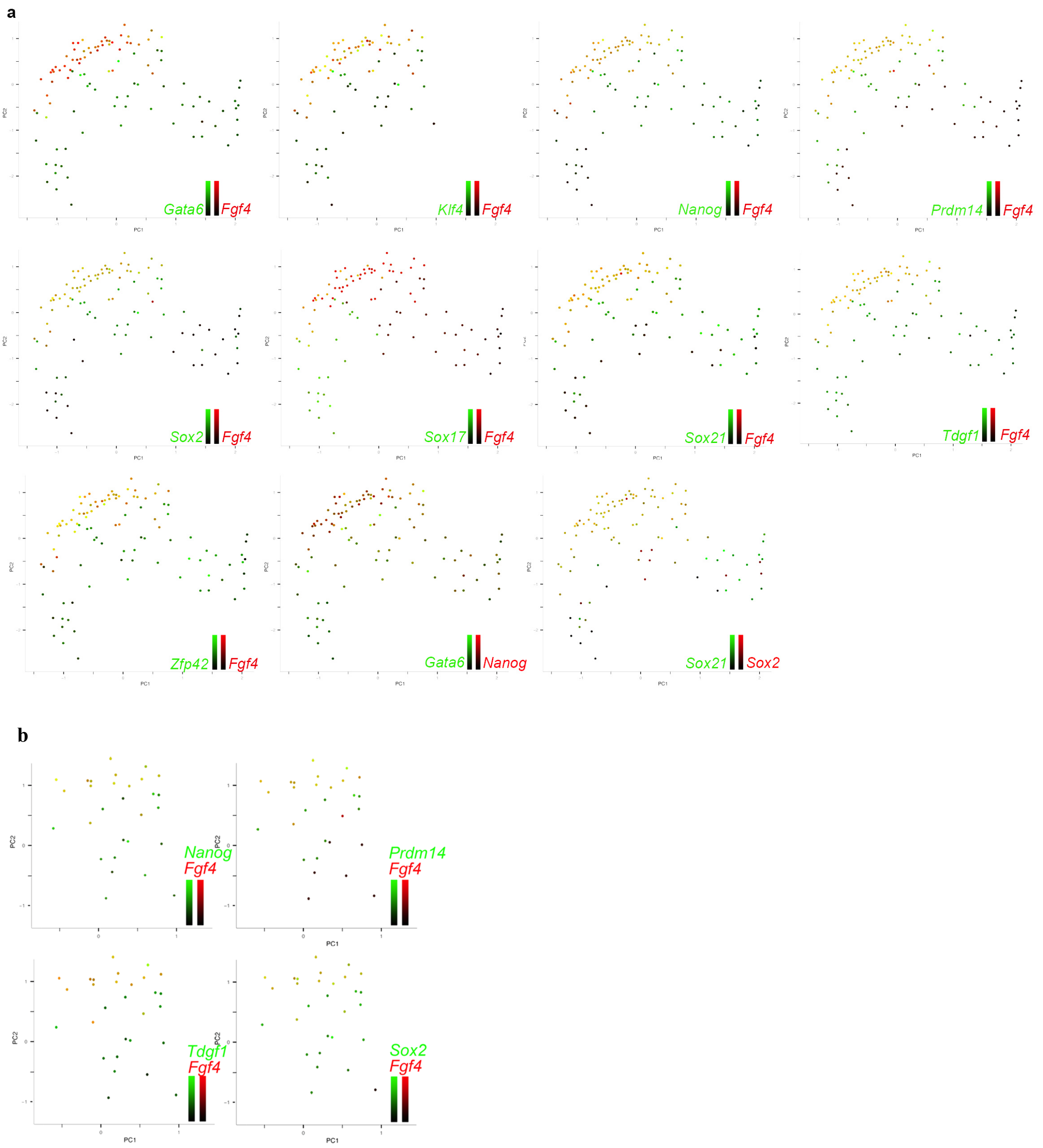

Supplementary Figure 2: Co-expression of genes in individual cells. a, Paired gene expression, in red and green, to show their colocalization (yellow) on the PCA map (see Fig 1b). For each gene, the intensity scale (log2) is shown in Supplementary Figure 1b. **b**, Examples of paired gene expression at the 32C stage only, as in **(a)**. Source data are provided as a Source Data file.

	32C	
Fgf4 vs	*Rho*	*p*-value
Nanog	0.64	0.0002
Klf2	0.59	0.0008
Prdm14	0.54	0.003
Tdgf1	0.51	0.005
Sox2	0.49	0.008
Pecam1	0.46	0.013
Klf4	0.45	0.015
Sox21	0.40	0.034
Fgfr2	-0.41	0.028

Supplementary table 1: Positive and negative correlations of expression (Spearman test) for *Fgf4* with genes expressed in 32C stage cells. Source data are provided as a Source Data file.

	32C		16C	
Nanog vs	*Rho*	*p*-value	*Rho*	*p*-value
Klf4	0.78	0.000002	n.d	n.d
Klf2	0.69	0.00005	n.d	n.d
Tdgf1	0.69	0.00006	-0.17	0.51
Sox2	0.69	0.00006	0.16	0.387
Fgf4	0.64	0.0003	n.a.	n.a.
Pou5f1	0.62	0.0004	0.52	0.028
Pecam1	0.62	0.0004	0.27	0.282
Prdm14	0.59	0.0008	0.30	0.219
Sox21	0.59	0.0009	-0.31	0.209
Etv5	0.52	0.003	n.d.	n.d.
Fgfr2	-0.45	0.01481	n.a.	n.a.

	32C		16C	
Sox2 vs	*Rho*	*p*-value	*Rho*	*p*-value
Nanog	0.69	6.05 E-05	0.16	0.387
Klf4	0.69	3.69 E-05	n.d	n.d
Sox21	0.64	0.0002	0.13	0.483
Klf2	0.61	0.0004	n.d	n.d
Pecam1	0.60	0.0007	0.18	0.330
Pou5f1	0.58	0.0013	0.52	0.003
Tdgf1	0.52	0.0045	0.23	0.219
Fgfr2	-0.58	0.0012	n.a.	n.a.

Supplementary table 2: Positive and negative correlations (Spearman test) of *Nanog* and *Sox2* expression with that of Epi genes, at 32C and 16C stage cells (significant values are highlighted in grey). For space constraints on the Fluidigm chip some genes were not analysed at the 16C stage (n.d.). Other genes are not expressed at the 16C stage, thus the correlation analysis could not be carried out (n.a.). Source data are provided as a Source Data file.

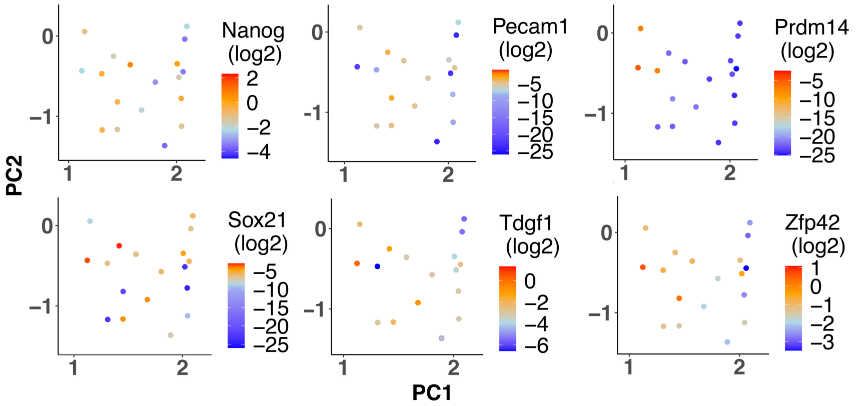

Supplementary Figure 3: Relative gene expression in individual cells at the 16C stage. PCA map (see Fig 1b) showing only the 16C stage, with gene expression levels shown as a gradient. *Fgf4* is not detected at this stage. Source data are provided as a Source Data file.

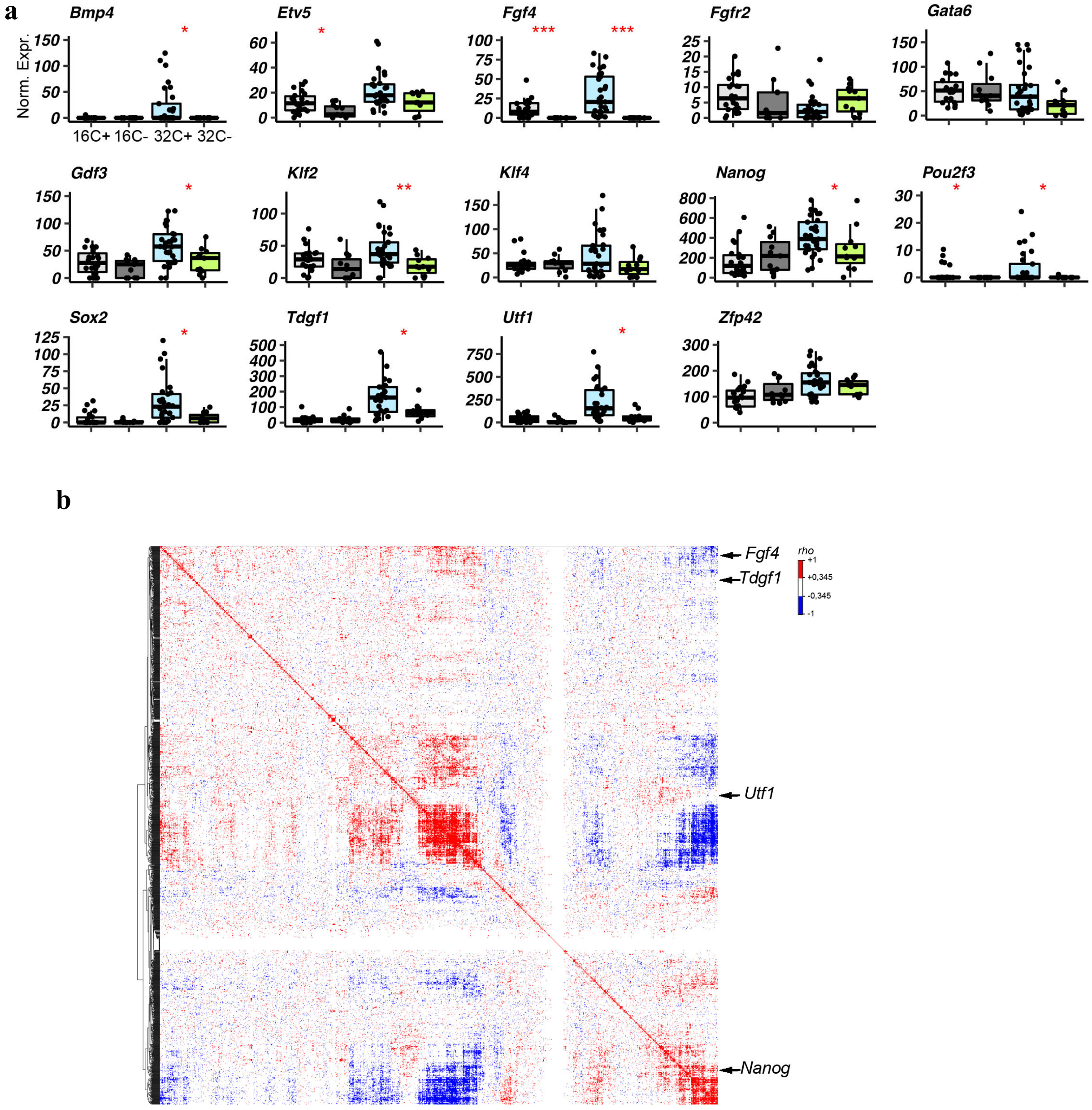

Supplementary Figure 4: RNA-seq gene expression analysis in 16C and 32C-stage mouse ICMs. **a,** Normalized gene expression in 16C and 32C cells categorized as *Fgf4*+ (+) (22 cells at 16C and 29 at 32C) and *Fgf4*- (-) (11 cells at 16C and 32C); *p<0.05, **p<0.01 ,***p<0.001 (Two-sided Wilcoxon test between *Fgf4*+ and *Fgf4*- cells, see Supplementary Data 15 for exact pvalues). The edges of the box represent the 25th and 75th quartiles. The median is represented by the central line. The whiskers extend to 1.5 times the interquartile range (25th to 75th percentile). Cells are plotted individually. **b,** Spearman correlation matrix for gene expression analysis using RNA-seq data at 16C stage. The 1434 genes selected by their correlated expression with *Fgf4* at the 32C stage (Fig 1f; Supplementary Data 3) were analysed at in 16C inner cells. Expression correlation heatmaps were built with all selected gene pairs, with 33 inner cells at the 16C stage (see Supplementary data 5 for detailed maps on vector-based PDFs). Genes are ordered in a hierarchical tree for similarity. Spearman correlation tests (p < 0.05; for 33 samples: Rho> 0.345 or < -0.345).

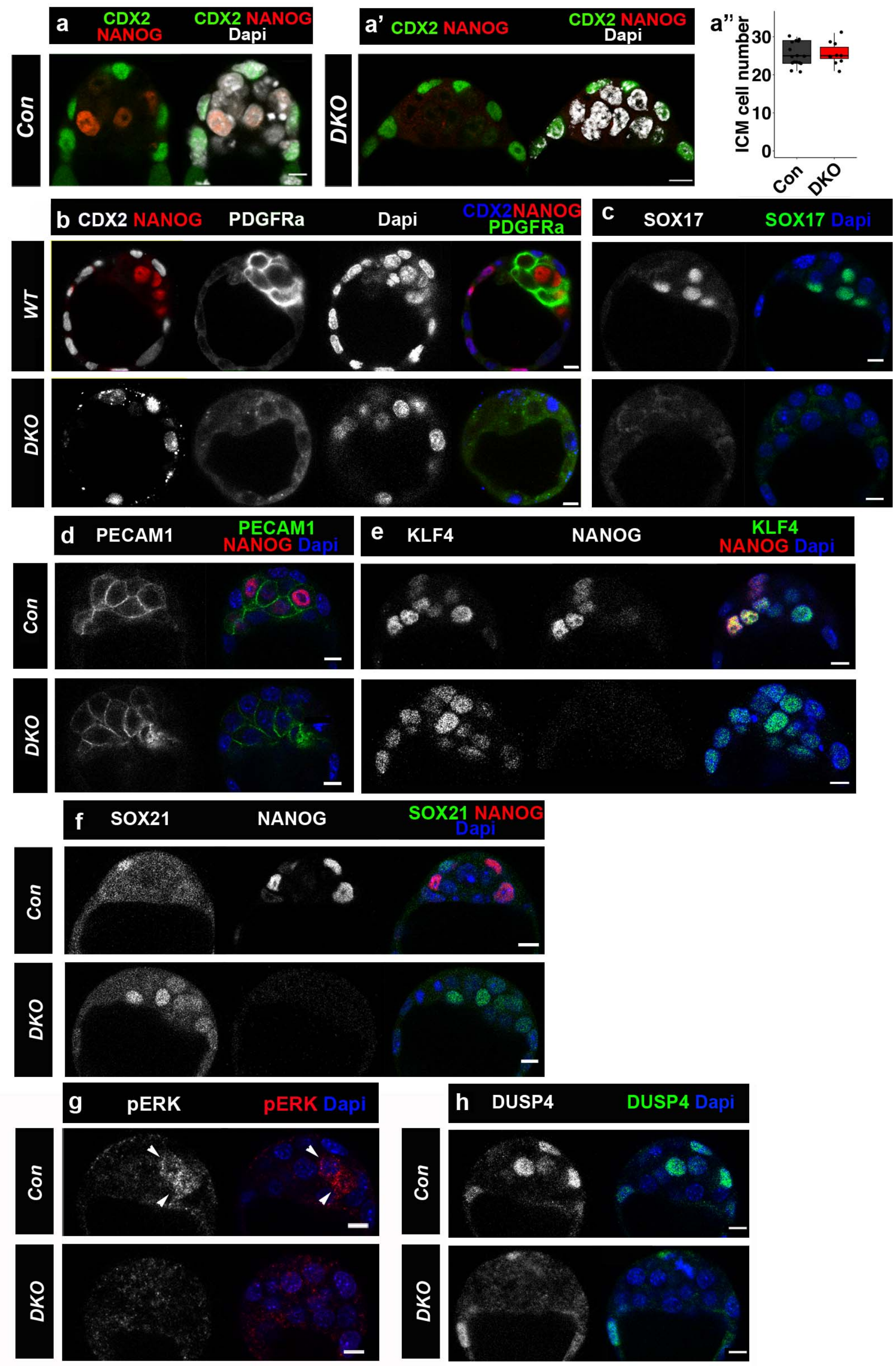

Supplementary Figure 5: **Localization of different markers in *DKO* blastocysts by IF analysis**. CDX2 and NANOG expression at the 90C stage in control (**a**) and DKO (**a'**) embryos. **a'',** ICM cell numbers in 64-90C control (n=16) and *DKO* (n=10) embryos (Two-sided Wilcoxon test p=0.75) from IF analyses. The edges of the box represent the 25th and 75th quartiles. The median is represented by the central line. The whiskers extend to 1.5 times the interquartile range (25th to 75th percentile). Cells are plotted individually. **b**, CDX2, PDGFRa and NANOG expression at the 90C stage. **c**, SOX17 expression at the 64-90C stages. **d, e, f,** PECAM1, KLF4 and SOX21 are expressed in both control and *DKO* embryos at the 64-90C stages. Note that SOX21 expression is stronger in *DKO* ICM cells because its expression decreases at around 64-90C in *WT* ICMs. **g**, Representative immunofluorescence images of pERK in 64-90C *DKO* embryos. **h**, At the 64-90C stages, DUSP4 can be detected in some TE cells, but not ICM cells of *DKO* embryos. Number of analysed *DKO* embryos at 64-90C (fully penetrant phenotypes): CDX2 n>8; SOX17 n=4; PDGFRa n=5; KLF4 n=4; SOX21 n=5 (3 at 64-90C; 2 at 32C); PECAM1 n=5 (3 at 64-90C and 2 at 32C stages); pERK n=5 and DUSP4 n=5. Scale bars: 10 µm.

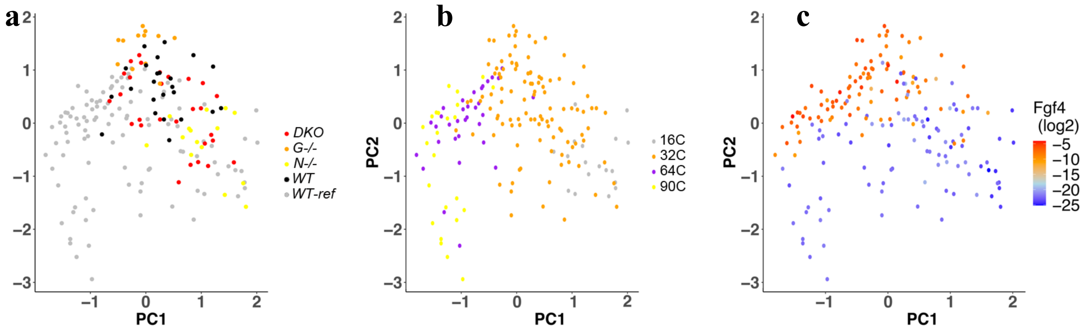

Supplementary Figure 6: a, PCA map built with the cells of the four genotypes (*WT, Nanog*$^{-/-}$, *Gata6*$^{-/-}$ and *DKO*) as well as with *WT-Ref* cells, used as reference. Scores: PC1: 19.38% PC2: 13.13%. **b**, Same PCA map with developmental stages. **C**, Same PCA map with *Fgf4* expression intensities. Source data are provided as a Source Data file.

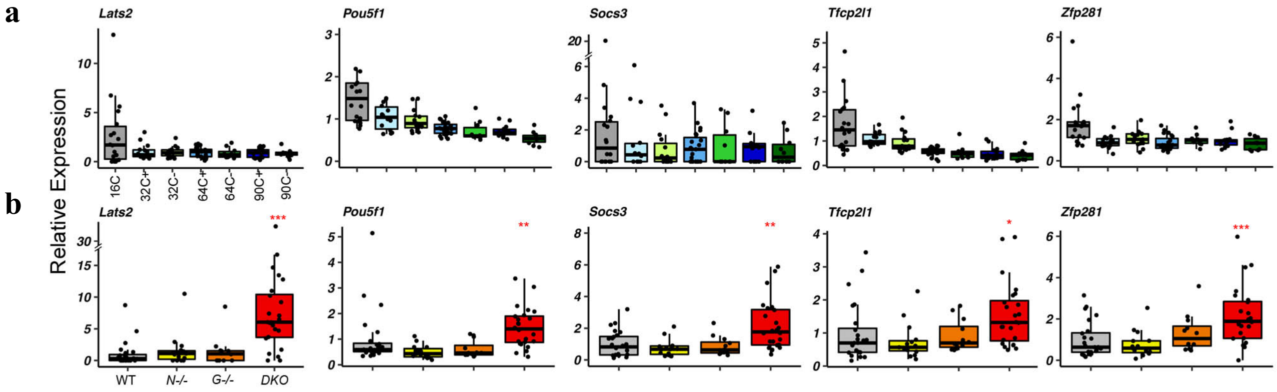

Supplementary Figure 7: a, Expression of markers preferentially expressed at higher levels at 16C stage by RT-qPCR. **b**, High expression of these markers is maintained in *DKO* ICMs at the 32C stage, while is reduced in *WT* embryos; **p*<0.05, ***p*<0.01(Two-sided Wilcoxon test compared with *WT*, see Supplementary Data 15 for exact pvalues). Cell numbers provided in Fig 1a and 3a. The edges of the box represent the 25th and 75th quartiles. The median is represented by the central line. The whiskers extend to 1.5 times the interquartile range (25th to 75th percentile). Cells are plotted individually in single-cell experiments. Source data are provided as a Source Data file. Source data are provided as a Source Data file.

	WT		*DKO*	
	Rho	*p*-value	*Rho*	*p*-value
Sox2 vs Sox21	0.71	0.0001	0.78	1.31E-05
Sox2 vs Tdgf1	0.54	0.007	0.23	0.272
Nanog vs Sox2	0.54	0.007	0.17	0.411
Sox2 vs Pou5f1	0.53	0.009	0.20	0.358
Sox2 vs Pecam1	0.51	0.012	0.53	0.007
Fgf4 vs Tdgf1	0.49	0.016	0.066	0.759
Nanog vs Pecam1	0.49	0.016	0.30	0.151
Nanog vs Sox21	0.48	0.018	0.21	0.326
Nanog vs Fgf4	0.43	0.035	0.04	0.865
Fgf4 vs Nanog	0.43	0.036	0.036	0.866
Sox2 vs Zfp42	0.42	0.044	0.4	0.054
Nanog vs Tdgf1	0.41	0.045	0.34	0.107
Fgf4 vs Zfp42	0.40	0.055	-0.019	0.93
Fgf4 vs Pecam1	0.39	0.056	0.341	0.103

Supplementary Table 3: Correlation of the expression (Spearman test) of different Epi markers in *WT* or *DKO* ICM cells. Significant correlations are highlighted in grey (*p*-value <0.05). Source data are provided as a Source Data file.

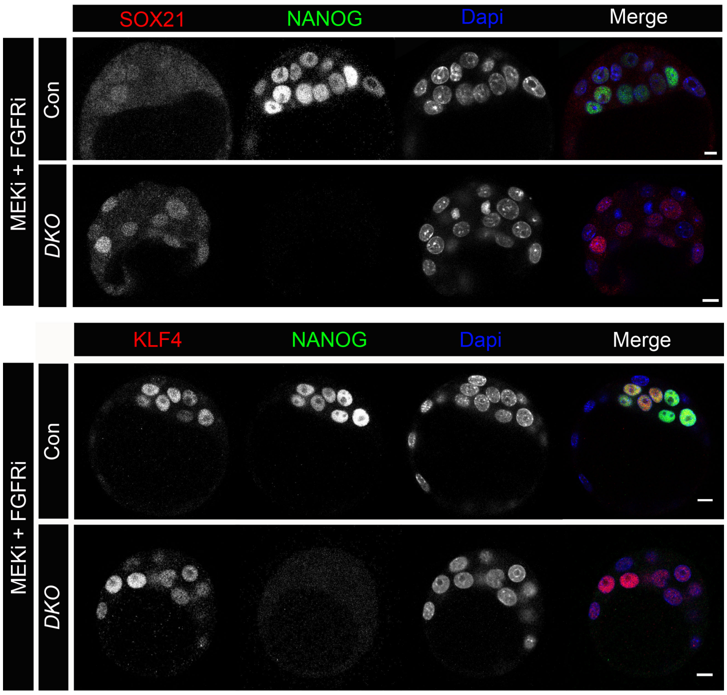

Supplementary Figure 8: *DKO* and control embryos cultured with FGFR and MEK1 inhibitors from the 8C to the 64-90C stage. SOX21 and KLF4 remain expressed heterogeneously in the absence of *Nanog*, *Gata6* and also of FGF pathway activity. Number of analysed *DKO* embryos: SOX21 (n=6); KLF4 (n=5). Scale bars: 10 µm.

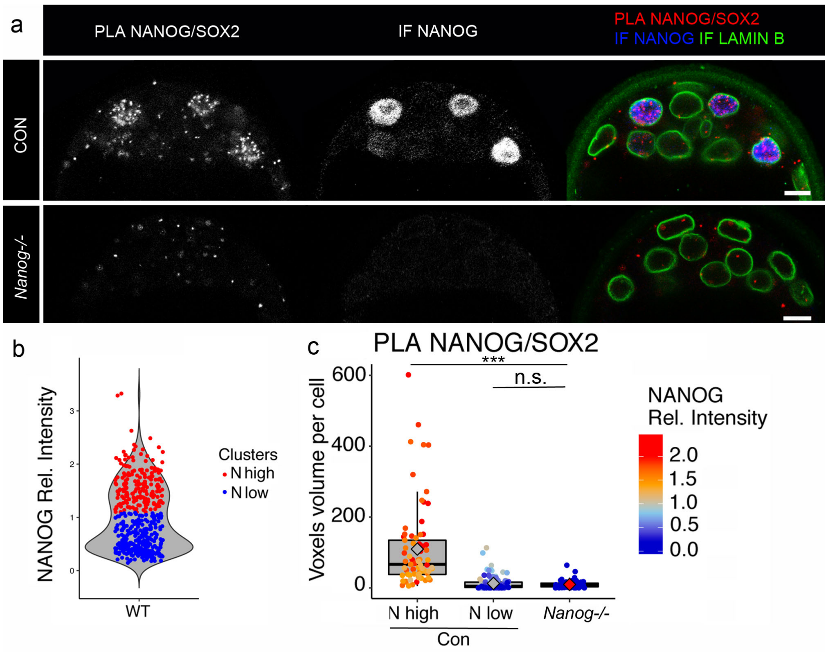

Supplementary Figure 9: a, NANOG-SOX2 PLA with NANOG and LaminB immunofluorescence in control and *Nanog*$^{-/-}$ (n=6) ICMs. Scale bars: 10 µm. **b**, Violin plot showing the distribution of ICM cells according to their NANOG level in all *WT* embryos examined by PLA (n= 22). Clusters of NANOG-high (n=73 cells) and NANOG-low (n=102 cells) cells were determined by K-means clustering. **c**, Quantification of PLA signals in control (NANOG-high and NANOG-low) and *Nanog*$^{-/-}$ embryos (n=62 cells). Two-sided Kruskal-Wallis test for the three cell types ($p < 2.2e\text{-}16$) was followed by pairwise Wilcoxon comparisons (***p<2e-16; n.s., p =0,43). The edges of the box represent the 25th and 75th quartiles. The median is represented by the central line. The whiskers extend to 1.5 times the interquartile range (25th to 75th percentile). Cells are plotted individually. Source data are provided as a Source Data file.

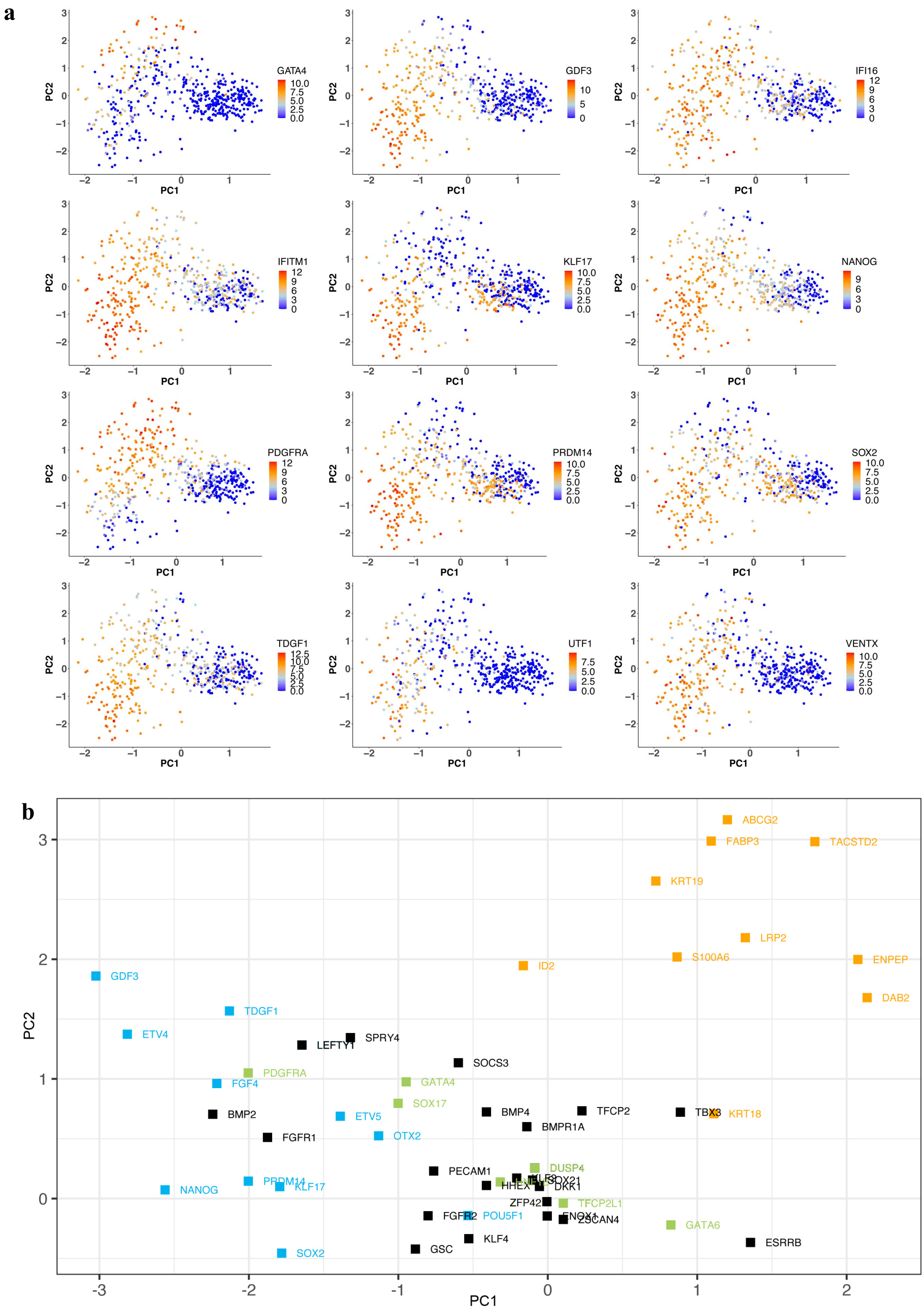

Supplementary Figure 10: Gene expression in human cells. a, PCA map shown in Fig. 4 with graded colours indicating the expression level (log2) of the indicated genes. **b,** PCA loading map showing the distribution of the measured gene expression levels. Known[74] human Epi markers are labelled in blue, PrE markers in green and TE markers in yellow; other genes are represented by black dots.

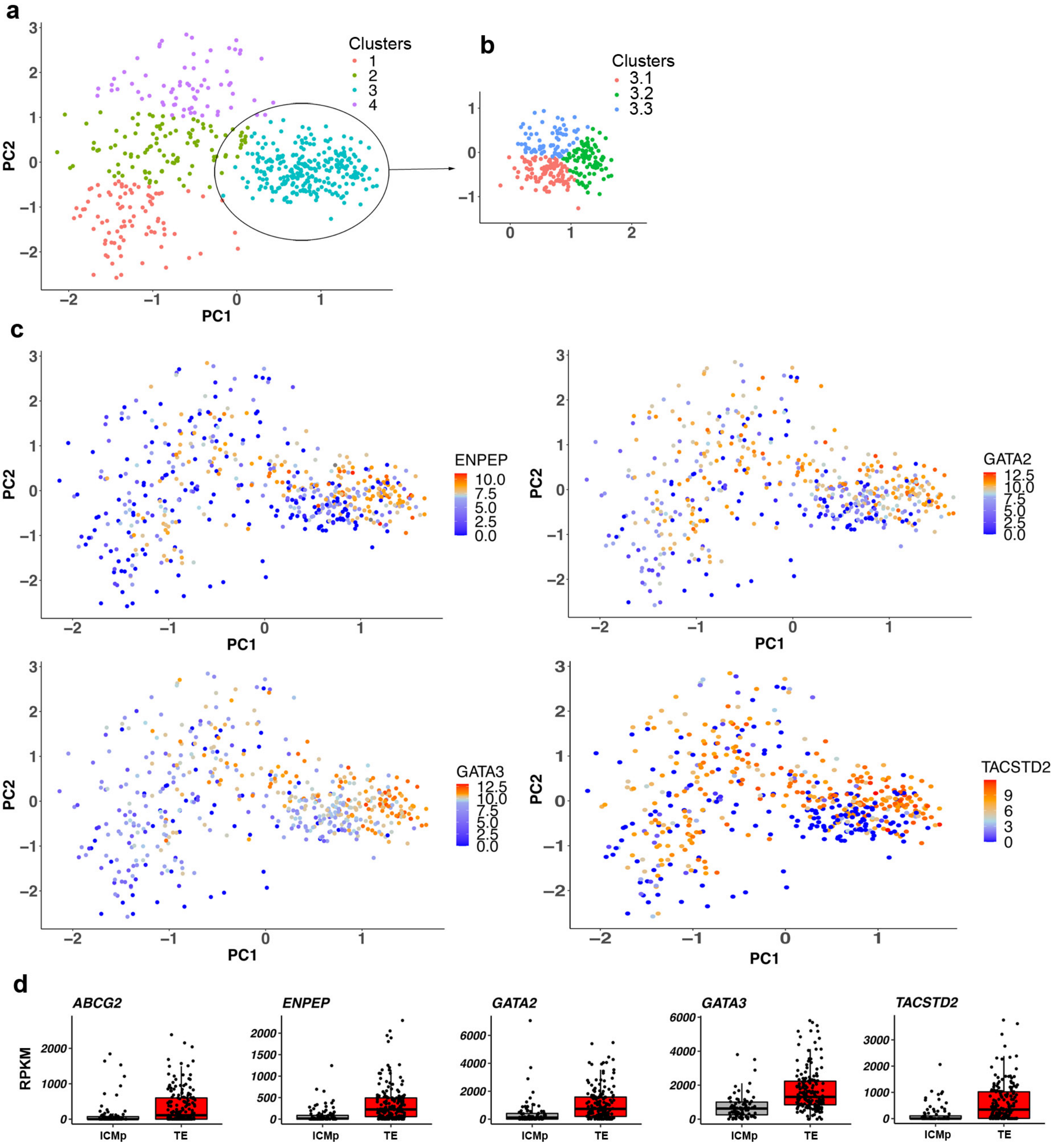

Supplementary Figure 11: Selection of ICMp cells. a, K-means clustering of four groups on the PCA map of Fig 4a-c. Cluster 3 comprises cells that do not express *FGF4* or *SOX17*, and thus belong to ICM-progenitor (ICMp) or early TE populations. **b,** Cluster 3 in **(a)** was subdivided in three K-means clusters. **c,** Normalized expression of ENPEP, GATA2, GATA3 and TACSTD2 (TE markers) on the PCA map. Their different expression levels segregate cluster 3 into two populations. This identifies the ICMp cell population, confirmed by K-means clustering (cluster 3.1 in Fig **b,** n=88 cells, see list in Supplementary data 8). **d**, TE marker expression in ICMp cells and E5 TE cells (clusters 3.2 and 3.3 in (**b**), n=171 cells). Two-sided Wilcoxon test between TE and ICMp cells (p-values: *ABCG2*<2.00 e^{-06} ; *ENPEP*=7.10 e^{-13} ; *GATA2*= 1.20 e^{-10}; *GATA3*=6.70 e^{-14} ; *TACSTD2*= 5.90 e^{-12}). The edges of the box represent the 25th and 75th quartiles. The median is represented by the central line. The whiskers extend to 1.5 times the interquartile range (25th to 75th percentile). Cells are plotted individually.

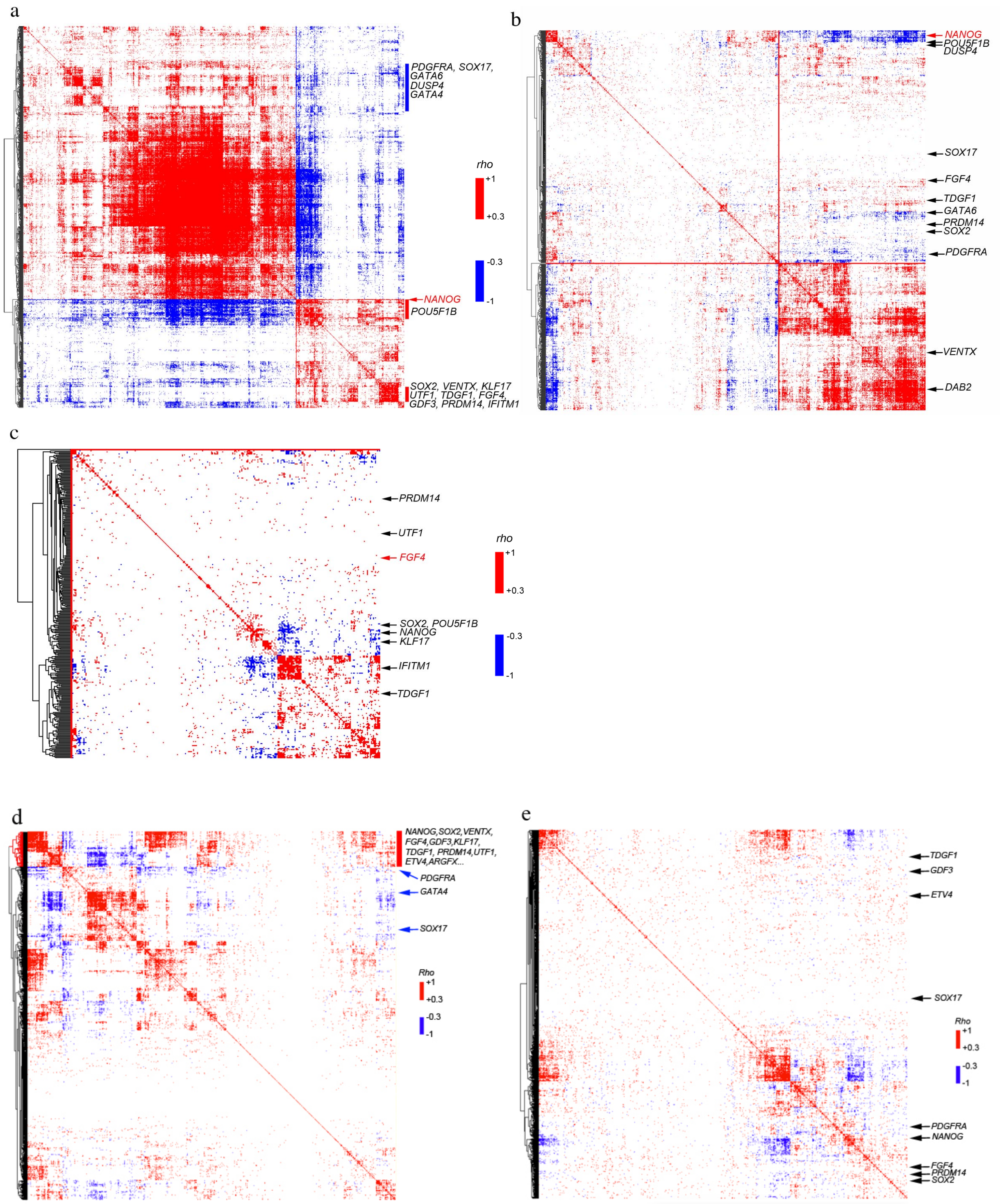

Supplementary Figure 12: Spearman correlation matrices for gene expression in human ICM cells. a, Spearman correlation matrix for the paired expression of 1052 genes (selected by their correlation with *NANOG* expression, see Supplementary data 3) in ICMd cells and **b,** in ICMp cells (*rho>0.3* or <*-0.3*). **c**, Spearman correlation matrix for the paired expression of the 308 genes from Fig 2a in ICMp cells (*rho>0.3* or <*-0.3*). **d, e**, Spearman correlation matrix for the paired expression of 1065 genes selected from Striparo et al. (see Supplementary data 3) in ICMd (**d**) and ICMp (**e**) (*rho>0.3* or <*-0.3*). Genes are ordered in hierarchical trees for similarity (see Supplementary data 10,11,12,13,14 for detailed maps on vector-based PDFs).

Supplementary Table 4: List of the primers used.

RefSeq Accession #	Gene Symbol	Forward 5'	Reverse 3'
NM_007395.3	***Acvr1b***	AGAAGCTACGGCCCAATGTC	TCACATCTTCCTGCACGCTT
NM_015732	***Axin2***	CTCCCCACCTTGAATGAAGA	GATTGACTGGGTCGCTTCTC
SA_βGeo	***βGeo*****	ACTATCCCGACCGCCTTACT	TAGCGGCTGATGTTGAACTG
NM_007554.2	***Bmp4***	TGAGCCTTTCCAGCAAGTTT	CTTCCCGGTCTCAGGTATCA
NM_009758.4	***Bmpr1a***	CAGACGGTGTTAATGCGTCA	TGGCACATTTCAGGAAGTCA
NM_007561	***Bmpr2***	TGAGGAGAGGATGGCTGAAC	GATTTTTGGCACACGCCTAT
NM_007673	***Cdx2***	TCTCCGAGAGGCAGGTTAAA	GCAAGGAGGTCACAGGACTC
NM_009931	***Col4a1***	CCATCTCTGGGGACAACATC	GAAGGAATAGCCGATCCACA
NM_001033638	***Crxos1***	CCTCCAGGATCGAAAGATCA	GCATCTCCTCCTCCAAGGAT
NM_001310693	***Csde1***	TCCTTTGGAACTTGTGCTGA	CTTGCCGTTCTGAACACTGA
NM_001037905	***Dab2***	AGTCGTCGATCTTAAAGACCTTT	CTCTTCTGCTTTGTTGGCTTCT
NM_010051	***Dkk1***	TCTCTATGAGGGCGGGAACA	TTTCGGCAAGCCAGACAGAT
NM_176933.4	***Dusp4***	AGCTCCTGGTTCATGGAAGC	ACTCAAAAGCCTCCTCCAGC
NM_026268	***Dusp6***	CAGCGACTGGAATGAGAACA	CGGCCTGGAACTTACTGAAG
NM_172813	***Enox1***	CCATCCGAAAGAGCAAGAAG	CGACCCTAATCGCATTCTGT
NM_007936	***Epha4***	ACGACACTAGAAGCCGTGGT	CTGGCTCAGACAGGAACCAT
NM_001159500	***Esrrb***	TAGGGGTTGAGCAGGACAA	CTACCAGGCGAGAGTGTTCC
NM_008815	***Etv4***	GCAGGGAAAGCTCATGGAC	GAGCCACGTCTCTTGGAAGT
NM_023794	***Etv5***	CAGAACCTGGATCACAGCAA	GACTGAGGAGGGAAGGGATG
NM_008007	***Fgf3***	GGAGATTACTGCGGTGGAAG	GGCGGGAAGCATATGTATTG
NM_010202	***Fgf4***	ACTACCTGCTGGGCCTCAA	ACTCCGAAGATGCTCACCAC
NM_001079908	***Fgfr1***	GCTATAACCCCAGCCACAAC	AGCCAAAGTCTGCGATCTTC
NM_010207	***Fgfr2***	CACCAACTGCACCAATGAAC	GAATCGTCCCCTGAAGAACA
NM_008010	***Fgfr3***	GGGGTGGTCTTCTTCCTCTT	GACAAGGGGTGTGTTGGAGT
NM_008011	***Fgfr4***	CACATGCCTTGTGGAGAACT	GTCGCTGTACACCTTGCAGA
NM_010446	***Foxa2***	CATCCGACTGGAGCAGCTA	TGTGTTCATGCCATTCATCC
NM_008239	***Foxq1***	ATTGGAGGTGTTCGTCCCAC	CTAAGGAGTCGTCACCAGCC
NM_008091	***Gata3***	GATGTAAGTCGAGGCCCAAG	GCAGGCATTGCAAAGGTAGT
NM_008092.3	***Gata4***	GAGATGGGACGGGACACTAC	TAGTCTGGCAGTTGGCACAG
NM_010258	***Gata6ex4-5****	GCCAACTGTCACACCACAAC	GGTTTTCGTTTCCTGGTTTG
NM_010258	***Gata6ex1****	ATTCACCAGCAGCGACTAGC	AGTCAAGGCCATCCACTGTC
NM_010258	***Gata6Δ2*****	ATTCACCAGCAGCGACTAGC	GACAGGTCCTCCAACAGGTC
NM_010262	***Gbx2***	AGACGGCAAAGCCTTCTTG	GGGTCATCTTCCACCTTTGA
NM_008108	***Gdf3***	AACCTGCTTCAGCTTCTCCC	TCTAGAGTCAGCTGGGCCAT
NM_008110	***Gdf9***	CAAACCCAGCAGAAGTCACC	TAGCAAGACCGATTTGAGCA
NM_010351	***Gsc***	GAAGCCCTGGAGAACCTCTT	CTTCTCGGCGTTTTCTGACT
NM_008245	***Hhex***	ACTACACGCACGCCCTACTC	ACTTGACCGCCTTTCCTTTT
NM_009330	***Hnf1b***	CTCCTCTCCACCCAACAAGA	GCTGGGGAGACTTGCTGTAA
NM_001312907	***Hnf4a***	GGTGCCAACCTCAATTCATC	CCACACATTGTCGGCTAAAC
NM_010496.3	***Id2***	GGTGGACGACCCGATGAGT	TGCCTGCAAGGACAGGATG
NM_008452	***Klf2***	CCAAGAGCTCGCACCTAAAG	GTGGCACTGAAAGGGTCTGT
NM_010637	***Klf4***	CCAAAGAGGGGAAGAAGGTC	CGTCCCAGTCACAGTGGTAA
NM_009769	***Klf5***	CTCCGGAGACGATCTGAAAC	GAACTGGAGGGAGCTGAGG
NM_008480	***Lama1***	CACATCAGCGCCAATGCTAC	TTGTTGGTGCCATCGATTGC
NM_010690	***Lats1***	TCATGGAGCAGCACGTAGAG	CATTTGATCCTGGGCATCTT
NM_015771	***Lats2***	GCAACACGTGGAGAATGTCA	CTTCCTCATCTGCTCCTGCT
NM_010094	***Lefty1***	CGCTGGACCTCAAGGACTAT	GGCGGTTCTAGGATCCAGTT
NM_028016	***NanogΔ2*****	ATGCCTGCAGTTTTTCATCC	GGCAGGTCTTCAGAGGAAGG
NM_028016	***Nanog***	AAGTACCTCAGCCTCCAGCA	GCTTGCACTTCATCCTTTGG
NM_013611	***Nodal***	CTCTGGCGTACATGTTGAGC	CGCCCATACCAGATCCTCT
NM_010929.2	***Notch4***	CAGGAGTGTGAATCGGAGGT	GGGTTCCAGATTTCCTAGCC
NM_007430	***Nr0b1***	TCCAGGCCATCAAGAGTTTC	ATCTGCTGGGTTCTCCACTG

RefSeq Accession	Gene Symbol	Forward 5’	Reverse 3’
NM_144841	***Otx2***	CTCGACGTTCTGGAAGCTCT	CTGACCTCCATTCTGCTGCT
NM008808.3	***Pdgfα***	CAACCTGAACCCAGACCATC	TTGGCAATGAAGCACCATAC
NM_011058	***Pdgfrα***	GACCTGCAGTGGACTTACCC	CCAGTTTGATGGATGGGAGT
NM_008816	***Pecam1***	ACGCTGGTGCTCTATGCAAG	TCAGTTGCTGCCCATTCATCA
NM_013633	***Pou5f1***	AGCCGACAACAATGAGAACC	TGATTGGCGATGTGAGTGAT
NM_001081209	***Prdm14***	GAGCACCCAACCGACTTACA	GCTGGAGGGAGTCTTTGTCC
NM_023894	***Rhox9***	GGATGGGTGTGGATGAATCT	TCATCAGCCTGCTGTGTCTG
NM_001163485	***Rpl30***	AGTCTCTGGAGTCGATCAACT	AGCCAGTGTGCATACTCTGTAG
NM_007475.5	***Rplp0***	TGCCACACTCCATCATCAAT	CGAAGAGACCGAATCCCATA
NM_009092	***Rps17***	ATGACTTCCACACCAACAAGC	GCCAACTGTAGGCTGAGTGAC
NM_009825	***Serpinh1***	TGGTGACCCGCTCCTATACT	CCATCTGCAGCTTCTCCTTC
NM_007707.3	***Socs3***	CCTCGGGGACCATAGGAG	GGAAACTTGCTGTGGGTGAC
NM_011443	***Sox2***	CTCTGCACATGAAGGAGCAC	CTCCGGGAAGCGTGTACTTA
NM_011446	***Sox7***	CTTCAGGGGACAAGAGTTCG	GGGTCTCTTCTGGGACAGTG
NM_011441.5	***Sox17***	CTTTATGGTGTGGGCCAAAG	GCTTCTCTGCCAAGGTCAAC
NM_177753	***Sox21***	GCCCAGGAGAACCCTAAGAT	GTAGTCGGGATGCTCCTTCA
NM_011898	***Spry4***	GGTTCGGGGATTTACACAGA	CATGACTGAGCTGGGATTCA
NM_009309	***T***	CTGCGCTTCAAGGAGCTAAC	CCACTCCCCGTTCACATATT
NM_011535	***Tbx3***	CCTTCCACCTCCAACAACAC	GCATGCTGTTCAAATTGAGG
NM_011562.2	***Tdgf1***	GTTTGAATTTGGACCCGTTG	GTACGGCAGGTTCTTTCTGG
NM_009370.3	***Tgfbr1***	TTGGGACTTGCTGTGAGACA	TGCATAGATGTCAGCGCGTT
NM_023755	***Tfcp2l1***	CCGCCCCTACAGTATGTGTT	AGCCGGATTTCATACGACTG
NM_172472	***Tfe3***	TCATCCCCAAGTCCAATGAT	AGCGTTGCTGTTCCTTCTGT
NM_009523	***Wnt4***	ACTGGACTCCCTCCCTGTCT	GTCACAGCCACACTTCTCCA
NM_009556.3	***Zfp42***	CTGGGTACGAGTGGCAGTTT	CGTGTCCCAGCTCTTAGTCC
NM_001160251	***Zfp281***	TTCACCTCTCCACAACCACA	AACAGACTGGCCAAAACCAC
NM145492	***Zfp521***	CTGCAGAACCACATCCAGAC	TGGATGTCCCACTCGTTGTA
NM_001100186.1	***Zscan4d***	GGACAAAGAGGTGAGGTGGA	GTGAAGCCATTGTGGTGACA

Gata6ex1 primers were used in the WT-Ref analyses, while *Gata6ex4-5* were used in the mutant lines.
** Primers used to check the cell genotype.

Supplementary Table 5: List of antibodies used

TARGET	HOST	DILUTION	REFERENCES
CATENINb	Rabbit	1/300	C2206 (Sigma)
CDX2	Mouse	1/1	Ab86949 (ABCAM)
DUSP4	Rabbit	1/100	Ab216576 (ABCAM)
pERK	Rabbit	1/50	4370 (Cell Signaling Technology)
GATA6	Goat	1/300	AF1700 (R&D systems)
KLF4	Rabbit	1/300	GTX101509 (GeneTex)
LaminB	Mouse	1/100	Sc-374015 (SANTA CRUZ)
NANOG	Rabbit	1/100	Ab80892 (ABCAM)
NANOG	Rat	1/100	14-5761-80 (eBioscience)
OCT4	Rabbit	1/300	Sc-9081 (SANTA CRUZ)
OCT4	Rabbit	1/600	Ab19857 (ABCAM)
PDGFRα	Goat	1/100	AF1062 (R&D systems)
PECAM1	Goat	1/300	AF3628 (R&D systems)
SOX2	Goat	1/100	Sc-17320 (SANTA CRUZ)
SOX17	Goat	1/100	AF1924 (R&D systems)
SOX21	Goat	1/100	AF3538 (R&D systems)

Supplementary Table 6: List of genes used for PCA analyses of mouse and human cells

gene.id.mouse	gene.symbol	gene.id.human	gene.symbol
**ENSMUSG00000021835**	*Bmp4*	**ENSG00000118777**	*ABCG2*
**ENSMUSG00000021796**	*Bmpr1a*	**ENSG00000125845**	*BMP2*
**ENSMUSG00000074365**	*Crxos*	**ENSG00000125378**	*BMP4*
**ENSMUSG00000022150**	*Dab2*	**ENSG00000107779**	*BMPR1A*
**ENSMUSG00000024868**	*Dkk1*	**ENSG00000153071**	*DAB2*
**ENSMUSG00000031530**	*Dusp4*	**ENSG00000107984**	*DKK1*
**ENSMUSG00000022012**	*Enox1*	**ENSG00000120875**	*DUSP4*
**ENSMUSG00000021255**	*Esrrb*	**ENSG00000120658**	*ENOX1*
**ENSMUSG00000050917**	*Fgf4*	**ENSG00000138792**	*ENPEP*
**ENSMUSG00000031565**	*Fgfr1*	**ENSG00000119715**	*ESRRB*
**ENSMUSG00000030849**	*Fgfr2*	**ENSG00000175832**	*ETV4*
**ENSMUSG00000037025**	*Foxa2*	**ENSG00000244405**	*ETV5*
**ENSMUSG00000021944**	*Gata4*	**ENSG00000121769**	*FABP3*
**ENSMUSG00000005836**	*Gata6*	**ENSG00000075388**	*FGF4*
**ENSMUSG00000030117**	*Gdf3*	**ENSG00000077782**	*FGFR1*
**ENSMUSG00000021095**	*Gsc*	**ENSG00000066468**	*FGFR2*
**ENSMUSG00000024986**	*Hhex*	**ENSG00000136574**	*GATA4*
**ENSMUSG00000020679**	*Hnf1b*	**ENSG00000141448**	*GATA6*
**ENSMUSG00000020644**	*Id2*	**ENSG00000184344**	*GDF3*
**ENSMUSG00000038793**	*Lefty1*	**ENSG00000133937**	*GSC*
**ENSMUSG00000012396**	*Nanog*	**ENSG00000152804**	*HHEX*
**ENSMUSG00000021848**	*Otx2*	**ENSG00000275410**	*HNF1B*
**ENSMUSG00000029231**	*Pdgfra*	**ENSG00000115738**	*ID2*
**ENSMUSG00000020717**	*Pecam1*	**ENSG00000127528**	*KLF2*
**ENSMUSG00000024406**	*Pou5f1*	**ENSG00000136826**	*KLF4*
**ENSMUSG00000042414**	*Prdm14*	**ENSG00000171872**	*KLF17*
**ENSMUSG00000053113**	*Socs3*	**ENSG00000111057**	*KRT18*
**ENSMUSG00000074637**	*Sox2*	**ENSG00000171345**	*KRT19*
**ENSMUSG00000025902**	*Sox17*	**ENSG00000243709**	*LEFTY1*
**ENSMUSG00000061517**	*Sox21*	**ENSG00000081479**	*LRP2*
**ENSMUSG00000024427**	*Spry4*	**ENSG00000111704**	*NANOG*
**ENSMUSG00000018604**	*Tbx3*	**ENSG00000165588**	*OTX2*
**ENSMUSG00000032494**	*Tdgf1*	**ENSG00000134853**	*PDGFRA*
**ENSMUSG00000026380**	*Tcfcp2l1*	**ENSG00000261371**	*PECAM1*
**ENSMUSG00000051176**	*Zfp42*	**ENSG00000204531**	*POU5F1*
**ENSMUSG00000090714**	*Zscan4d*	**ENSG00000147596**	*PRDM14*
		**ENSG00000197956**	*S100A6*
		**ENSG00000184557**	*SOCS3*
		**ENSG00000181449**	*SOX2*
		**ENSG00000164736**	*SOX17*
		**ENSG00000125285**	*SOX21*
		**ENSG00000187678**	*SPRY4*
		**ENSG00000184292**	*TACSTD2*
		**ENSG00000135111**	*TBX3*
		**ENSG00000241186**	*TDGF1*
		**ENSG00000135457**	*TFCP2*
		**ENSG00000115112**	*TFCP2L1*
		**ENSG00000179059**	*ZFP42*
		**ENSG00000180532**	*ZSCAN4*
