# Supplementary material for: NANOG initiates epiblast fate through the coordination of pluripotency genes expression
Source: Nat Commun. 2022 Jun 21;13:3550. doi: 10.1038/s41467-022-30858-8 (PMC9213552; doi:10.1038/s41467-022-30858-8)
Supplement: Supplementary file 3 — Description of Additional Supplementary Files [file 41467_2022_30858_MOESM3_ESM.pdf]

Description of Additional Supplementary Files

Supplementary Data 1:
Single-cell gene expression analysis by RTqPCR in 104 cells at the 13C, 32C, 64C and 90C stages (n=44genes) or at the 32C, 64C and 90C stages (n=20 genes).
The edges of the box represent the 25th and 75th quartiles. The median is represented by the central line. The whiskers extend to 1.5 times the interquartile range (25th and 75th percentile). Cells are plotted individually.

Supplementary Data 2:
Single-cell gene expression analysis by RTqPCR in Fgf4+ and Fgf4- cells (n=104 cells at the 13C, 32C, 64C and 90C stages). For some genes, analysis at the 16C was not carried out.
WT (n=5), *N-/-* (n=5), *G-/-* (n=8) and *DKO* (n=5). The median is represented by the central line. The whiskers extend to 1.5 times the interquartile range (25th and 75th percentile). Cells are plotted individually.
See Supplementary Data 15 for statistical analysis.

Supplementary Data 3:
Correlation analyses of single-cell RNAseq data in mouse and human

Supplementary Data 4:
Spearman correlation matrix for gene expression analysis using RNA-seq data at 32C stage (Vector-based PDF). See Figure 1f.

Supplementary Data 5:
Spearman correlation matrix for gene expression analysis using RNA-seq data at 16C stage (Vector-based PDF). See Supplementary Fig 4b.

Supplementary Data 6:
RTqPCR analysis of whole individual WT, *Nanog*$^{-/-}$ (*N-/-*), *Gata6* $^{-/-}$ (*G-/-*) and *DKO* ICMs or embryos. Left panels: gene expression in individual WT embryos at the 8C (n=3) and 16C (n=3) stages and in individual ICMs at the 32C (n=5) and 90C (n=6) stages. Middle and right panels: gene expression in 32C *WT* (n=5), *N-/-* (n=5), *G-/-* (n=8) and *DKO* (n=5) and in 90C *WT* (n=6), *N-/-* (n=6), *G-/-* (n=5) and *DKO* (n=4) ICMs. The mean is represented by a square, the median by the central line and outliers are plotted individually. The edges of the box represent the 25th and 75th quartiles. The whiskers extend to 1.5 times the interquartile range (25th and 75th percentile). expression levels are relative to the mean level in 32C wt samples.
See Supplementary Data15 for statistical analysis.

Supplementary Data 7:
Single-cell gene expression analysis by RTqPCR in *WT* (n=24), *N-/-* (n=15), *G-/-* (n=13) and *DKO* (n=24) from ICM at the 32C-50C.
See Supplementary Data15 for statistical analysis.

Supplementary Data 8:
List of human single cells used in Figure 4.

Supplementary Data 9:
Spearman correlation matrix for paired expression of the 308 *FGF4*-correlated genes in the 258 human ICMd cells (see Supplementary Data 3). Genes are ordered in a hierarchical tree for similarity (Vector-based PDF of Figure 4d).

Supplementary Data 10:
Spearman correlation matrix for the paired expression of 1052 genes (selected by their correlation with *NANOG* expression in ICMd, see Supplementary Data 3) in ICMp cells. (Vector-based PDF of Supplementary Figure 12a).

Supplementary Data 11:
Spearman correlation matrix for the paired expression of 1052 genes (selected by their correlation with *NANOG* expression in ICMd, see Supplementary Data 3) in ICMd cells. (Vector-based PDF of Supplementary Figure 12b)

Supplementary Data 12:

Spearman correlation matrix for the paired expression of the 308 genes from Fig 2a (selected by their correlation with *FGF4* expression in ICMd, see Supplementary Data 3) in ICMp cells. (Vector-based PDF of Supplementary Figure 12c)

Supplementary Data 13:
Spearman correlation matrix for the paired expression of 1065 genes selected from Striparo et al. (see Supplementary Data 3) in ICMd. (Vector-based PDF of Supplementary Figure 12d).

Supplementary Data 14:
Spearman correlation matrix for the paired expression of 1065 genes selected from Striparo et al. (see Supplementary Data 3) in ICMp. (Vector-based PDF of Supplementary Figure 12e).

Supplementary Data 15:
Statistical values (RT-qPCR experiments)
