# Supplementary figures and images for: NANOG initiates epiblast fate through the coordination of pluripotency genes expression
Source: Nat Commun. 2022 Jun 21;13:3550. doi: 10.1038/s41467-022-30858-8 (PMC9213552; doi:10.1038/s41467-022-30858-8)

Supplementary data1.

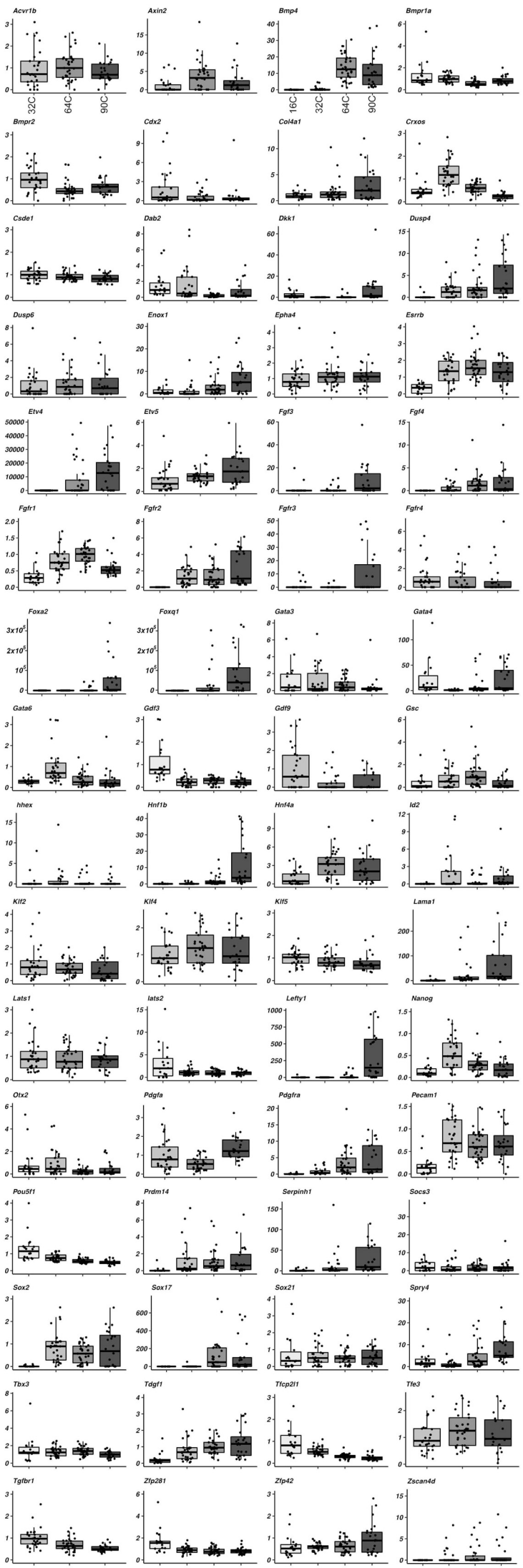

Supplement: Supplementary file 4 — Supplementary Data 1 [file 41467_2022_30858_MOESM4_ESM.pdf]

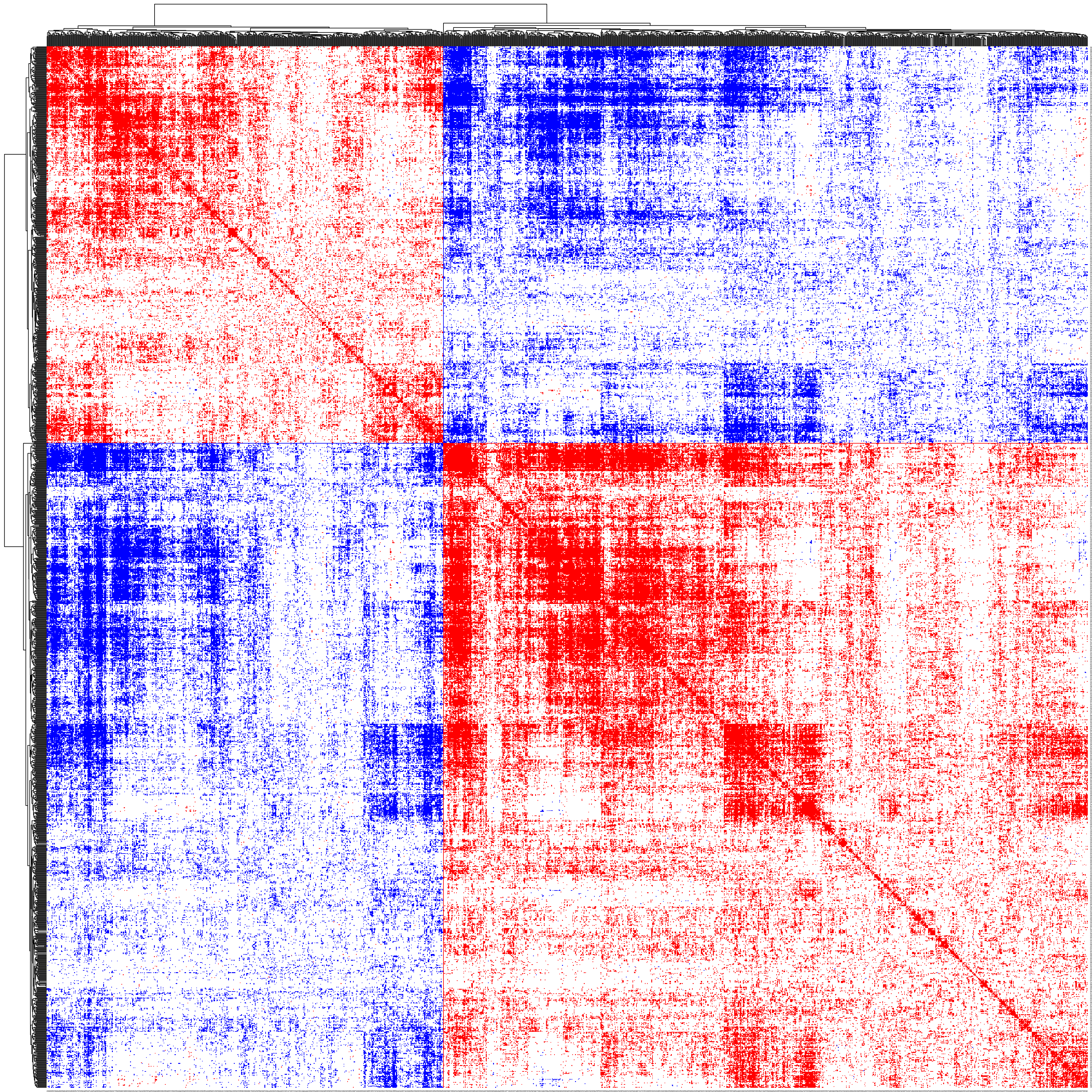

Supplement: Supplementary file 7 — Supplementary Data 4 [file 41467_2022_30858_MOESM7_ESM.pdf]

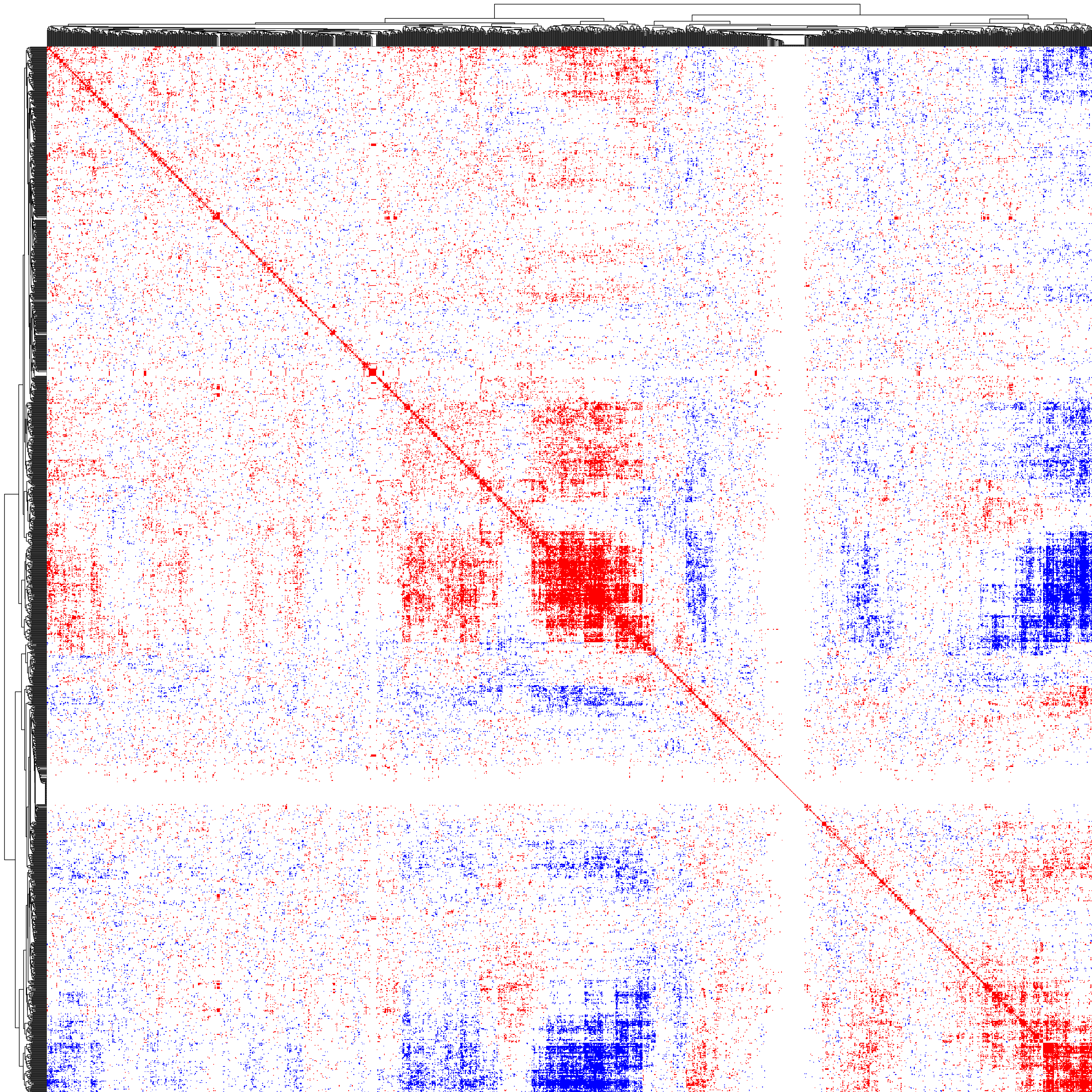

Supplement: Supplementary file 8 — Supplementary Data 5 [file 41467_2022_30858_MOESM8_ESM.pdf]

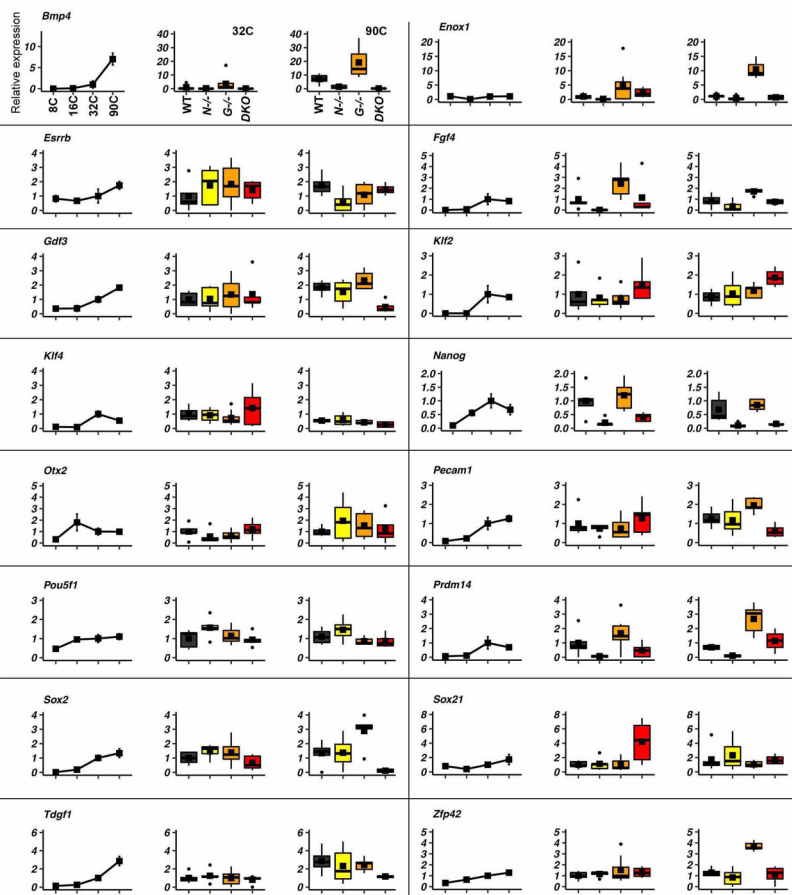

## PrE genes expression

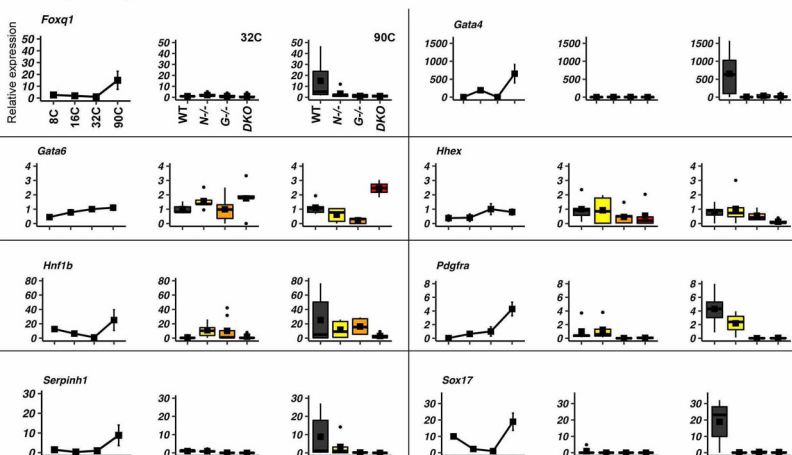

## FGF pathway genes expression

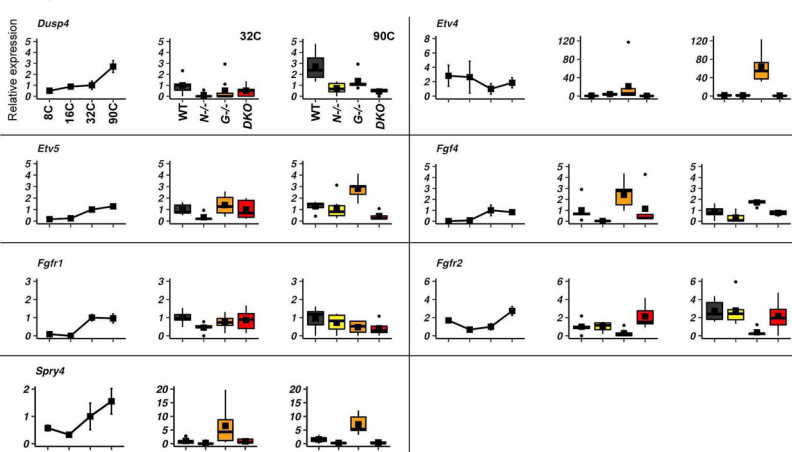

## Other genes expression

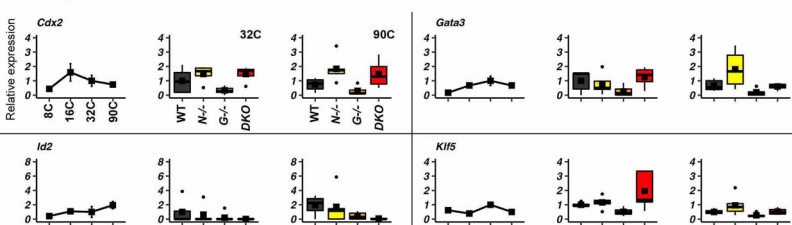

Supplement: Supplementary file 9 — Supplementary Data 6 [file 41467_2022_30858_MOESM9_ESM.pdf]

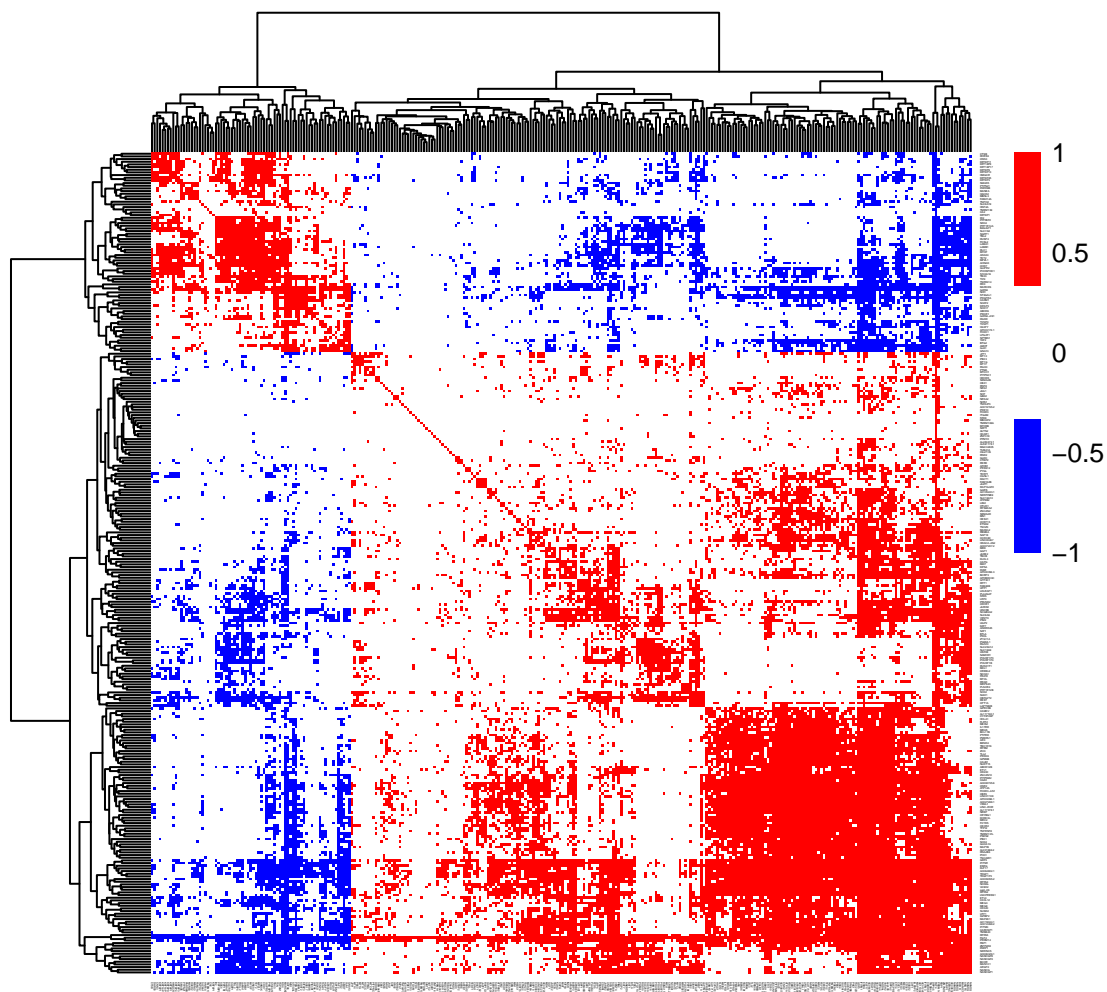

Supplement: Supplementary file 12 — Supplementary Data 9 [file 41467_2022_30858_MOESM12_ESM.pdf]

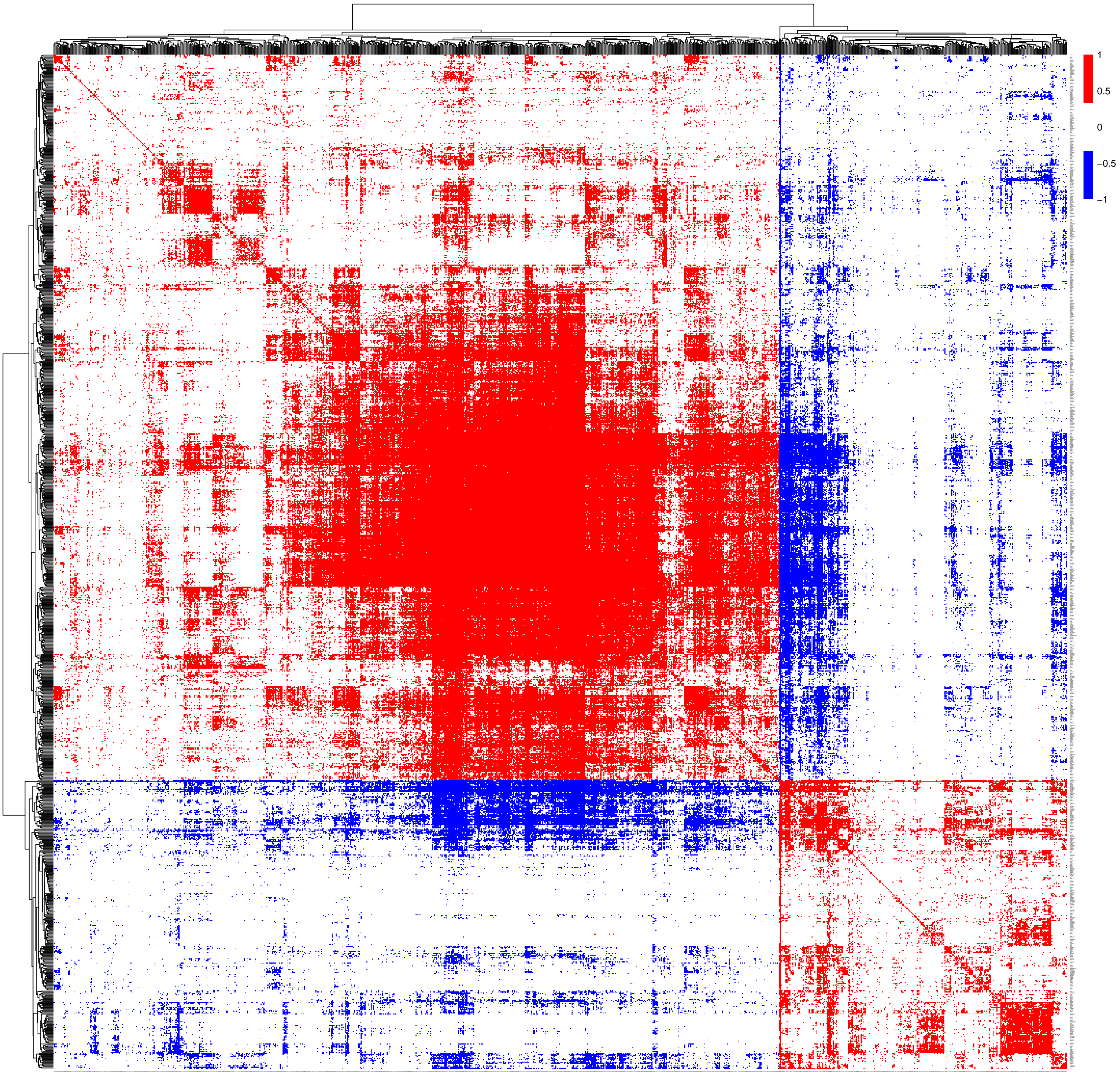

Supplement: Supplementary file 13 — Supplementary Data 10 [file 41467_2022_30858_MOESM13_ESM.pdf]

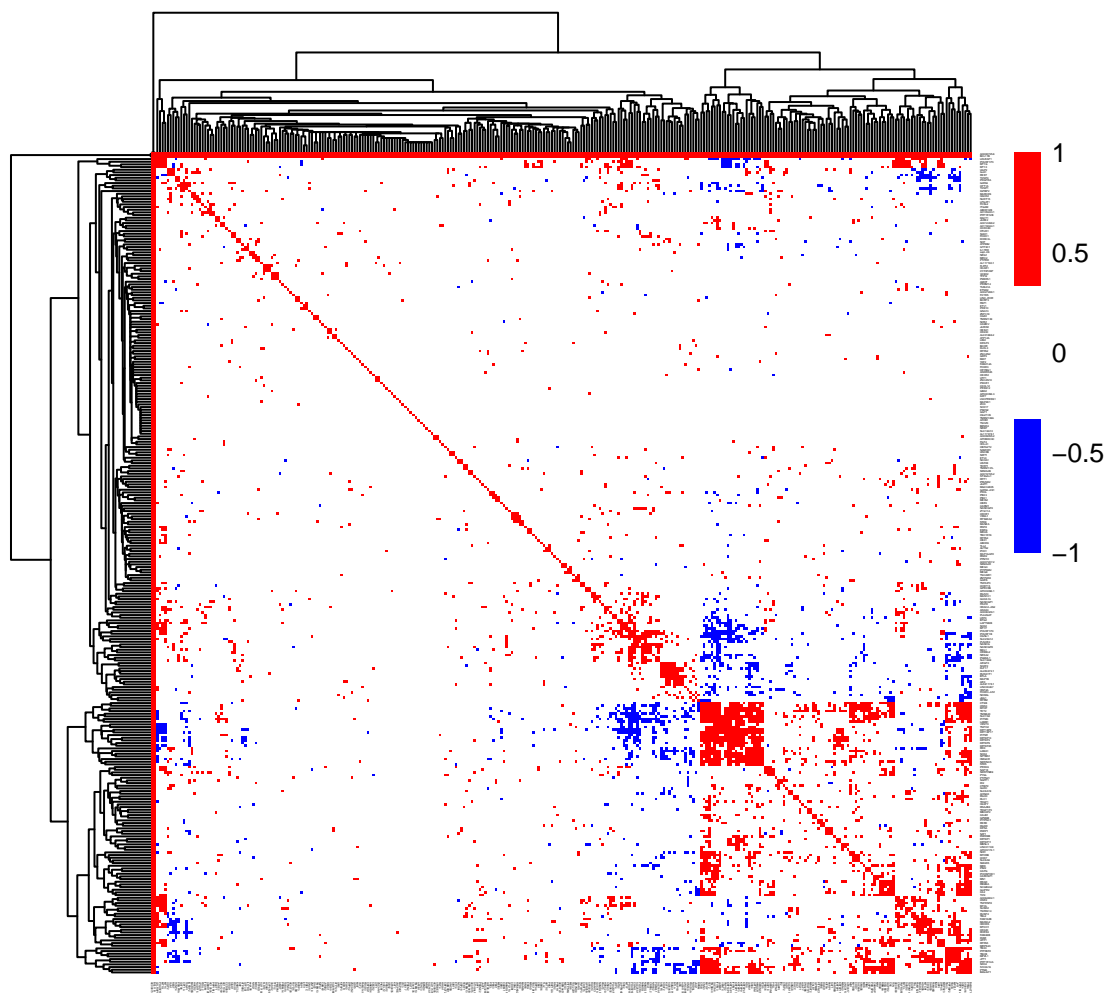

Supplement: Supplementary file 14 — Supplementary Data 11 [file 41467_2022_30858_MOESM14_ESM.pdf]

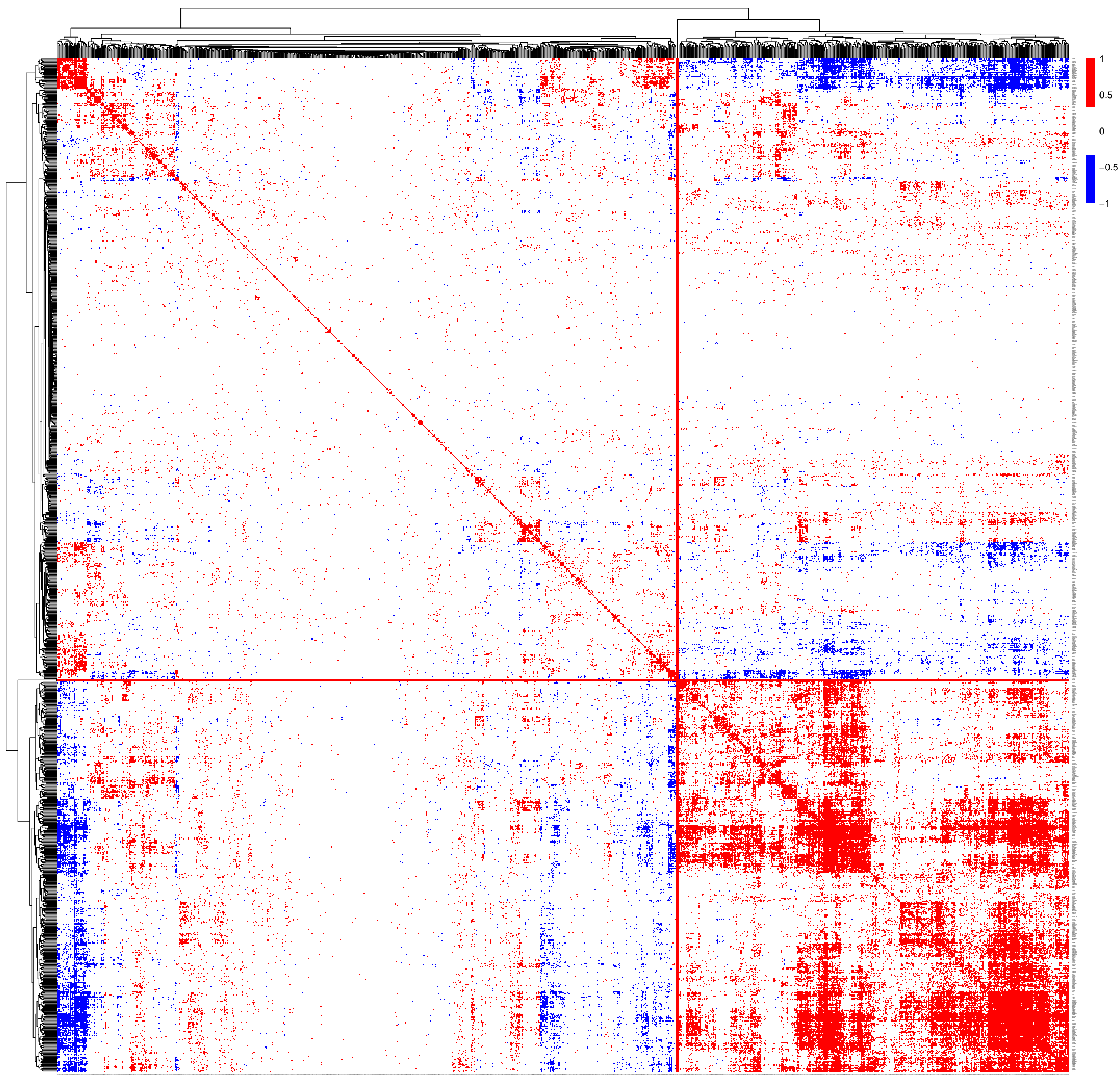

Supplement: Supplementary file 15 — Supplementary Data 12 [file 41467_2022_30858_MOESM15_ESM.pdf]

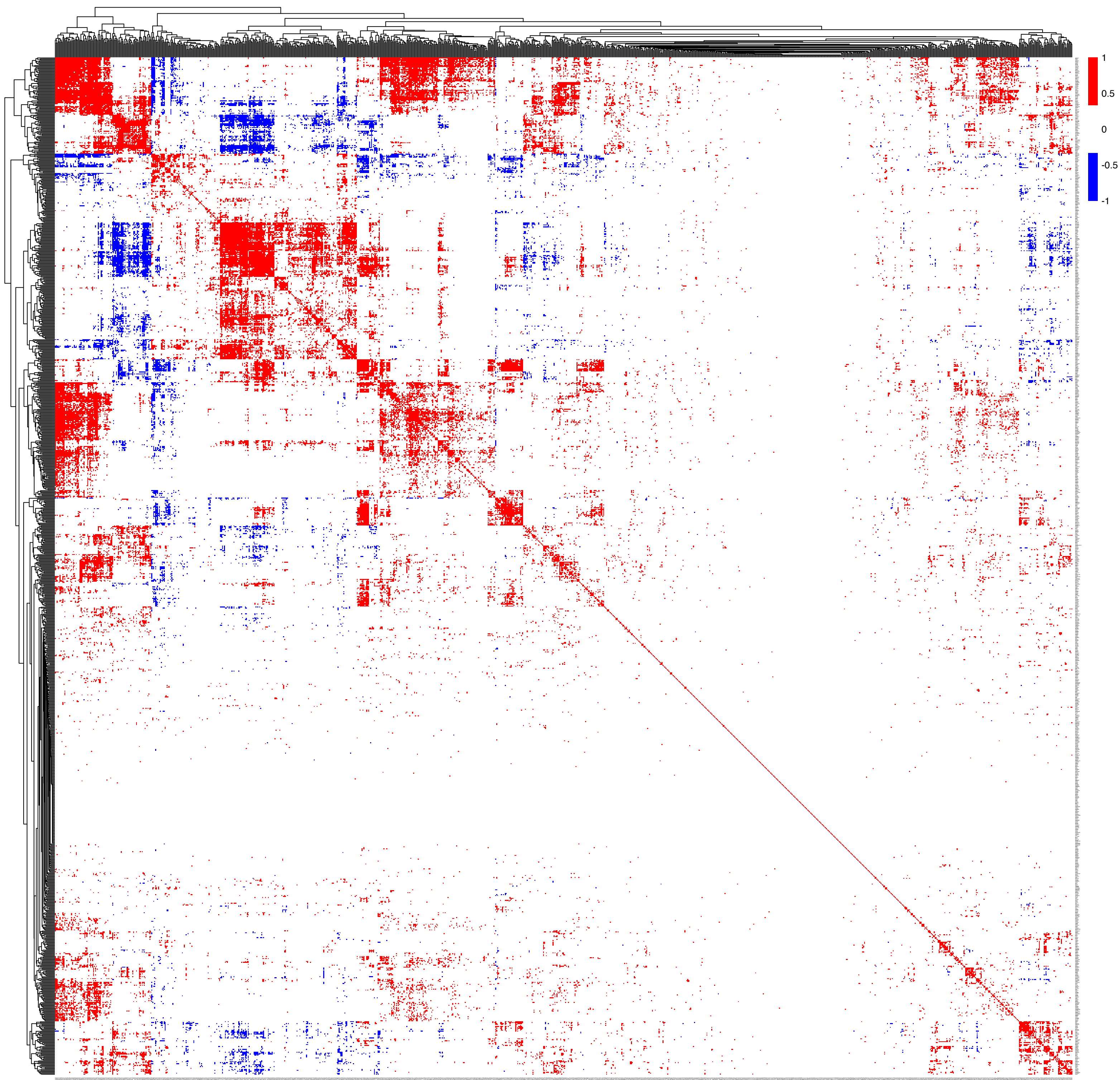

Supplement: Supplementary file 16 — Supplementary Data 13 [file 41467_2022_30858_MOESM16_ESM.pdf]

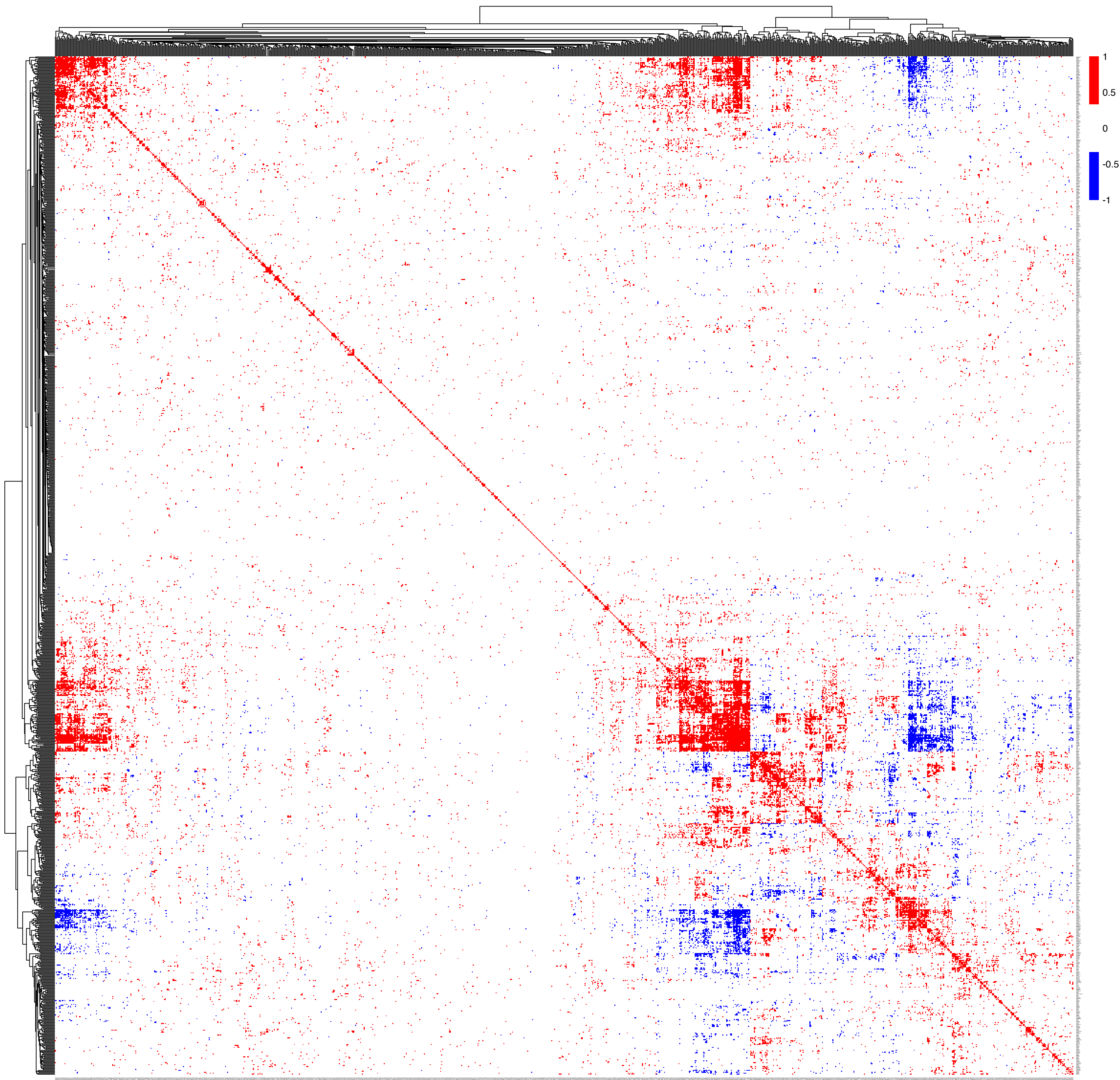

Supplement: Supplementary file 17 — Supplementary Data 14 [file 41467_2022_30858_MOESM17_ESM.pdf]
