# Supplementary material for: NANOG initiates epiblast fate through the coordination of pluripotency genes expression
Source: Nat Commun. 2022 Jun 21;13:3550. doi: 10.1038/s41467-022-30858-8 (PMC9213552; doi:10.1038/s41467-022-30858-8)

Supplementary data 2

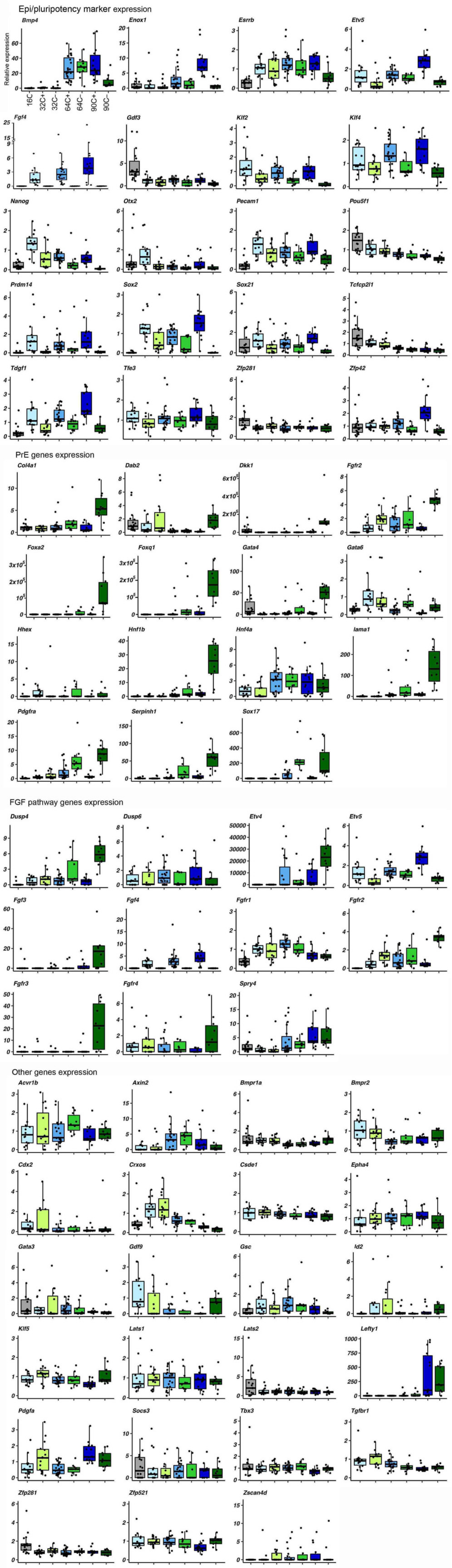

Supplement: Supplementary file 5 — Supplementary Data 2 [file 41467_2022_30858_MOESM5_ESM.pdf]
