# Supplementary material for: NANOG initiates epiblast fate through the coordination of pluripotency genes expression
Source: Nat Commun. 2022 Jun 21;13:3550. doi: 10.1038/s41467-022-30858-8 (PMC9213552; doi:10.1038/s41467-022-30858-8)

Supplementary data 7

Epi/pluripotency markers expression

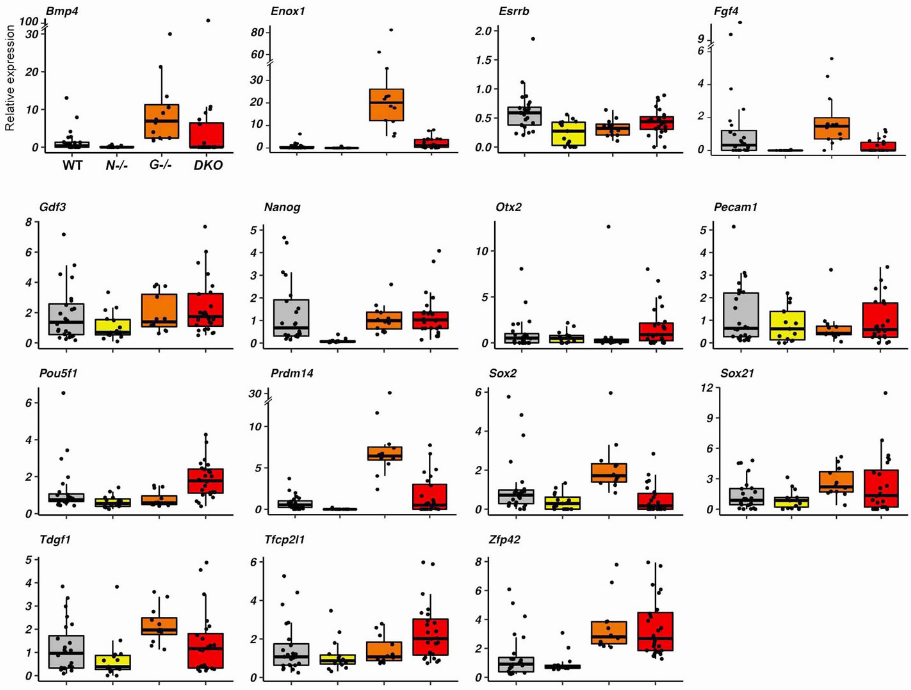

PrE genes expression

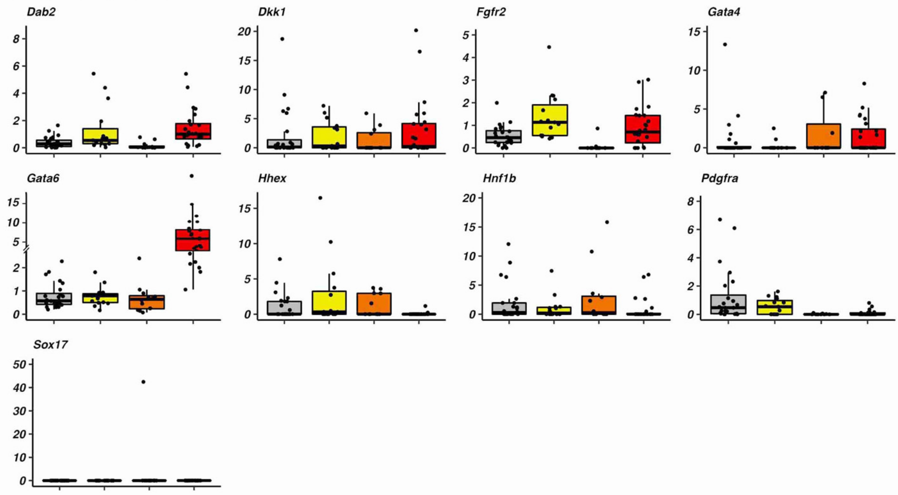

FGF pathway genes expression

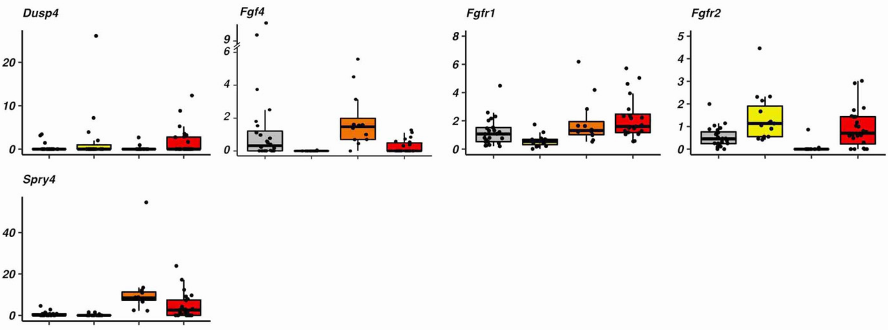

Other genes expression

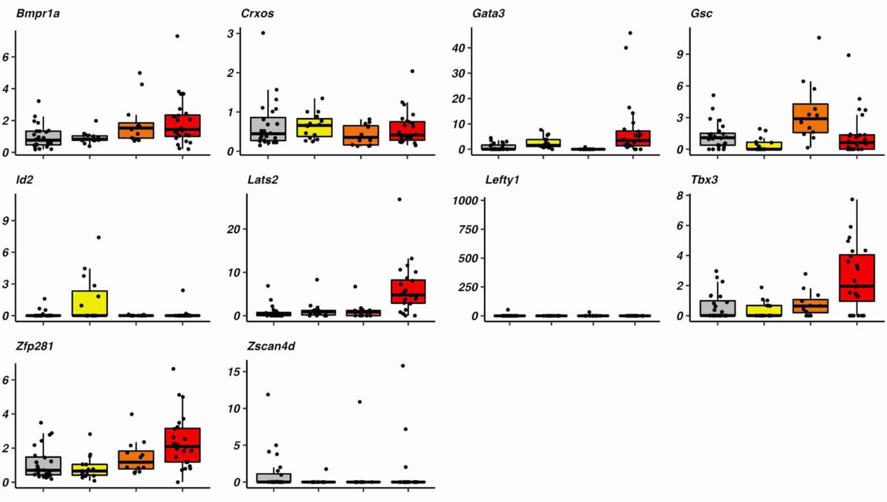

Supplement: Supplementary file 10 — Supplementary Data 7 [file 41467_2022_30858_MOESM10_ESM.pdf]
