# Supplementary material for: NANOG initiates epiblast fate through the coordination of pluripotency genes expression
Source: Nat Commun. 2022 Jun 21;13:3550. doi: 10.1038/s41467-022-30858-8 (PMC9213552; doi:10.1038/s41467-022-30858-8)
Supplement: Supplementary file 2 — Peer Review File [file 41467_2022_30858_MOESM2_ESM.pdf]

nature portfolio

Peer Review File

UrsNANOG initiates epiblast fate through the coordination of pluripotency genes expression.

Open Access This file is licensed under a Creative Commons Attribution 4.0 International License, which permits use, sharing, adaptation, distribution and reproduction in any medium or format, as long as you give appropriate credit to the original author(s) and the source, provide a link to the Creative Commons license, and indicate if changes were made. In the cases where the authors are anonymous, such as is the case for the reports of anonymous peer reviewers, author attribution should be to 'Anonymous Referee' followed by a clear attribution to the source work. The images or other third party material in this file are included in the article's Creative Commons license, unless indicated otherwise in a credit line to the material. If material is not included in the article's Creative Commons license and your intended use is not permitted by statutory regulation or exceeds the permitted use, you will need to obtain permission directly from the copyright holder. To view a copy of this license, visit http://creativecommons.org/licenses/by/4.0/.

The previous round of reviews was completed at another journal

Answers to referees comments on the original manuscript submitted to *Nature*.

Referee #1 (Remarks to the Author):

Review of Allegre et al.

NANOG and GATA6 are known to regulate EPI and PrE divergence in mice and the senior author has published a number of important papers investigating the expression of these transcription factors and their relationship to FGF signalling. This is an interesting paper and is an advance over previous publications investigating the relationship between FGF signalling and EPI/PrE differentiation and the expression of NANOG/GATA6. The most interesting findings are that ICM heterogeneity is initiated independently of NANOG, GATA6 and FGF/ERK signalling (with evidence of the heterogeneous expression of SOX21 and SOX2, though seemingly not for PrE markers). In addition, the authors showed that FGF signalling is downstream of EPI specification, which depends on NANOG expression. Initial heterogeneity in TF expression in the ICM cell is further reinforced by FGF signalling that depends of NANOG.

While I believe the findings will be of interest to developmental biologists and even some stem cell biologists, I'm unsure of its broader interest to others outside of the field, but perhaps this reflects the writing style that makes it difficult to access. The paper is generally difficult to follow and the writing and figures needs to be improved. The authors might want to consider generating a schematic model, especially when discussing the correlation with Fgf4 expression.

We thank the reviewer for these instructive comments. We have broaden the scope of the manuscript by adding a novel *in silico* single-cell analysis of human embryos. This not only widens the impact of the results but also reinforces the data as similar cell transitions in cell heterogeneities can be found in both mice and humans, suggesting a conserved mechanism. We show that correlation analyses offer an efficient method to segregate undifferrentiated from differentiated cells in the ICM in both mouse and human.

Upon reviewers comments, and also due to novel results to include, we have reorganised the structure of the article. In addition, we have generated a schematic model as suggested.

We believe that the manuscript will be of interest not only to developmental biologists but also to stem cell biologists working on pluripotent stem cells. Indeed, showing how the pluripotent Epi state is initiated/established will tell them where pluripotent cells are coming from. Moreover, the correlation analysis method we show here can provide a read out to isolate cells equivalent to the ICM state, as no *in vitro* model of the ICM state has been found yet (Posfai, *Nature Cell Biology* (2021) **23**, 49–60; Stirparo et al, 2019).

I think if the authors could go some way to determine the evolutionary conservation of their findings (as is hinted in the abstract and discussion) or further uncover the currently unknown partner(s) functioning independently of the FGF pathway or speculate about which factors might initiate this early heterogeneity this would increase the impact of the paper. For example, what factors are known to regulate Sox2 expression? How might the heterogeneity in Sox2 and Sox21 impact on Fgf2 and subsequent Nanog expression?

We thank the reviewer for these suggestions.

- As mentionned above we are demonstrating an evolutionary conservation of cell states transition between mouse and human.
- We also analysed potential partners to NANOG. SOX2, KLF4, NANOG and OCT4 interact and synergise in ES cells to maintain the naïve pluripotency state. In the embryo they are expressed independently of the FGF pathway in ICM cells before Epi differentiation. Using Proximity Ligation Assays (PLA) we now provide evidences that NANOG is crucial for a maximal interaction between SOX2/OCT4 and SOX2/KLF4 duos. This is in line with findings in ES cells showing that NANOG recruits transcription factors to target genes involved in ES cell maintenance (Heurtier et al, 2019).

- SOX2 presence is regulated, at least, by the Hippo pathway from the 16C stage, to restrict it to ICM cells versus TE cells (Wicklow, 2014). Active (nuclear) YAP is homogeneously low in early ICM cells (Hashimoto, Dev Cell 2019, PMID: 31204175), thus the hippo pathway does not seem to influence cell heterogeneity in the early ICM. The extracellular matrix/Integrinb1 may also have an influence on ICM specification/SOX2 expression at the 16C stage (Biorxiv doi.org/10.1101/2021.07.05.450860). However no difference was observed when the number of Epi and PrE cells was analysed in *Itgb1* mutants. The Initiation of *Sox2* expression is independent from NANOG and OCT4 activities but they are required to maintain it (Frum et al., 2019; this manuscript). SOX2 is homogeneously present at low levels in 4C-8C stage blastomeres (White et al, 2016), despite a possible influence of heterogeneous SOX21 (Goolam et al, 2016). Thus *Sox2* expression initiation remains unknown.

Sox2 mutation does not impact NANOG expression during Epi specification. In *Sox2* mutants Epi and PrE specification occurs correctly as illustrated by the mutually exclusive expression of NANOG and GATA6 expression in early blastocysts (Wicklow et al, 2014) but PrE further differentiation is delayed due to a lower production of FGF4 (*Fgf2* is not expressed in the blastocyst, Ohnishi, 2014) by Epi cells (Avillion et al, 2003; Wicklow et al, 2014). Is SOX2 only required after Epi specification to produce FGF4 or does it have a role earlier in a redundant manner with other factors remains an open question.

- SOX21 has been shown to be heterogeneously expressed at 4C, much earlier than ICM differentiation. Still would the early 4C expression have an impact later? RNAi experiments show that SOX21 depletion does not impact on Epi and PrE proportions (Goolam et al, 2016). This also means that SOX21 probably does not have any impact *Fgf4* expression.

Thus results in the literature reported so far do not show any impact of SOX2 or SOX21 on NANOG expression during Epi specification.

The continued expression of Prdm14, Sox2 and Klf2 in the NANOG/GATA6 DKO embryos indicates that the EPI may still be initiated despite the loss of NANOG, so I think the wording here needs to be considered carefully. Experimentally this could be determined, for example by disaggregation/chimera experiments to investigate cell autonomous versus non-cell autonomous effects of the loss of NANOG/GATA6. Are the DKO cells still responsive in an otherwise normal environment and can they at all continue to contribute to the developing embryo?

We apologize for the lack of clarity in our previous manuscript. One of the discoveries of our manuscript is that the transition from an uncoordinated to a coordinated expression of Epi factors defines the switch from one cell state (progenitor) to another (differentiated Epi). Some Epi genes are expressed in both states but their patterns of expression are clearly different. There are thus two clearly distinct states that need to be called differently. Therefore when uncorrelated, the expression of pluripotency markers is not sufficient to define the Epi pluripotent state.

In the DKO, *Prdm14, Sox2, Klf2* as well as other Epi genes remain expressed however their expression is not correlated in the same cells, meaning that the Epi identity has not been acquired.

NANOG non cell-autonomous role in Epi differentiation is an additional question. NANOG is a transcription factor and has been recently shown to recruit pluripotency factors to target Epi genes in mES cells (Heurtier et al, 2019), thus NANOG activity in ES/Epi cells is at least cell autonomous. Whether NANOG can induce a non cell-autonomous factor that then could rescue the coordination of Epi genes expression in absence of NANOG remains unknown. To analyse Epi differentiation of DKO chimeric cells in a WT embryo, single cell RNA analyses would have to be carried out to see whether coordinated expression of Epi genes is present or not. While theoretically feasible, the 1/16 DKO embryos would make this experiment extremely difficult. Due to the scarcity of DKO embryos we focused on other experiments.

Interestingly, the authors show that an EPI transcriptional program may be initiated between the 16-32-cell stages. It is unclear if this is also at the protein level for any factor other than Sox2/Sox21. Did the authors find any other interesting candidate genes?

Other reports as well as our previous work has shown that several proteins coded by genes identified in the Epi signature such as KLF4 (Morgani et al, 2015), TDGF1 (Fiorenzano et al, 2016), PECAM1 (Robson et al,2001), ETV5 (Azami et al, 2019), Rex1/ZFP42 (Climent et al, 2013 doi: 10.1089/scd.2012.0211) start to be detected or become specific to Epi cells during Epi/PrE differentiation. Thus, these are Epi markers at both RNA and protein levels.

The heterogeneous expression of Fgf4 is associated with the expression of Nanog, Klf2 and Klf4, among other factors. Given that Gata6 is not similarly co-expressed at earlier stages the authors conclude that Nanog is required for the high expression of Fgf2 and that EPI specification drives the downstream FGF pathway by upregulating FGFs above a level required for PrE differentiation. Could this speculation be confirmed experimentally by genetic or pharmacological means?

Two independent labs have shown that defined levels of FGF4 are critical to induce PrE differentiation. This was shown both genetically, using combinations of heterozygous, embryos with zygotic or maternal zygotic deletion of *Fgf4*, and pharmacologically by administrating recombinant FGF4 to *Fgf4* KO embryos. (Kang, 2011; Krawchuk, 2011). In addition, it was recently shown that FGF4 levels, depending on Epi cells, drive the pattern and the proportions between Epi and PrE populations (Saiz et al, 2019; Fischer et al, 2020) and are directly mediated by ERK signalling (Simon et al, 2020). We show that activated ERK as well as DUSP4 are absent in DKO embryos demonstrating that the low levels of *Fgf4* expression are not sufficient to induce the FGF pathway.

In the DKO embryos Fgf4 and the receptors Fgfr1 and Fgfr2 are expressed, but downstream target genes were downregulated. Based on this downregulation of a few putative target genes and the pERK staining in Figure 1 (which is unconvincing) the authors conclude that FGF signaling is not activated. But this dampened expression is not confirmed experimentally. The use of other methods, such as FISH, which the senior author developed ought to be considered to determine if their hypothesis of globally reduced expression compared to heterogeneous expression with some cells expressing these genes above a certain threshold holds true. An alternative experimentally better but more difficult method is to use the Fgfr1 and Fgfr2 reporter mice used in this paper: https://www.ncbi.nlm.nih.gov/pmc/articles/PMC5502766/
Since the first submission of this manuscript we have published the dynamics of pERK during blastocyst formation and demonstrated that DUSP4 is also a good reporter of pERK activity (Azami et al, 2019).
We have replaced the image showing pERK staining as rightly suggested (now Extended data Figure 5g).
We have carried out a new FISH protocol (the previous one reffered by the author was not compatible with precise quantification). These novel experiments confirm the lower expression of *Fgf4* in DKO cells (Fig 3c,d).
So we demonstrate a dampened FGF pathway by four means: lower range of *Fgf4* expression in single cells by RTqPCR and by FISH, absence of pERK and absence of DUSP4 in the ICM.
The Fgfr1 and Fgfr2 reporter mice suggested by the reviewer are transcriptional reporters that reflect gene expression and not the activation of the receptors. This would not bring any added value to our RNA analysis.

What is the significance of the continued Fgfr2 expression in the DKO embryos? Are the embryonic cells in the DKO embryos still responsive to FGF signalling activation – e.g. do they upregulate Sox17 and/or Gata4 in the presence of exogenous FGF?
In DKO embryos *Fgfr2* expression probably does not depend on FGF pathway activation as we show that this pathway is inactive in these ICMs. The continued expression of *Fgfr2* in DKO embryos could be due to the absence of NANOG, as indeed *Fgfr2* transcripts are also high in *Nanog* single mutants.
Others and we have shown that in absence of *Gata6,* SOX17 or GATA4 cannot be expressed, even with exogenous presence of recFGF2/4 (Schrode et al, Dev Cell 2014; Bessonnard et al, Development 2014).
As asked by the reviewer we have performed embryo cultures with recFGF2/4 as we

previously did (Frankenberg et al, Dev Cell 2011; Bessonnard et al, Development 2014; Azami et al Development 2019). Immunofluorescence did not reveal the presence of SOX17 or PDGFRA in DKO embryos in the presence of recFGF(n= 3/3). This result is not included in the manuscript that is focused on progenitor to Epi transition.

SOX2 and SOX21 are initially heterogeneously expressed indicating that EPI is initiated and that heterogeneity of early markers is independent of NANOG initiation. This observation seemingly contradicts the statement on line 63: “Altogether, our gene expression analyses revealed that DKO ICM cells cannot differentiate into Epi or PrE.” I instead interpret initiation of SOX2 and SOX21 (and upregulation of Klf2 and continued expression of Prdm14) as indicating partial initiation of EPI differentiation in the DKO embryos, though the maturation of this lineage may be compromised.
SOX2, SOX21, Klf2, Klf4 are indeed expressed in *DKO* embryos, however not in the same cells. As in 16C embryos, their expression is not coordinated. This shows that the Epi state is not reached in *DKO* embryos and that the cells remain in a progenitor state, bearing the primary heterogeneity characterized by uncorrelated expression of some pluripotency markers. The individual presence of these markers does not define the Epi state. For example, some of these markers are already present before the 4C stage (Klf2, Klf4, Pecam1, Sox2; Guo et al, 2010), in blastomeres that indeed are not Epi cells.

In extended data figure 1 c and e for the DKO embryos – is there a hole in the ICM with missing cells? Is this abnormality in the ICM consistently seen and what is the explanation?
Blastocele cavity formation can be initiated with two cavities, in WT embryos as well. We have changed the images to avoid confusion.

Figure 2d is identical to one of the panels in Extended data figure 2c – the authors should not reuse images or figures in the paper and each panel needs to be unique.
We chose to put all the analysed genes altogether on the same supplementary figure to enable a better comparison.

Could the authors provide a list of all of the genes used to analyse figure 2 as a supplementary table?
We are now providing the list in the Extended data table 6.

In Supplementary Figure 4 – Otx2, Pou5f1 and Zfp42 expression seems higher in the DKO embryos compared to wildtype controls. Could the authors perform statistical tests of all of the genes in this figure and report which ones are significantly different from wildtype ICMs and how they interpret this data? Why is Gata6 higher in the DKO ICM data shown in this figure?
The statistical data were provided in SI 8, which is now SI 15.
Indeed, *Gata6* RNA levels are high in DKO embryos, and suggest a complex regulation of transcription as it is low in *Gata6* and *Nanog* single mutants. We have no explanation so far.

Line 137-138 and Figure 2 what does +/- ve mean? The authors need to better definite any acronyms used. In the methods section they state that “Cells were considered in

Fgf4–ve subpopulations when CT values were above 35 and all other cells were placed in Fgf4+ve subpopulations." But I'm confused does ve reflect a cell type or that the cell is uncommitted?

We have made changes accordingly, with *Fgf4*+ and *Fgf4*- being abbreviations of *Fgf4*-expressing and *Fgf4*-non expressing cells respectively.

In the following datasets: 348032_0_supp_3185139_ppcc0f and 348032_0_supp_3185140_ppcc0f these heatmaps are not useful in their current form. The text is not visible. It would be better to actually visualise the text or to reduce this figure so it can be visible.

These files are vectorized PDF (SI 4, 5, 9-14) that enable zooming without pixellisation. Not all PDF applications are suitable to enable correct zooming. In our hands Acrobat Reader DC, freely available, works well. It enables to zoom in to read genes names and genes search is possible.

Figures are frequently not called out in text in the order that they are shown in the figures and these need to be reorganized.

We are doing our best to call out figures in the order and to have coherent figures. We have made some changes to better organize figures and hope this has improved the manuscript.

Could they provide a table with the spearman p values calculated for the same genes evaluated in extended data figure 3c?

We have added the p-values accordingly.

For clarity in the figure and figure legends N-/- should be spelled out as Nanog-/- and G-/- as Gata6-/-.

We apologize but we are using abbreviations for space matters and readability.

Lines 18 and 36: missing the word "development" after preimplantation

It has been added accordingly

Lines 121 and 163 single-cell RNA analyses – what type? Seq or qRT-PCR?

The types of experiments are indicated in the figures legends.

Line 41: need to state what the PrE cells form for non-specialist readers of the journal

We are now giving this information accordingly.

Line 47 the hypothesis should be the selection of one of two options not "with or without cell heterogeneity". Is it one and why select this option? Or if it wasn't part of the original hypothesis then leave it off.

We rephrased the text accordingly.

In figure 1: could they spell out control in the black boxes – there is room to fit it in. As above, the expression of pERK is unconvincing and the staining method needs to be improved. There also needs to be-co-staining for a PrE protein to determine the cell type. In the figure legend it states that "The asterisk labels a mitotic cell" but there is no

asterisk.
For consistency, we spell out the controls the same way in all the figures, and in some there is not enough space to write it fully. Since the first submission, we have demonstrated that pERK staining is present in Gata6-expressing cells (Azami et al, 2019). We have corrected the figure's legend.

Figure 2: panel h need to correct the comma to a dot.
We have made corrections accordingly

Esrrb is a better example compared to Bmp4 that is currently in Figure 2f.
Accordingly we have added Esrrb, and other genes, in the novel Fig 1e, as Seurat plots allows visualisation of more genes

Figure 3: panel d need to correct the comma to a dot.
We have made corrections accordingly

Figure 4: Difficult to see the quantitative plots comparing expression. Could the authors select fewer examples in the main figure and place the other panels in the supplementary data and further improve visibility by making the panels larger? The figure call out on lines 212-213 described Dab2 and Lrpap1 anti-correlation with Fgf4, but this data is not shown. Would an R2 value provide additional evidence of correlation/anti-correlation?
We have made changes in the organisation of figures. Some panels have been transferred to the supplementary data or removed for space matters. We hope that this new organisation will be suitable.

On line 124 and in figure extended data figure 2a is this hierarchical clustering of the hand-picked subset of genes? If so, I think this needs to be stated more clearly.
The results for this figure were redundant with the PCA analysis, which is more informative. Therefore, we have removed it.

Lines 192 and 202: should not be Fgf4's, instead change to Fgf4 expression.
We have made the correction.

Referee #2 (Remarks to the Author):

In this study, Allègre, Chauveau et al. investigated the origin of heterogeneity in the inner cell mass (ICM). Using Nanog and Gata6 double knockout (DKO) mouse embryos and single-cell expression analysis to characterize ICM precursor states, they concluded that Nanog is indispensable for the initiation of epiblast (Epi) specification at the 16/32-cell stage. The investigated topic is timely and very important for the scientific community interested in the mechanism of embryonic lineage specification, yet the data presented in the manuscript appear however to be of preliminary rather than commanding value. The correlative findings are unfortunately weak, and the provided insights are of unsatisfactory significance. The reviewer has listed following examples to

illustrate the need for further investigation:

The conclusion that Nanog is exclusively required for the high expression of FGF4 in individual cells or the early ICM is not sufficiently justified. As FGF4 expression is correlated with the expression of many pluripotency genes, the reviewer wonders why the authors put their focus solely on Nanog? Downregulation (by siRNA or dCas9) of other correlated factors is required to establish Nanog's role. These experiments can also identify the timing and the need for the expression of particular genes that are responsible for Epi and primitive endoderm (PrE) formation.
We thank the reviewer for these comments, that helped us to improve the clarity of the manuscript. We also think that NANOG does not act alone to induce EPI differentiation. However as far as we know, NANOG is the only Epi/pluripotency factor whose inactivation induces a phenotype defective for Epi initiation.
Several loss of function experiments have been carried out on Epi/pluripotency factors that have a correlated expression at 32C. In addition to *Oct4* and *Sox2 (*Avilion, G&D 2003; Nichols, Cell 1998; Wicklow, Plos Biol 2014; Frum, Dev Cell 2013; Le Bin, Development 2014; Stirparo et al, PNAS 2021, PMID: 33452132), several genes such as *Tdgf1, Klf2, Klf4, Sox21, Zfp42 or Esrrb*, have no early embryonic phenotypes when mutated or down regulated (Ding et al, Nature 1998, PMID: 9790191; Masui et al, BMC Dev Biol 2008 PMID: 18433507; Goolam et al, 2016; Luo et al, Nature 1997, PMID: 9285590), even *Klf2;Klf4* double KO survive to E10.5 (Chiplunkar et al, BMC Dev Biol. 2013, PMID: 24261709). If these factors have a role to induce Epi cell differentiation, it would be masked by redundancy. Alternatively possible maternal expression could rescue a phenotype, however this is not the case for *Sox2* and *Oct4* whose MZ-mutants still properly specify Epi and PrE cells (Wicklow et al, 2014; Frum et al, 2013).

To explore how co-factors could interact with NANOG, we have carried out Proximity Ligation Assays (PLA). Our results show that NANOG co-factors such as SOX2, OCT4, KLF4 preferentially interact (between themselves and NANOG) in Epi cells. Our results also show that NANOG is required for maximal PLA signals (Fig.3 g,h).

The presence of heterogeneity in DKO cells (in which Nanog is absent) indicates that the emergence of the Epi lineage is not necessarily related to Nanog expression. However, while the authors acknowledge this result, they still conclude that Nanog (potentially with a co-factor) could be the source of heterogeneity. To provide a comprehensive understanding and a truly significant mechanistic insight, the identification of a co-factor would strenghten the manuscript tremendously.
Our results uncover that there are two different states of ICM heterogeneity, and we rewrote the manuscript to describe this more clearly. Before Epi specification, ICM cells show cell-to-cell heterogeneity for some Epi markers expression, however the expressions of these different genes are not coordinated. On the reverse, the coordination of Epi markers expression defines the onset of Epi specification. Thus some Epi genes are expressed in both states but their patterns of expression are clearly different.
Therefore, the mechanism for Epi induction results in the transition from uncoordinated to coordinated gene expression heterogeneity. We show that *Nanog* is absolutely required for this transition and thus for the emergence of the Epi lineage.

In addition, PLA experiments indicate that NANOG is required for a maximal interaction between SOX2, KLF4 and OCT4. These results are in agreement with experiments in mES cells demonstrating that NANOG recruits pluripotency transcription factors at genes enhancers to establish/maintain the naive pluripotent mES state (corresponding to an Epi state) (Heurtier et al, 2019). We have thus brought novel mechanistic elements to understand the emergence of Epi specification:
- ICM progenitor cells show uncoordinated expression of Epi/pluripotency factors
- the Epi state is induced through the coordinated expression of these factors
- NANOG is required for these factors expression coordination
- NANOG is required to enable SOX2, KLF4 and OCT4 maximal interactions

The authors claim that Nanog's function is essential already between the 16/32-cell stages for the initiation of Epi specification. A systematic single-cell analysis of gene expression differences at the 16-cell stage between WT and DKO embryos to evaluate whether apparent gene expression differences already exist at the 16-cell stage will strengthen the narrative of the manuscript. If it is the case, then these differences could also contribute to cell allocation within the ICM.

Most of Epi genes that are expressed at 16C in WT embryos remain expressed in 32C *DKO* embryos with a similar pattern (i.e. uncoordinated expression, Extended data Table 2 and 3). Thus *Nanog* mutation does not prevent their expression at the 32C stage, it only prevents expression coordination during the transition process. This is further supported by PLA experiments (Fig 3 g, h). Thus, as being present in 32C DKO ICMs, these early Epi genes are most likely expressed in 16C DKOs.

The focus of our manuscript is the establishment of the Epi state. We think that examining DKO embryos at the 16C stage is not required to validate the major findings of our manuscript:
- the Epi state is determined by the coordinated expression of Epi/pluripotency markers from 16/32C stage.
- NANOG is required for the transition from random to coordinated expression of Epi/pluripotency genes.
- a primary ICM heterogeneity occurs in absence of NANOG, GATA and FGF pathway
- Cells states (ICM progenitors, Epi, PrE) exist in human similarly to in mouse.

As cells in the ICM of DKO embryo do not differentiate to Epi and PrE, are these in the ICM of DKO embryos at late blastocyst stages more similar to 32 cell stage WT embryos?

The DKO embryo analyses that we have carried out at late blastocyst stage (90C stage) were through either whole ICM RTqPCR analyses or immunofluorescence. Both analyses show that Epi and PrE cells cannot differentiate in DKO embryos. Thus the phenotype is not a delay in differentiation.

Currently, the authors only compare expression levels of individual genes such as Sox2 and Sox21 (in very few embryos, n=2 for Sox21). This analysis deserves a more systematic approach. In general, a lot of conclusions are drawn from immunostainings, yet the number of replicates for these stainings are often very low or not mentioned at all.

We apologize for the low number of embryos in the first manuscript. It has been difficult

and long to work with double mutant embryos as only 1/16 embryo are DKO (explaining in part the delay in the resubmission). We have increased these numbers. Of note, all phenotypes are fully penetrant.

We believe that our manuscript results from a strong combination of immunostainings that enable protein analyses at the single cell level and of single-cell RNA analyses. During this study, we have analysed DKO embryos using several markers by different methods:
- 35 markers by RTqPCR in the whole ICM, including 16 Epi markers (validated as such by published articles)
- 68 markers by RTqPCR on single cells, including 18 genes considered as Epi or pluripotency markers
- protein analyses of 9 markers by immunofluorescence, including 4 Epi markers. We have added KLF4 immunostaining to this study.

The manuscript lacks sometimes clarity: some sentences are very long and convoluted. In addition, the figures should have titles that summarize the findings, and the axes of all plots should be labelled properly.
We have carefully revised the manuscript to improve its clarity and fixed figure labelling. The previous and present manuscripts have been through english corrections by professionals.

Minor comments:
• The generation of DKO lacks detail. The authors say that no Nanog mRNA could be detected, but could mRNA be produced in the first place?
The generation of individual mutations for *Nanog* and *Gata6* are referenced. DKO embryos were obtained from double-heterozygous intercrosses as individual homozygotes are embryonic lethal. For some experiments, we used Nanog$^{+/-}$; Gata6$^{Flox/Flox}$; ZP3::Cre females to increase the number of DKO embryos. We have added these details in the methods section.
Nanog RNA can be detected in *Nanog* mutant and DKO embryos as we used primers upstream of the mutation. The *LacZ* chimeric RNA produced by the mutated allele is stable and can be translated, as shown by the ß-gal staining in heterozygous embryos (Mitsui et al, 2003).

• It is not clear whether Sox2 and Pecam1 are also heterogeneous at the 32-cell stage. In other words, does Nanog heterogeneity precede Sox2 heterogeneity?
Sox2 and *Pecam1* RNAs are clearly enriched in Epi cells. It is difficult to assess PECAM1 protein differential levels as it is present at membranes. Thus adjacent membranes cannot be distinguished with classical confocal microscopy and the amount of Pecam1 in each cell cannot be precisely assessed. Nevertheless going through the image z stack and examining cells that are on the outside of the ICM (thus not sharing membrane with another ICM cell), different levels can be appreciated between ICM cells. SOX2 is heterogeneously present at the 32C stage with the 2 antibodies we have been using (Goat from Santa-Cruz, Mouse MAB2018 from R&D). Co-staining with these two antibodies provides the same labelling variations in each cell. In this study we only used

the goat antibody for consistency.
Before blastocyst formation, NANOG starts to be detected at the 8C stage in a heterogeneous pattern, (Dietrich and Hiiragi, 2007; Plusa et al, 2008). SOX2 levels are low and homogeneous before the 16C stage and increase drastically in inner cells from the 16C stage with cell to cell variability (White et al, 2016; Wicklow et al, 2014). Thus expression patterns would indicate that NANOG heterogeneity appears before SOX2 heterogeneity. However, removing NANOG does not prevent SOX2 heterogeneous expression before blastocyst formation (this manuscript; Frum et al, 2019) and NANOG remains expressed correctly in *Sox2* mutants (Wicklow et al, 2014).

• Figure 3: it is confusing to plot different time points for the WT-reference cells while for the KO cells only cells of the 32-cell stage are displayed.
We chose to plot *DKO* cells (that were collected at the 32C stage) against all WT-reference stage to be able to see where these cells are situated on the plot compared to the others. This enables to see that *DKO* cells are not delayed compared to the WT

• The lack of p-ERK signaling in N-/- embryos does not necessarily mean that it is a direct effect of Nanog. Could this be tested in ESCs?
ChIP experiments in ESCs have shown that NANOG can bind *Fgf4* locus (Chen, Cell 2008; Marson et al, Cell. 2008 134: 521–533; Gagliardi et al, EMBO J. 2013 ;32: 2231–2247). Thus induction of *Fgf4* transcription by NANOG is mostlikely direct in the embryo.

• Figure 4B: Please provide a correlation coefficient for each plot.
We have reorganised several figures and removed these plots. Correlation coefficients between genes are found in table SI 3.

• The candidate transcription factors found to be correlated with FGF4 expression at the 32-cell stage are indicative of the acquisition of an Epi identity. The correlation plots also show that these genes correlate with Nanog expression. What if the analysis is turned around i.e first look for candidate TFs that are correlated with Nanog in the ICM and then look if they also express high Fgf4?
On the reviewer's suggestion, we are now showing the correlations values with *Nanog* expression (Ext data fig 3b). They demonstrate that a coordinated expression of Epi genes with that of *Nanog* is present at the 32C stage but not at the 16C stage.

• The authors rightfully state that the FGF pathway is less important for differentiation into Epi and PrE in other mammalian embryos. Therefore, heterogeneities that appear before those in Nanog and FGF could be more critical for lineage allocation in the ICM. If this is the case, then what is the exact impact of the current findings?
There are indeed heterogeneities that appear before those of *Fgf4* (thus before 32C) and of NANOG (thus before 8C) heterogeneities. For example, SOX21 and CARM1 have been shown to be heterogeneously expressed at 4C (Torres-Padilla et al, 2007; Goolam et al, 2016), a much earlier stage. Would this have an impact later? RNAi experiments show that SOX21 RNAi does not impact on Epi and PrE proportions (Goolam et al, 2016). Carm1 levels (Torres-Padilla et al, 2007; Goolam et al, 2016, White et al, 2016) impact *Sox2* and *Nanog* mRNA levels at 8C stage by RNAi or mRNA injection. Carm1 MZ-KO (Hupalowska et al, 2018) have decreased cell numbers in the

three cell lineages (embryos are arresting around the 32C) preventing cell lineage analyses at the blastocyst stage.

Nevertheless the question refers to the impact of early (4C or 8C) heterogeneities on the primary heterogeneity at 16C or the later Epi/PrE heterogeneity. If *Nanog* and *Sox2* (or other Epi marker) expression are directly inherited by 4C heterogeneities, their expression should be coordinated at the 16C stage. However, *Nanog* and *Sox2* expressions are not correlated at 16C stage demonstrating that Epi specification is not directly inherited from one or two cells at the 4C stage.

If upstream pathway(s) or transcription factor(s) such as CARM1 can influence some Epi factors, their activity(ies) would have to impact each Epi gene differently as their expression is not correlated in 16C stage inner cells. Still, increasing or decreasing the levels of a key Epi factor could shift the probability of this key factor to be co-expressed with other Epi factors in individual cells. Thus the number of Epi cells could change depending on upstream pathways/factors influence. However the mechanism of Epi specification depends on the occurence of Epi genes co-expression in the same cell. Thus despite possible deterministic components, the mechanism remains stochastic.

We have modified the text to better explain this part.

• Both “SI” and “extended data” are used to refer to supplemental figures. Only a uniform term should be used throughout the manuscript.

We are strictly following Nature guidelines for authors.

Referee #3 (Remarks to the Author):

In this manuscript by Allegre and colleagues, the authors investigate mechanisms driving specification of epiblast (EPI) and primitive endoderm (PrE) cells in the inner cell mass (ICM) of the mouse blastocyst. This topic is significant as it provides the basis for understanding the molecular regulation of pluripotency and pluripotent stem cells. The earliest event that is known to induce differentiation of the ICM to EPI and PrE fates is FGFR/ERK signaling, which dozens of high profile studies have shown to be necessary and sufficient to induce PrE differentiation. However, this manuscript provides the first evidence of ICM heterogeneity that is upstream or independent of FGFR/ERK signaling. Thus, if true, this manuscript would present a MAJOR breakthrough in the field.

Overall, the authors have undertaken experiments that are extremely technically challenging. Their results are truly surprising and potentially ground breaking since all heterogeneity within the ICM is thought to be dependent on FGFR/MEK signaling. The evidence for an FGFR/MEK-independent is quite striking. What is less strong is the interpretation and mechanistic explanation, particularly of the epistasis (double knockout) experiments. In other words, the major shortcoming of this draft is in the writing and not in the experimentation. With a few exceptions described below, the studies are experimentally rigorous. However, the authors simply describe phenotypes and fail to provide mechanistic interpretations of these elegant genetic studies. There is no integrative molecular model to propose: what do NANOG and GATA6 do in which cells. This will prevent this study from moving the field as far forward as it could and should. For example, the authors refer to “NANOG/GATA6 ratio” controlling gene expression and cell fates in several places in the paper. A more

mechanistic explanation would be more like, NANOG represses GATA6 in epiblast cells, which would otherwise represses targets X, Y, Z in epiblast cells, etc.. Put another way, how could a “ratio” be interpreted by a cell? No mechanism is proposed.

We thank the reviewer for the supportive comments. We have rewritten the manuscript to better describe the proposed mechanism and we have included a schematical model.

Additional Concerns:

1) Please avoid using “+ve” and “-ve” to indicate positive and negative. These are not well-defined conventions.
As requested, we have modified abbreviations to “+” and “-“ for positive and negative, respectively

2) Line 49 and extended data Fig. 1a do not adequately validate that TE and ICM are properly segregated because the images are extremely dim, and there is no quantification.
We apologize for the poorly contrasted image. In the new figure we have added another picture and CDX2 labelling is shown with levels of grey that enable a better visualisation of the staining (now ext data fig 5a,b).

3) For the readers clarity and ease of understanding, Fig. 1b should be edited to include labels under each graph.
For space matters due to the novel results, these data are no longer in the main figures but have been moved to SI 6.

4) On line 62, the authors indicate that “absence of Nanog RNA in DKO embryos indicated that NANOG is required for its own expression in embryos.” However, this is not a valid interpretation since both Nanog and Gata6 are absent in the DKO embryos. The authors would have to look in Nanog knockouts to draw conclusions about Nanog’s roles. There are similar logical fallacies on lines 98-103 and 181-184.
NANOG protein is absent in DKO embryos, however the first exons are present on the mutated allele. The truncated allele can be transcribed, visualised by ß-gal staining in heterozygous embryos (Mitsui et al, Cell 2003). This enables to detect the transcription of the gene by RTqPCR in absence of the protein and provides information on the transcriptional status.

5) The conclusion that DKO cells remain as ICM precursors (lines 83-86) is short on evidence because SOX2 and NANOG exhibit heterogeneity with similar timing (Wicklow et al., 2014) and because little is known about the timing of SOX21 heterogeneity in the ICM.
The fact that Epi markers expressions at the RNA level are not correlated in DKO at 32C stage reveals that cells have not acquired an Epi state. In addition, by examining genes preferentially expressed at higher levels at 16-32C (Ext data Fig 7), and down-regulated at subsequent stages, we show that these markers remain expressed at a high level in DKO embryos, supporting a freeze in the progenitor state.
Indeed to our knowledge, SOX21 protein heterogeneity and down regulation has not

been characterized so far in the blastocyst. This is thus novel data that we are unveiling in the manuscript, providing one of the first protein marker for undifferentiated cells.

6) In line 71, when describing SOX2 and PECAM1 expression, the number of replicates in unclear. As in the main text it says 32C (n=4) and 90C (n=7); however, in the cited Fig 1c, and extended fig. 1d legends indicate different replicates. Extended fig. 1d indicates an n=2 for PECAM1, does not explicitly indicate what stage. Fig. 1c indicates an n=6 for SOX2, stating they are 90C stage. I would recommend clarification of replicate number.
As recommended we are now indicating the details for each protein at each stage.

7) While DKO SOX21 PECAM1, and ETV5 phenotypes appear to be fully penetrant, replicates should be increased as n=2 is low.
We apologize for these low replicate numbers in the first manuscript. We have increased the numbers of DKO embryos examined accordingly and the phenotypes are still fully penetrant

8) Fig. 2c, d, b, Fib. 3a, c, and Fib. 4b, c – the dots are so small it is nearly impossible to resolve differences in color.
Increasing the dot size would prevent to distinguish them easily from each other. We have changed the colours of some graphs for a better visualisation.

REVIEWER COMMENTS

Reviewer #2 (Remarks to the Author):

In this manuscript the authors advanced their previous analysis strengthening their observation that ICM heterogeneity is upstream or independent of FGFR/ERK signalling. Importantly, the authors make an effort to provide a mechanistic explanation to the emergence of Epi specification using PLA which is very much appreciated. The schematic representation and discussion is thorough , yet I would encourage the authors to include original studies that could explain how Nanog ensures the maximal interaction of SOX2/OCT4 and SOX2/KLF4 rather than referring to reviews. For instance, partitioning error and trancriptional bursts (Shi et al., 2015, Dev) leading to differential transcription factor kinetics and nuclear presence (Plachta et al., 2011, NCB) could account for this change.

Overall, the written manuscript is much improved and the results are clearly presented. From the remarks I made on the original manuscript I found the responses sufficient and well thought out.

Reviewer #3 (Remarks to the Author):

I really wanted to like this manuscript, but it suffers several fatal flaws, unfortunately:

- Figures and text are extraordinarily disorganized, with an apparent stream of consciousness narrative. This could have been a desk reject.
- Much of the mouse embryo transcriptional analysis confirms known concepts of early development, yet the authors do not carefully position their observations in the context of the broader field. Thus, the first result section comes across as descriptive and incremental. Additionally, it is not clear how inside and outside cells are distinguished at the earliest stages. Finally, the main result is sort of a negative result: no strong correlations prior to the 32-cell stage - are the authors sure that they have the statistical power to make this conclusion? Regardless, the point has been made by many others before.
- The most interesting results are presented in the second result section (analysis of the Gata6 and Nanog DKO embryos). Yet, the analyses were not sufficiently rigorous to provide confidence in the conclusions. The observation that some pluripotency genes are expressed heterogeneously in the ICMs of DKO embryos even in the absence of FGF signaling - is potentially very significant and highly impactful. However, the authors fail to establish that the observed heterogeneity is the same as the wild type heterogeneity. There is no quantification; there is no evaluation of cell death; there is no evaluation of coexpression of the pluripotency genes.
- The third result section (PLA evaluation of NANOG/SOX2/OCT4 interaction) is also potentially interesting. However, it is unclear why, if SOX2 and OCT4 are pioneer factors, would they need NANOG in order to interact? Are the authors suggesting that these are not pioneer factors in this context?
- The fourth section is an analysis of human embryo transcriptional data. It is unclear what the significance of FGF signaling is in the human embryo is (functionally, none), which raises questions about the significance of expression differences along these lines.

Reviewer #4 (Remarks to the Author):

The authors have addressed this reviewer's comments; the revised manuscript is now clear and interesting. The addition of the new analysis of published human embryo transcription data introduces novel evidence of the evolutionary relevance of the original mouse-specific study.

Reviewer #2 (Remarks to the Author):

In this manuscript the authors advanced their previous analysis strengthening their observation that ICM heterogeneity is upstream or independent of FGFR/ERK signalling. Importantly, the authors make an effort to provide a mechanistic explanation to the emergence of Epi specification using PLA which is very much appreciated. The schematic representation and discussion is thorough , yet I would encourage the authors to include original studies that could explain how Nanog ensures the maximal interaction of SOX2/OCT4 and SOX2/KLF4 rather than referring to reviews. For instance, partitioning error and trancriptional bursts (Shi et al., 2015, Dev) leading to differential transcription factor kinetics and nuclear presence (Plachta et al., 2011, NCB) could account for this change.

Overall, the written manuscript is much improved and the results are clearly presented. From the remarks I made on the original manuscript I found the responses sufficient and well thought out.

We thank the reviewer for his/her support. We have included the references as suggested.

Reviewer #3 (Remarks to the Author):

I really wanted to like this manuscript, but it suffers several fatal flaws, unfortunately:

- Figures and text are extraordinarily disorganized, with an apparent stream of consciousness narrative. This could have been a desk reject.
Upon the reviewer's suggestion (and the other reviewers), we had reorganised the figures from the previous version. We have made our best for fitting figures with the editorial guidelines. We are happy to improve data organization if the reviewer/editor have insightful suggestions.

- Much of the mouse embryo transcriptional analysis confirms known concepts of early development, yet the authors do not carefully position their observations in the context of the broader field. Thus, the first result section comes across as descriptive and incremental.
We agree that existence of two phases of cell heterogeneity has been previously suggested by Ohnishi et al, 2014. However, our data bring important and novel insigths that are listed below: 1) Compared to previous publications (Ohnishi et al, 2014; Kang et al, 2017; Nowotchin et al, 2019), our ICM single-cell RNA analyses starts at an earlier stage (16C), thus before Epi differentiation. This enabled to observe the transition toward Epi differentiated cells. This allowed us to show that the switch from uncoordinated to coordinated expression occurs between 16C and 32C stages. This also provided a method to examine undifferentiated or differentiated cell states that we could implement in human embryos. 2) Our analyses distinguish for the first time subpopulations at 32C. Indeed, we demonstrate that several Epi/pluripotency markers are correlated at 32C and segregate on the PCA, validating an Epi state. 3) We also show that *Fgf4* expression is not randomly expressed but induced only in the cells that

have acquired this Epi state. This shows that *Fgf4* induction is posterior/concommitant to Epi specification.

Additionally, it is not clear how inside and outside cells are distinguished at the earliest stages.
As described on Fig1A and mat&Met, ICM were isolated by immunosurgery from the 32C stage while all cells from 16C embryos were processed and analysed. Concerning our *in silico* analyses from Posfai et al data, we only included 16C and 32C cells that were classified as inside cells according to *Cdx2-GFP* and other makers expression. Our results indicate that Epi markers are not coordinated in inside cells at the 16C stage.

Finally, the main result is sort of a negative result: no strong correlations prior to the 32-cell stage - are the authors sure that they have the statistical power to make this conclusion? Regardless, the point has been made by many others before.
Prior to the 32C stage there is an <u>absence</u> of correlation of Epi markers expression as we reported. This absence of correlation is not a negative result and illustrates a state different than the Epi state. This is the switch from uncorrelated to correlated expressions that defines the beginning of Epi formation. This is the first time that Epi gene expression correlation analyses are carried out on 16C inner cells.

- The most interesting results are presented in the second result section (analysis of the Gata6 and Nanog DKO embryos). Yet, the analyses were not sufficiently rigorous to provide confidence in the conclusions. The observation that some pluripotency genes are expressed heterogeneously in the ICMs of DKO embryos even in the absence of FGF signaling - is potentially very significant and highly impactful. However, the authors fail to establish that the observed heterogeneity is the same as the wild type heterogeneity.
PCA (Fig 3e) and correlation analyses (Supplementary Table 3) reveal a coordinated expression of Epi genes in WT ICMs at 32-50C while uncoordinated in *DKO* embryos at the same stage. This establishes that the observed heterogeneity between ICM cells is <u>different</u> in WT and *DKO* embryos. Therefore, *Nanog* and/or *Gata6* are important to coordinate expression of EPI and/or PrE genes.

There is no quantification; there is no evaluation of cell death; there is no evaluation of coexpression of the pluripotency genes.
We thank the reviewer to point at this and apologise for leaving this behind. We now show an ICM cell quantification in Supplementary Figure 5 (a''). There is no variation between the controls and *DKO* so cell death analyses were not carried out.
Correlation analyses of the expression of Epi/pluripotency genes in WT (correlation) and *DKO* (no correlation) are shown in Supplementary Table 3 (unchanged). This demonstrates that *Nanog* is required for gene expression coordination.

- The third result section (PLA evaluation of NANOG/SOX2/OCT4 interaction) is also potentially interesting. However, it is unclear why, if SOX2 and OCT4 are pioneer factors, would they need NANOG in order to interact? Are the authors suggesting that these are not pioneer factors in this context?
The precise mechanism is not known yet. Our results clearly show that NANOG is

required for an optimal interaction. We propose that NANOG enables the association of pluripotency factors to induce the Epi state based on this and on findings in ES cells, indicating that NANOG recruits pluripotency TF at binding sites. In the embryos, we do not know whether this is a direct recruitment (even if NANOG interacts with these factors) or through the pluripotency GRN which is activated concommitantly.
It has indeed been shown that pioneer factors can act differently depending on the context and can require co-factors (see Zaret, K.S. Pioneering the chromatin landscape. Nat Genet 50, 167–169 (2018); Zeitlinger, Curr Opin Syst Biol. 2020 Oct; 23: 22–31 and references therein). We have added a sentence in the discussion to mention this possibility.

- The fourth section is an analysis of human embryo transcriptional data. It is unclear what the significance of FGF signaling is in the human embryo is (functionally, none), which raises questions about the significance of expression differences along these lines.
Indeed, FGF function in human embryos is not completely clear yet, although a recent report shows that treating human embryos from 2 to 6 dpf impacts NANOG expression (Wamaitha et al, Nat Commun. 2020; 11: 764).
Nevertheless FGF signaling is not the subject of our analysis in human embryos, we only use it as an Epi marker, which is indeed very specific. If the reviewer was referring to the correlation analyses with *FGF4* expression, we also used a list of genes with expression correlations with *NANOG* expression (SI 12 a,b) as well as with a WGCNA based gene list (Supplementary figure 12 d,e). The three analyses show that there is a switch from uncoordinated to coordinated expressions between ICMp and ICMd cells.

Reviewer #4 (Remarks to the Author):

The authors have addressed this reviewer's comments; the revised manuscript is now clear and interesting. The addition of the new analysis of published human embryo transcription data introduces novel evidence of the evolutionary relevance of the original mouse-specific study.

We thank the reviewer for covering Reviewer 1 comments and his/her support.

REVIEWERS' COMMENTS

Reviewer #5 (Remarks to the Author):

Here, Allegre et al., use single-cell profiling, accompanied by orthogonal analysis to demonstrate that the epiblast state is defined between 16-32 cell stages, by the coordinated expression of Epi/pluripotency markers. This coordination fails in expression fails in Nanog and Gata6 double KO embryos. I have assessed the authors' responses to the concerns raised by previous reviewers. It is my assessment that the authors have addressed all the points raised. This is an interesting mechanistic study and the data also represents a valuable resource.
